# A global snapshot on health systems capacity for detection, monitoring, and management of acute kidney injury: A multinational study from the ISN-GKHA

**DOI:** 10.1371/journal.pgph.0003823

**Published:** 2024-10-15

**Authors:** Marina Wainstein, Yannick Nlandu, Andrea Viecelli, Javier A. Neyra, Silvia Arruebo, Fergus J. Caskey, Sandrine Damster, Jo-Ann Donner, Vivekanand Jha, Adeera Levin, Masaomi Nangaku, Syed Saad, Marcello Tonelli, Feng Ye, Ikechi G. Okpechi, Aminu K. Bello, David W. Johnson, Jorge Cerda

**Affiliations:** 1 Faculty of Medicine, University of Queensland, Brisbane, Queensland, Australia; 2 Department of Internal Medicine, Nephrology Unit, Kinshasa University Hospital, University of Kinshasa, Kinshasa, Democratic Republic of the Congo; 3 Department of Kidney and Transplant Services, Division of Medicine, Princess Alexandra Hospital, Woolloongabba, Queensland, Australia; 4 Australasian Kidney Trials Network at the University of Queensland, Brisbane, Queensland, Australia; 5 Division of Nephrology, Department of Medicine, University of Alabama at Birmingham, Birmingham, Alabama, United States of America; 6 The International Society of Nephrology, Brussels, Belgium; 7 Population Health Sciences, Bristol Medical School, University of Bristol, Bristol, United Kingdom; 8 George Institute for Global Health, University of New South Wales (UNSW), New Delhi, India; 9 School of Public Health, Imperial College, London, United Kingdom; 10 Manipal Academy of Higher Education, Manipal, India; 11 Division of Nephrology, Department of Medicine, University of British Columbia, Vancouver, British Columbia, Canada; 12 Division of Nephrology and Endocrinology, The University of Tokyo Graduate School of Medicine, Tokyo, Japan; 13 Division of Nephrology and Immunology, Faculty of Medicine and Dentistry, University of Alberta, Edmonton, Alberta, Canada; 14 Department of Medicine, University of Calgary, Calgary, Alberta, Canada; 15 Canada and Pan-American Health Organization/World Health Organization’s Collaborating Centre in Prevention and Control of Chronic Kidney Disease, University of Calgary, Calgary, Alberta, Canada; 16 Division of Nephrology and Hypertension, University of Cape Town, Cape Town, South Africa; 17 Kidney and Hypertension Research Unit, University of Cape Town, Cape Town, South Africa; 18 Centre for Kidney Disease Research, University of Queensland at Princess Alexandra Hospital, Brisbane, Queensland, Australia; 19 Translational Research Institute, Brisbane, Queensland, Australia; 20 Division of Nephrology, Department of Medicine, Albany Medical College, Albany, New York, United States of America; ICMR-National Institute of Epidemiology, INDIA

## Abstract

Acute kidney injury (AKI) is a significant cause of morbidity and mortality, especially in low and lower-middle income countries. Data from the third iteration of the International Society of Nephrology Global Kidney Health Atlas (ISN-GKHA) were used to evaluate the organization of structures and services for the provision of AKI care in world countries and ISN regions. An international survey of key stakeholders (clinicians, policymakers, and patient advocates) from countries affiliated with the ISN was conducted from July to September 2022 to assess structures and services for AKI care across countries. Main findings of the study show that overall, 167 countries or jurisdictions participated in the survey, representing 97.4% of the world’s population. Only 4% of countries had an AKI detection program based on national policy or guideline, and 50% of these countries used a reactive approach for AKI identification (i.e., cases managed as identified through clinical practice). Only 19% of national governments recognized AKI as a healthcare priority. Almost all countries (98% of the countries surveyed) reported capacity to provide acute hemodialysis (HD) for AKI, but in 31% of countries, peritoneal dialysis (PD) was unavailable for AKI. About half of all countries (44% of countries surveyed) provided acute dialysis (HD or PD) via public funding, but funding availability varied across ISN regions, including less than a quarter of countries in Oceania and South East Asia (17%) and Africa (24%) and highest availability in Western Europe (91%). Availability increased with the increasing country income level. Initiatives have been developed to propose and promote optimal care for AKI (including the ISN 0-by-25 initiative), but capacity for optimal AKI care remains low, particularly in low- and lower-middle-income countries. Concerted efforts by the global community are required to close these gaps, to improve AKI outcomes across the world.

## Introduction

By 2013 the global burden of AKI was estimated at 13.3 million cases, with 85% of cases found in low-income countries (LICs) or lower-middle income countries (LMICs) [1]. A similarly timed meta-analysis showed that almost one in five adults and one in three children experienced AKI while hospitalized [2]. While the incidence of AKI is similar or higher in LMICs than in high-income countries (HICs), mortality is higher in LMICs [1, 3].

In LICs where AKI commonly develops in the community in the setting of dehydration, infections, and environmental toxins, the capacity for timely detection and management is severely limited [3]. Over time, AKI can lead to chronic kidney disease (CKD) and other non-kidney complications. The resulting prolongation of hospital stays and the need for kidney replacement therapies (KRT) are major drivers of AKI-associated healthcare costs [4].

Despite the 2015 call to arms from the International Society of Nephrology (ISN) 0by25 initiative to eliminate preventable deaths from AKI by 2025 [3], there remains variable and often insufficient capacity around the world to detect, manage, and prevent AKI. Previous iterations of the ISN Global Kidney Health Atlas (ISN-GKHA) have brought to light the importance of developing sustainable programs for AKI detection as well as disease-specific registries that can inform clinical practice and help advocacy efforts [3, 5, 6]. In this manuscript, we leveraged data from the third iteration of the ISN-GKHA to report on the capacity for detection, monitoring, and management of AKI around the world.

## Methods

### Study design and data sources

Detailed methods of the ISN-GKHA have been published previously [7, 8]. In brief, two strategies were used to obtain country-level data on kidney care capacity. The first component involved an in-depth literature review of published data, grey literature, kidney registries, and databases. The second component involved a multinational, cross-sectional, online survey of opinion leaders using the six World Health Organization (WHO) health system building blocks, available in the supporting information S1 File [9]. Three leaders were identified from each country to participate in the survey, including a nephrology society leader, a policymaker, and a leader of a patient representative organization. All stakeholders answered the same survey questions.

The present study focuses on the assessment of the global capacity for detection, monitoring, and management of AKI. In more detail, this included an evaluation of the: 1) availability and funding structures for acute KRT, 2) availability and nature of health information systems to detect, prevent, and manage AKI, 3) recognition of AKI as a national priority, and 4) capacity for disaster preparedness. These measures were assessed to be "generally available," "generally not available," "never," "unknown," or "not applicable." "Generally available" was defined as being present in at least 50% of the health institutions in the country and "generally not available" if present in less than 50% of the institutions that provided such service at the stated quality.

Key stakeholders in 191 countries with ISN affiliated societies were sent an electronic link to the survey’s online portal via REDCap (www.redcapcloud.com). The survey was conducted from June 1 to September 30, 2022, and was coordinated through the ISN’s 10 regional boards (Africa, Eastern and Central Europe, Latin America, Middle East, North America and the Caribbean, North and East Asia, Oceania and South East Asia (OSEA), Newly Independent States (NIS) and Russia, South Asia, and Western Europe).

### Data handling and analysis

As each country was the unit of analysis, data from multiple respondents within the same country were synthesized into a single response after regional board representatives clarified any inconsistencies. Further validation was carried out at national and regional levels by triangulating the findings with published literature and gray sources of information (i.e., government reports and other sources provided by the survey respondents). Responses were summarized based on the key survey domains using a descriptive statistical approach and reported as counts with percentages or medians with interquartile range (IQR), where appropriate. The results were then stratified by ISN region and World Bank country income group: LICs, LMICs, upper-middle income (UMICs), and HICs (estimated in June 2022). The analysis was conducted using STATA 17 software (Stata Corporation, 2017).

### Ethics approval

The University of Alberta Research Ethics Committee approved this project (protocol number: PRO00063121). Consent was obtained by email from survey respondents. Our study did not report experiments on humans and/or the use of human tissue samples.

## Results

### Characteristics of participating countries

A total of 167 countries responded to the survey (20 LICs, 45 LMICs, 39 UMICs, and 63 HICs), representing 97.4% of the world’s population. Survey respondents were mainly nephrologists (84%), followed by non-nephrologist physicians (5%), non-physician health care professionals (2%), administrators/policymakers (4%), and others affiliated with advocacy groups for people living with kidney disease (5%).

### Availability and funding structures for acute kidney replacement therapies

**Fig 1:**
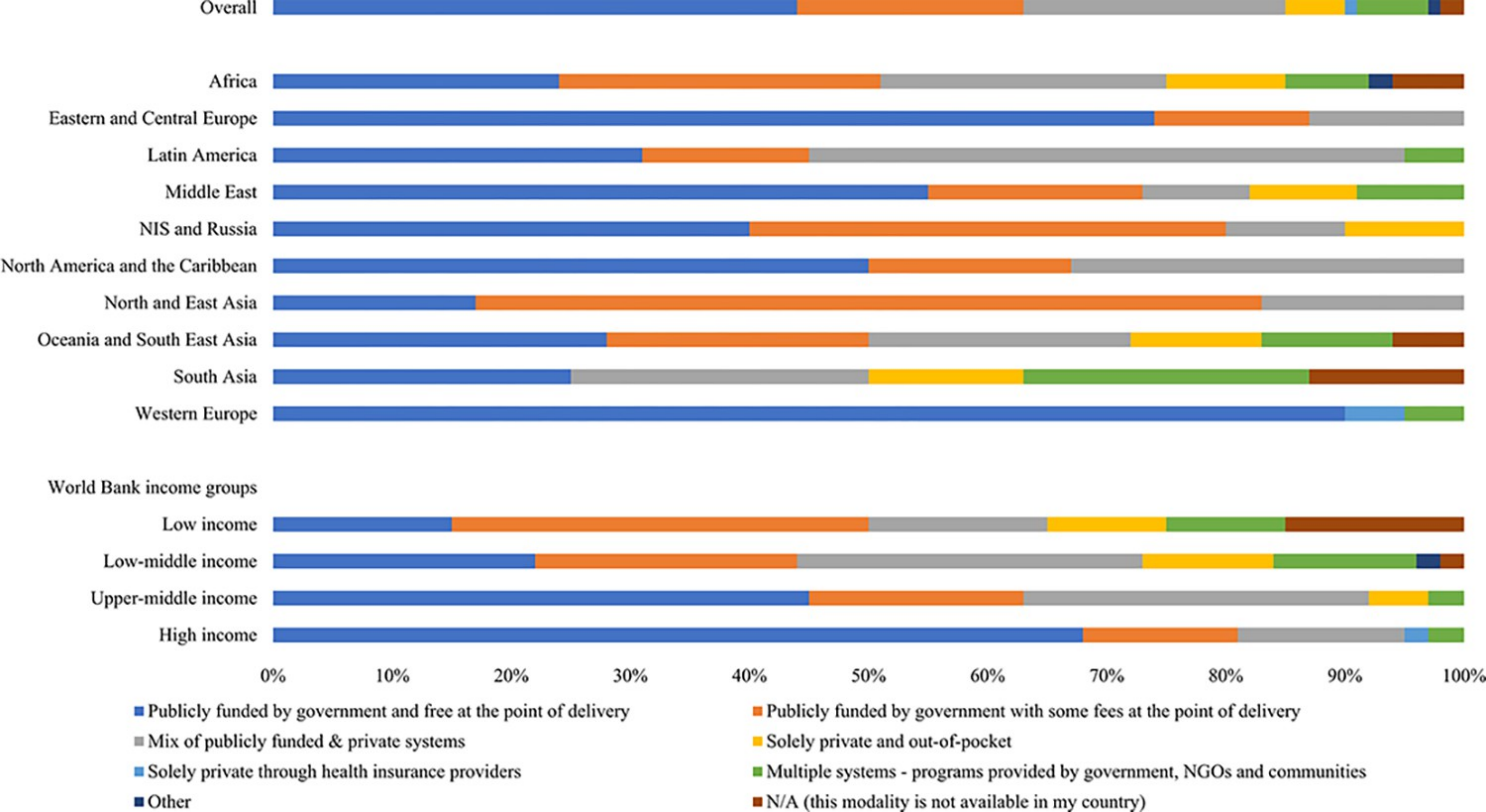
Funding structures for acute KRT (HD or PD), by ISN regions and World Bank income groups*. *Values represent absolute number of countries in each category expressed as a percentage of total number of countries. Abbreviations: KRT = kidney replacement therapy; HD = hemodialysis; PD = peritoneal dialysis; ISN = International Society of Nephrology; NIS = Newly Independent States: NGO = non-governmental organizations.

Approximately half of all countries (44%, 73 of 166) provided acute KRT, hemodialysis (HD) or peritoneal dialysis (PD), via public funding with no fees to the recipient, especially in countries from the ISN Western Europe (n = 20; 91%) and Eastern and Central Europe (n = 12; 75%) regions. In most other countries, acute KRT was funded either by the government with some fees to the recipient or through a mix of public and private systems. As country income level increased, so did the number of countries with freely available and publicly funded structures for acute KRT (Fig 1).

**Fig 2:**
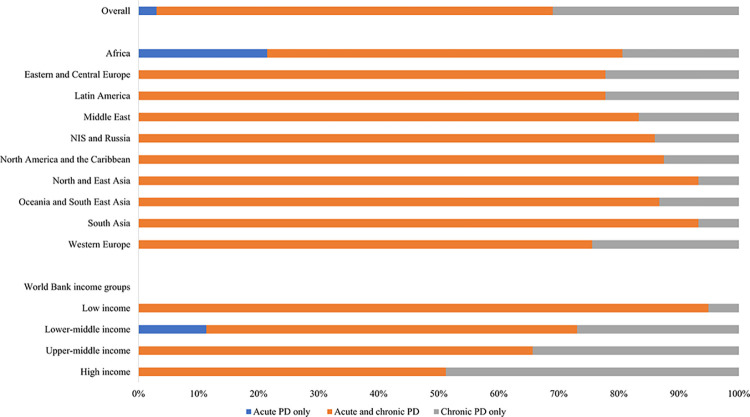
Accessibility of PD services, by ISN regions and World Bank income groups*. *Values represent absolute number of countries in each category expressed as a percentage of total number of countries. Abbreviations: PD = peritoneal dialysis; ISN = International Society of Nephrology; NIS = Newly Independent States.

A majority of countries could provide both acute and chronic PD (n = 86; 66%), while in a third of countries PD was only utilized as a chronic therapy (n = 40; 31%), and in four African countries it was only utilized as an acute therapy (3%) (Fig 2).

### Health information systems for detection and monitoring of AKI

#### Acute kidney injury detection programs

**Fig 3:**
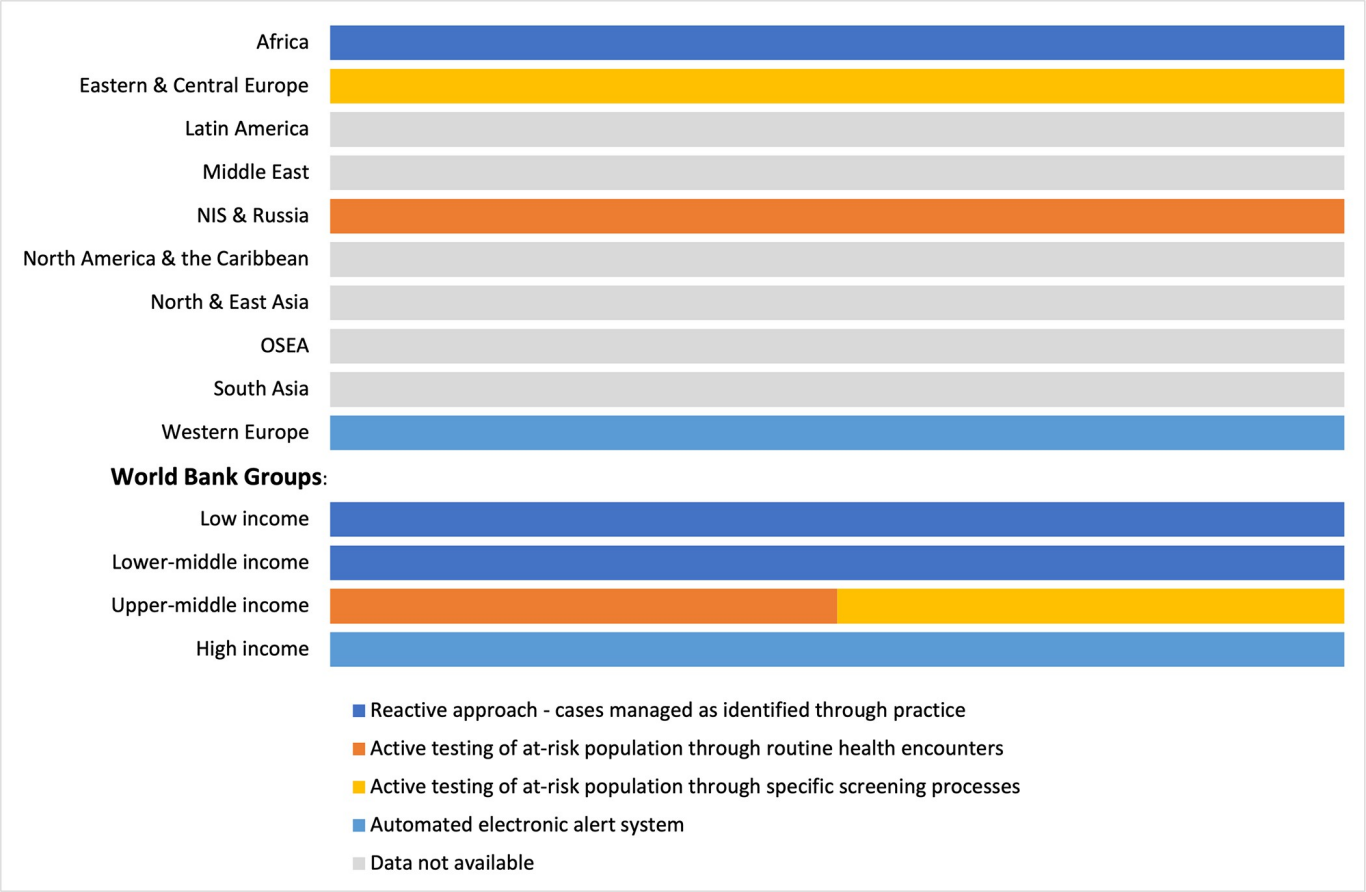
Methods of implementing AKI detection programs, by ISN regions and World Bank income groups*. *Values represent absolute number of countries in each category expressed as a percentage of total number of countries. Abbreviations: AKI = acute kidney injury; ISN = International Society of Nephrology; NIS = Newly Independent States.

In a third of countries (28%, 45 out of 162), specific groups of people were considered to be at increased risk of AKI. This proportion was highest in the ISN North and East Asia (n = 3; 50%), Western Europe (n = 11; 50%), and OSEA (n = 11; 61%) regions and among HICs (n = 25; 40%) (S1 Fig). Detection programs for AKI could have a reactive approach—cases managed as identified through practice–or a proactive approach which involved active screening of at-risk population—either through routine health encounters or specific screening processes—or through automated computation by pathology systems with electronic alerts. Only 6 of 162 (4%) countries had an AKI detection program based on national policy and/or guidelines (S2 Fig). Of these, 3 were from the Africa region and used a reactive approach, while one from NIS and Russia and one from Eastern and Central Europe reported using active testing of at-risk populations through routine health encounters in the former and, through a specific testing process in the latter. One country in the Western Europe region reported using an automated electronic alert system. The reactive approach was especially common in LICs and LMICs (Fig 3).

Most countries (96 of 162, 59%) had no mechanism to ensure the validity and the quality of data in health information systems, particularly among countries in the ISN South Asia (n = 6; 86%), Africa (n = 29; 74%), Middle East (n = 8; 73%), and OSEA (n = 13; 72%) regions (S3 Fig). The lack of data validation and quality assurance processes was common in LICs and LMICs.

#### Acute kidney injury registries

**Fig 4:**
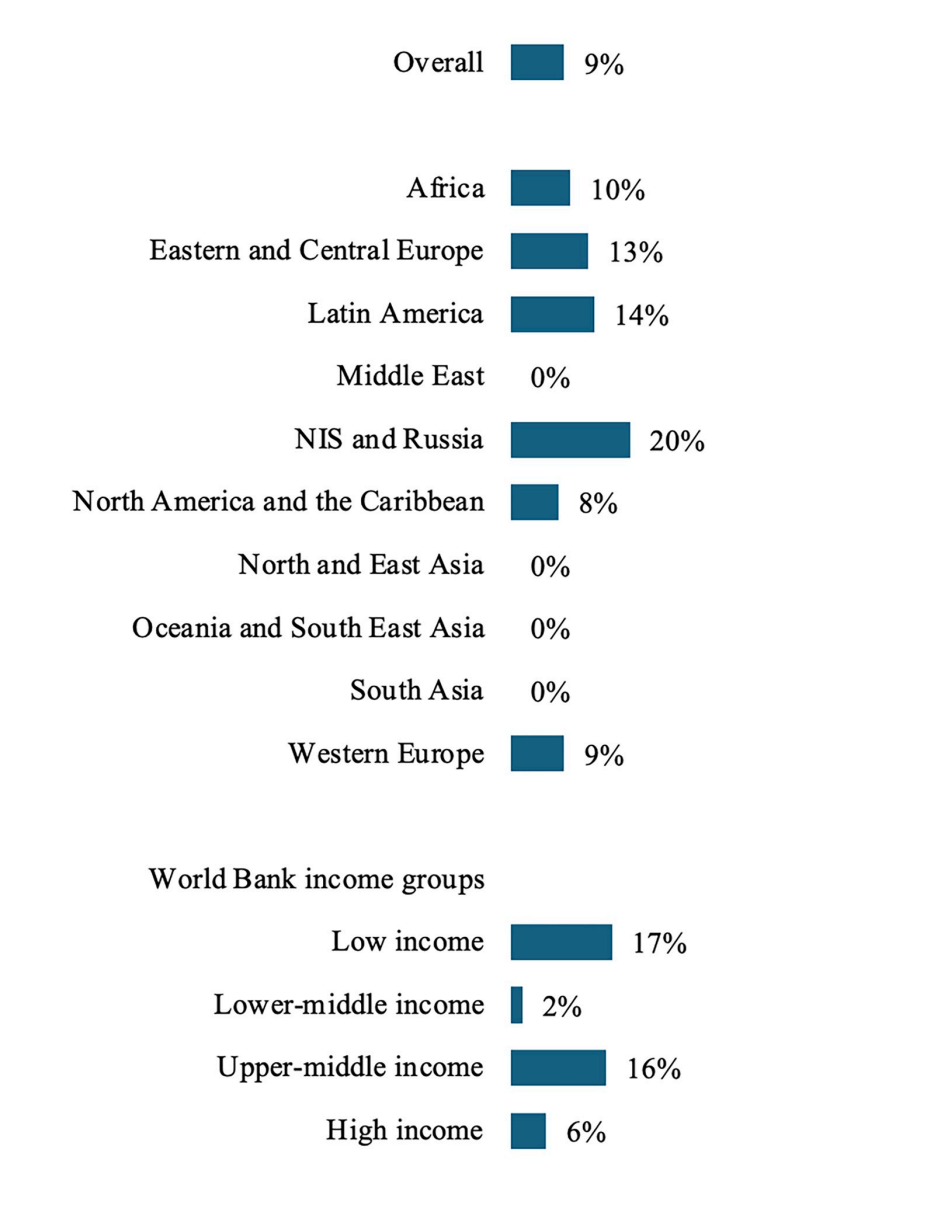
Prevalence of AKI registries, by ISN regions and World Bank income groups*. *Values represent absolute number of countries in each category expressed as a percentage of total number of countries. Abbreviations: AKI = acute kidney injury; ISN = International Society of Nephrology; NIS = Newly Independent States.

Only 14 (9%) countries reported having an AKI registry, of which 10 were in HICs or UMICs and 4 were in the ISN Africa (Fig 4) region. Of the existing registries, participation appeared evenly distributed between those that were voluntary and those that were mandatory (n = 5; 36 vs n = 6; 43%) (S1 Table). Half of the AKI registries (n = 7) were national, mainly in the ISN Latin American region, followed by local/hospital/country registries. Most registries (n = 10; 71%) covered the full spectrum of AKI and a minority recorded only AKI requiring KRT (n = 4; 29%). Information on the incidence of AKI, risk factors, etiology, and patient outcomes was collected in at least 50% of registries, except for outcomes of people with AKI on KRT (n = 6; 43%). Latin America was the ISN region with the highest proportion of registries containing complete information, followed by Eastern and Central Europe.

#### Recognition of AKI as a national health priority

**Fig 5:**
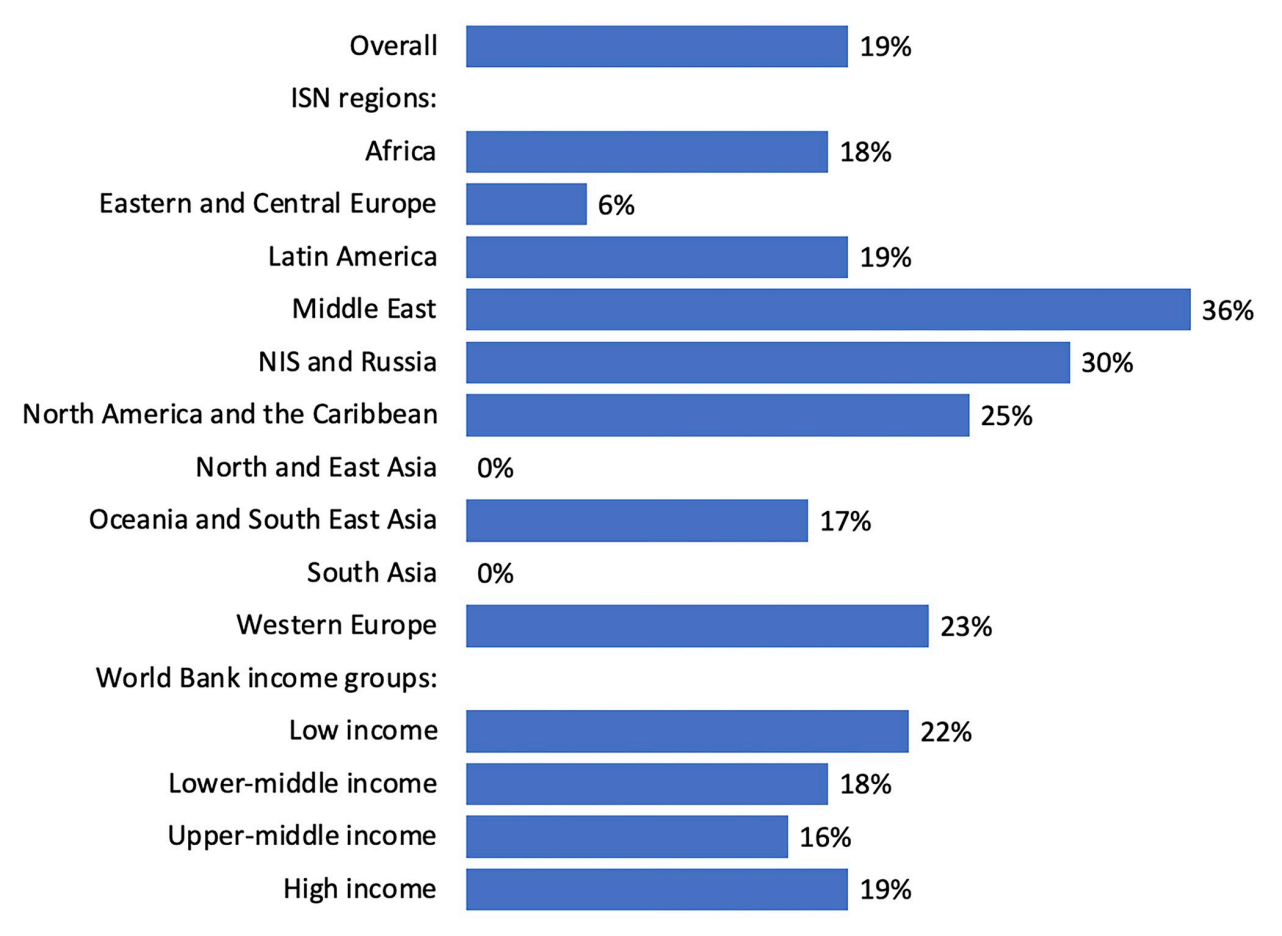
Government recognition of AKI as a health priority and existence of advocacy groups, by ISN regions and World Bank income groups*. *Values represent absolute number of countries in each category expressed as a percentage of total number of countries. Abbreviation: AKI = acute kidney injury; ISN = International Society of Nephrology; NIS = Newly Independent States.

Only 19% of participating countries (30 of 162) reported that their government recognized AKI and/or its treatment and prevention as a health priority (Fig 5). The recognition of AKI as a health priority varied widely across ISN regions and was greatest in the Middle East (n = 4; 36%), NIS and Russia (n = 3; 30%), followed by North America and the Caribbean (n = 3; 25%). Worldwide, the recognition of AKI as a health priority was low across all country income levels: LICs (n = 4; 22%), LMICs (n = 8; 18%), UMICs (n = 6; 16%), and HICs (n = 12; 19%). Similarly, advocacy groups at higher levels of government (e.g., parliamentary committees) or non-government organizations that increase advocacy and raise awareness about AKI were reported in only 11% of countries (n = 18) (Fig 5). Less than a fifth of countries across all income groups reported having an advocacy group with the highest proportion noted in LICs (n = 3; 17%).

## Discussion

This study brings to light several important aspects of AKI care around the world. Detection programs for AKI are sparse and those that exist often use a reactive approach despite clearly identified groups of people at increased risk of AKI. Few countries recognize AKI as a national health priority at the government level and less than 10% of countries around the world have a dedicated AKI registry based on national guidelines. Public funding of acute KRT becomes less common as country income level decreases and almost a third of countries are unable to deliver more cost-effective acute PD to support people with AKI.

While technologies of acute KRT continue to evolve, implementation and adoption of such technologies in LICs and LMICs have been slow and suboptimal. It’s a reality that most acute KRT is delivered in high dependency units around the world. Supporting a person with severe AKI is therefore often subject to the capacity and available resources of these small and costly units that must cater for all forms of organ failure. Increased funding and workforce training to develop quality, sustainable acute PD programs would create a paradigm shift in AKI care, moving it from the critical care units to the general wards and community clinics. While the evidence suggests that funding for acute KRT at a government level generally leads to more robust and sustainable KRT programs [10], our findings indicate that recognition of AKI as a national health priority is still far from a reality in most countries. Partnerships, such as those created through the ISN’s Sister Renal Center program that facilitate pairing of units with variable levels of familiarity with acute KRT programs, have led to extraordinary opportunities for capacity building among LICs [11]. At a more grassroots level, the ISN’s Saving Young Lives program has for many years been identifying champions of acute PD in the developing world to equip them with the knowledge and skills to manage AKI under strain and with limited resources [12].

The target of 0 preventable deaths by 2025 was largely based on the premise that most of the morbidity and mortality associated with AKI in the developing world is avoidable if it were met with timely detection and appropriate treatment [3]. However, to detect anything early, one must first know where to look. Our finding that less than a third of countries could identify specific groups at risk of AKI, speaks to a significant level of under-reporting and characterization of AKI, particularly among people from LICs and LMICs. The lack of recognition of AKI as a national health priority probably reflects a gap in the awareness of AKI as a common and often preventable and treatable disease. This also explains, at least partially, our finding of a very small number of countries with established AKI detection programs, and even a smaller number with programs that are pre-emptive, and risk based. In LICs, this may be further complicated by limited access to diagnostic and therapeutic interventions and the fact that most AKI occurs in the community setting. Nevertheless, in 2020, Macedo et al. demonstrated the success of an AKI detection and management program in community clinics of Bolivia, Nepal, and Malawi using a simple symptom-based score, point of care creatinine and urine dipstick, to determine AKI risk and baseline level of chronicity [6]. Incorporating AKI into a national health policy requires a multipronged approach with effective collaboration between international organizations like ISN and the WHO, government agencies, healthcare providers, researchers, and the community to raise awareness of the importance of AKI and its prevention/treatment. Strategies include provision of population-based education, targeted screening programs of at-risk populations and enhanced detection through electronic medical record alert systems, quality registries, and ongoing research to advance knowledge and improve outcomes [13]. The ISN-WHO AKI Tool kit launched in February 2024, is an example of a successful collaboration plan to highlight the importance of prevention and early recognition of AKI [14].

Detection and management programs for AKI must be informed by country and region- specific data. This can only be achieved through the development of disease registries that are based on national guidelines and which collect standardized data in a systematic and validated fashion. However, and contrary to the situation for kidney failure, few countries have an AKI registry, and those that do are usually subnational or collect limited information. Kidney registries are especially absent in LICs and LMICs due to a spectrum of political, financial, and cultural barriers. To overcome these, regional and international collaborations, such as those promoted by the Sharing Expertise to Support the set-up of Renal Registries (SharE-RR) project, an ISN initiative to help develop kidney registries, are critical to developing registries in LICs and improving their quality and sustainability [15]. Kidney disease registries are key in health infrastructure planning, benchmarking, continuous quality improvement, hypothesis generation, and real-world trials [16].

The strengths of this study include the use of a validated framework based on the WHO’s key building blocks of a functional health system. Responses were obtained from a representative number of key stakeholders with comprehensive coverage of the world population across all ISN regions and income levels, thereby ensuring generalizability. Data accuracy was enhanced by gathering results through multiple resources and a rigorous verification process. Despite these strengths, there is a residual risk of data inaccuracy, as well as response bias, particularly with regards to social desirability. While understanding workforce capacity specifically for AKI would have been useful, this could not be ascertained in an isolated manner through the survey or literature review. The granularity of information was limited to reduce the risk of survey fatigue and incomplete survey responses.

Almost a decade after the publication of the 0by25 landmark paper which characterized AKI as a ‘human rights case for nephrology’, its recognition, detection, and management, especially among LICs and LMICs, continues to be neglected. We must encourage initiatives and collaborations that promote the use of acute PD, which can be lifesaving and easily implemented now. Importantly, we must raise awareness of AKI as a common, preventable, and treatable disease through advocacy campaigns and continued publication of quality research that delineates the burden of AKI and tracks our efforts to curve it.

## Supporting information

S1 Checklist Inclusivity in global research. (DOCX)

S1 File International Society of Nephrology Global Kidney Health Atlas (ISN-GKHA) questionnaire. (PDF)

S1 Fig Identification of specific groups considered to be at increased risk of AKI, by ISN regions and World Bank income groups. (PDF)

S2 Fig Countries with AKI detection programs based on national policy or guidelines, by ISN regions and World Bank income groups. (PDF)

S3 Fig Prevalence of mechanisms to ensure validity and quality of data in health information systems, by ISN regions and World Bank income groups. (PDF)

S4 Fig Countries with guidelines for disaster preparedness, by ISN regions and World Bank income groups. (PDF)

S5 Fig Countries with a representative in the renal disaster task force, by ISN regions and World Bank income groups. (PDF)

S1 Table Characteristics of existing AKI registries, by ISN regions and World Bank income groups (N, %). (DOCX)

S1 Data AKI. (XLSX)
