# Supplementary material for: A global snapshot on health systems capacity for detection, monitoring, and management of acute kidney injury: A multinational study from the ISN-GKHA
Source: PLOS Glob Public Health. 2024 Oct 15;4(10):e0003823. doi: 10.1371/journal.pgph.0003823 (PMC11478907; doi:10.1371/journal.pgph.0003823)
Supplement: S1 File — (PDF) [file pgph.0003823.s002.pdf]

ISN-Global Kidney Health Atlas (ISN-GKHA) Survey - Language

ISN-Global Kidney Health Atlas (ISN-GKHA) Survey

Please complete the survey below.

Thank you!

Please select your language / Veuillez sélectionner votre langue / Por favor, seleccione su idioma

○ English
○ Français
○ Español

ISN-Global Kidney Health Atlas (ISN-GKHA) Survey

Topical survey:

Assessment of Global Kidney Health Care Status: Capacity, Availability, Accessibility, Affordability and Outcomes

Country Profiles of Assessment of Global Kidney Health Care Status: Capacity, Availability, Accessibility, Affordability and Outcomes

The International Society of Nephrology (ISN) has been working collaboratively with existing organizations and initiatives at international and national levels - to promote early detection and effective treatment of kidney diseases in order to improve patient health and quality of life. Through understanding and potentially helping to shape relevant health policies, practices, and infrastructure, ISN aims to facilitate the implementation of equitable and ethical care for kidney patients in all regions and countries of the world.

The ISN has conducted a research exercise on the status of care for kidney patients across all countries of the world published in its Global Kidney Health Atlas with 1st iteration (2017; https://www.theisn.org/wp-content/uploads/2021/05/GKDAtlas_2017_FinalVersion-1.pdf) and 2nd iteration (2019; https://www.theisn.org/wp-content/uploads/2021/05/GKHAtlas_2019_WebFIle-1.pdf).

The ISN-GKHA demonstrated significant inter- and intra-regional variability in global kidney care, with significant gaps in kidney health workforce, health service delivery, essential medicines and technologies, health financing, leadership and governance, health information systems, strategies and policy frameworks, and research capacity and development, particularly in low- and middle-income countries. This has provided a platform for championing the cause of chronic kidney disease (CKD) using the identified gaps in Universal Healthcare domains and has provided a foundation for a global CKD surveillance and benchmarking network.

This third iteration of the survey by the ISN is to understand, compare and monitor how different countries around the world detect, treat, monitor, and advocate for people with kidney disease with a key focus on capacity, availability, accessibility, affordability, and outcomes.

It will determine the capacity and readiness of nations towards achieving universal access to equitable integrated kidney care (including kidney replacement therapy and conservative care). This iteration also includes a patient questionnaire which provides patient perspective on access to, and quality of care being delivered.

This questionnaire is designed to address the core areas which inform aspects of universal health coverage specific to integrated kidney care: health financing, workforce, essential medications and health products access, health information systems and statistics, policies, and service delivery and safety as well as the response of the nephrology community and

capacity for research and development in kidney care. Using this framework, we will be able to develop an appropriate global perspective on the state of access to, and quality of kidney care globally. Obtaining universal, complete, and accurate responses is critical to closing the gaps that exist in kidney care globally.

If you have any questions about completing the questionnaire, please contact: Sandrine Damster (email: GlobalAtlas@theisn.org).

Thank you for your involvement and readiness to participate.

Professor Agnes Fogo

President, International Society of Nephrology (ISN)

List of abbreviations:

AKI: Acute kidney injury
AV fistula: Arteriovenous fistula
APD: Automated peritoneal dialysis
BP: Blood pressure
CKD: Chronic kidney disease
CKM: Conservative kidney management
ESKD: End-stage kidney disease
KRT: Kidney replacement therapy (i.e., hemodialysis, peritoneal dialysis, kidney transplantation)
NCD: Non-communicable disease
NGO: Non-governmental organization
HD: Hemodialysis
PD: Peritoneal dialysis
PTH: Parathyroid hormone

1. Survey ID (provided in email): ______________________

2. Current position: ______________________

3. Your role	◯ Nephrologist<br>◯ Pediatric nephrologist<br>◯ Non-nephrologist (physician)<br>◯ Health professional (non-physician)<br>◯ Administrator/policymaker/civil servant<br>◯ Other
3. If other, please specify	__________________________________

4. In which country do you reside?

◯ Afghanistan
◯ Åland Islands
◯ Albania
◯ Algeria
◯ American Samoa
◯ Andorra
◯ Angola
◯ Anguilla
◯ Antarctica
◯ Antigua And Barbuda
◯ Argentina
◯ Armenia
◯ Aruba
◯ Australia
◯ Austria
◯ Azerbaijan
◯ Bahamas
◯ Bahrain
◯ Bangladesh
◯ Barbados
◯ Belarus
◯ Belgium
◯ Belize
◯ Benin
◯ Bermuda
◯ Bhutan
◯ Bolivia
◯ Bosnia And Herzegovina
◯ Botswana
◯ Bouvet Island
◯ Brazil
◯ British Indian Ocean Territory
◯ Brunei Darussalam
◯ Bulgaria
◯ Burkina Faso
◯ Burundi
◯ Cambodia
◯ Cameroon
◯ Canada
◯ Cape Verde
◯ Cayman Islands
◯ Central African Republic
◯ Chad
◯ Chile
◯ China
◯ Christmas Island
◯ Cocos (Keeling) Islands
◯ Colombia
◯ Comoros
◯ Congo
◯ Congo (The Democratic Republic Of The)
◯ Cook Islands
◯ Costa Rica
◯ Cote D'ivoire
◯ Croatia
◯ Cuba
◯ Cyprus
◯ Czechia
◯ Denmark
◯ Djibouti
◯ Dominica
◯ Dominican Republic
◯ Ecuador
◯ Egypt
◯ El Salvador
◯ Equatorial Guinea
◯ Eritrea
◯ Estonia
◯ Ethiopia

◯ Falkland Islands (Malvinas)
◯ Faroe Islands
◯ Fiji
◯ Finland
◯ France
◯ French Guiana
◯ French Polynesia
◯ French Southern Territories
◯ Gabon
◯ Gambia
◯ Georgia
◯ Germany
◯ Ghana
◯ Gibraltar
◯ Greece
◯ Greenland
◯ Grenada
◯ Guadeloupe
◯ Guam
◯ Guatemala
◯ Guernsey
◯ Guinea
◯ Guinea-bissau
◯ Guyana
◯ Haiti
◯ Heard Island And Mcdonald Islands
◯ Holy See (Vatican City State)
◯ Honduras
◯ Hong Kong
◯ Hungary
◯ Iceland
◯ India
◯ Indonesia
◯ Iran, Islamic Republic Of
◯ Iraq
◯ Ireland
◯ Isle Of Man
◯ Israel
◯ Italy
◯ Jamaica
◯ Japan
◯ Jersey
◯ Jordan
◯ Kazakhstan
◯ Kenya
◯ Kiribati
◯ Korea: Democratic People's Republic Of
◯ Korea: Republic Of
◯ Kosovo
◯ Kuwait
◯ Kyrgyzstan
◯ Lao People's Democratic Republic
◯ Latvia
◯ Lebanon
◯ Lesotho
◯ Liberia
◯ Libyan Arab Jamahiriya
◯ Liechtenstein
◯ Lithuania
◯ Luxembourg
◯ Macao
◯ Macedonia
◯ Madagascar
◯ Malawi
◯ Malaysia
◯ Maldives
◯ Mali
◯ Malta
◯ Marshall Islands
◯ Martinique
◯ Mauritania

◯ Mauritius
◯ Mayotte
◯ Mexico
◯ Micronesia
◯ Moldova, Republic Of
◯ Monaco
◯ Mongolia
◯ Montenegro
◯ Montserrat
◯ Morocco
◯ Mozambique
◯ Myanmar
◯ Namibia
◯ Nauru
◯ Nepal
◯ Netherlands
◯ Netherlands Antilles
◯ New Caledonia
◯ New Zealand
◯ Nicaragua
◯ Niger
◯ Nigeria
◯ Niue
◯ Norfolk Island
◯ Northern Mariana Islands
◯ Norway
◯ Oman
◯ Pakistan
◯ Palau
◯ Palestinian Territory, Occupied
◯ Panama
◯ Papua New Guinea
◯ Paraguay
◯ Peru
◯ Philippines
◯ Pitcairn
◯ Poland
◯ Portugal
◯ Puerto Rico
◯ Qatar
◯ Reunion
◯ Romania
◯ Russian Federation
◯ Rwanda
◯ Saint Helena
◯ Saint Kitts And Nevis
◯ Saint Lucia
◯ Saint Pierre And Miquelon
◯ Saint Vincent And The Grenadines
◯ Samoa
◯ San Marino
◯ Sao Tome And Principe
◯ Saudi Arabia
◯ Senegal
◯ Serbia
◯ Seychelles
◯ Sierra Leone
◯ Singapore
◯ Slovakia
◯ Slovenia
◯ Solomon Islands
◯ Somalia
◯ South Africa
◯ South Georgia And The South Sandwich Islands
◯ South Sudan
◯ Spain
◯ Sri Lanka
◯ Sudan
◯ Suriname
◯ Svalbard And Jan Mayen
◯ Swaziland

◯ Sweden
◯ Switzerland
◯ Syrian Arab Republic
◯ Taiwan
◯ Tajikistan
◯ Tanzania, United Republic Of
◯ Thailand
◯ Timor-leste
◯ Togo
◯ Tokelau
◯ Tonga
◯ Trinidad And Tobago
◯ Tunisia
◯ Turkey
◯ Turkmenistan
◯ Turks And Caicos Islands
◯ Tuvalu
◯ Uganda
◯ Ukraine
◯ United Arab Emirates
◯ United Kingdom
◯ United States
◯ United States Minor Outlying Islands
◯ Uruguay
◯ Uzbekistan
◯ Vanuatu
◯ Venezuela
◯ Viet Nam
◯ Virgin Islands, British
◯ Virgin Islands, U.S.
◯ Wallis And Futuna
◯ Western Sahara
◯ Yemen
◯ Zambia
◯ Zimbabwe

---

5. In which city do you reside?

____

A. Health finance and service delivery

A1. Healthcare system and funding mechanism

A1.1. In general, what best describes your healthcare system funding structure for non-dialysis Chronic Kidney Disease (CKD)? (please choose the most appropriate response)	◯ Publicly funded by government and free at the point of delivery<br>◯ Publicly funded by government but with some fees at the point of delivery<br>◯ A mix of publicly funded (whether or not publicly funded component is free at point of delivery) and private systems (please explain)<br>◯ Solely private and out-of-pocket<br>◯ Solely private through health insurance providers<br>◯ Multiple systems - programs provided by government, NGOs, and communities<br>◯ Other (please specify)
A1.1. If other, please specify	__________________________________
A1.1. If a mix of publicly and and privately funded, please explain	__________________________________

A1.2. In general, what best describes your healthcare system funding structure for KRT (kidney replacement therapy)? (please choose the most appropriate response)

Reduce font size if the text below is overlapping.

	Publicly funded by government and free at the point of delivery	Publicly funded by government but with some fees at the point of delivery	A mix of publicly funded (whether or not publicly funded component is free at point of delivery) and private systems (please explain)	Solely private and out-of-pocket	Solely private through health insurance providers	Multiple systems - programs provided by government, NGOs, and communities	Other (please specify)	N/A (this modality is not available in my country)
A1.2.1. Acute dialysis for AKI (hemodialysis or peritoneal dialysis)	○	○	○	○	○	○	○	○

A1.2. In general, what best describes your healthcare system funding structure for KRT (kidney replacement therapy)? (please choose the most appropriate response)

Reduce font size if the text below is overlapping.

A1.2.2. Chronic Hemodialysis	○	○	○	○	○	○	○	○

A1.2. In general, what best describes your healthcare system funding structure for KRT (kidney replacement therapy)? (please choose the most appropriate response)

Reduce font size if the text below is overlapping.

A1.2.3. Chronic Peritoneal dialysis	○	○	○	○	○	○	○	○

A1.2. In general, what best describes your healthcare system funding structure for KRT (kidney replacement therapy)? (please choose the most appropriate response)

Reduce font size if the text below is overlapping.

A1.2.4. Medications for kidney transplantation ○ ○ ○ ○ ○ ○ ○ ○

A1.2.1. If other, please specify

____

A1.2.1. If a mix of publicly and and privately funded, please explain

____

A1.2.2. If other, please specify

____

A1.2.2. If a mix of publicly and and privately funded, please explain

____

A1.2.3. If other, please specify

____

A1.2.3. If a mix of publicly and and privately funded, please explain

____

A1.2.4. If other, please specify

____

A1.2.4. If a mix of publicly and and privately funded, please explain

____

A1.3.1. If KRT is publicly funded (in whole or in part), is this coverage universal (that is, are all residents of your country eligible to participate)?

- ○ Yes, all vulnerable populations are included in the coverage
- ○ No, not all vulnerable populations are included (please provide details)

A1.3.1. If no, please provide details.

____

A1.3.2. If KRT is publicly funded (in whole or in part), are vulnerable populations (refugees, displaced populations) eligible to participate?

- ○ Yes, all vulnerable populations are included in the coverage
- ○ No, not all vulnerable populations are included (please provide details)

A1.3.2. If no, please provide details.

____

A1.3.3. If KRT is publicly funded (in whole or in part), which aspects of care are not included in the coverage? Please check all that apply.	☐ Dialysis<br>☐ Transplantation<br>☐ Comprehensive conservative care (kidney palliative supportive services)<br>☐ Management of associated complications (anaemia, bone disease, malnutrition)<br>☐ None - all aspects funded<br>☐ Other (please specify)
A1.3.3. If other, please specify	__________________________________

A1.4. What best describes your healthcare system's coverage for surgical services for KRT? (please choose the most appropriate response for each row) (skip this section if KRT is unavailable in your country)

Reduce font size if the text below is overlapping.

	Publicly funded by government and free at the point of delivery	Publicly funded by government but with some fees at the point of delivery	A mix of publicly funded (whether or not publicly funded component is free at point of delivery) and private systems (please explain)	Solely private and out-of-pocket	Solely private through health insurance providers	Multiple systems - programs provided by government, NGOs, and communities	Other (please specify)
A1.4.1. Vascular access for hemodialysis (central venous catheters)	○	○	○	○	○	○	○

A1.4. What best describes your healthcare system's coverage for surgical services for KRT? (please choose the most appropriate response for each row) (skip this section if KRT is unavailable in your country)

Reduce font size if the text below is overlapping.

A1.4.2. Vascular access for hemodialysis (fistula or graft creation)	○	○	○	○	○	○	○

A1.4. What best describes your healthcare system's coverage for surgical services for KRT? (please choose the most appropriate response for each row) (skip this section if KRT is unavailable in your country)

Reduce font size if the text below is overlapping.

A1.4.3. Access surgery for peritoneal dialysis (PD catheter insertion)	○	○	○	○	○	○	○

A1.4. What best describes your healthcare system's coverage for surgical services for KRT? (please choose the most appropriate response for each row) (skip this section if KRT is unavailable in your country)

Reduce font size if the text below is overlapping.

A1.4.4. Surgery for kidney transplantation	○	○	○	○	○	○	○

A1.4.1. If other (please specify). __________________________________

A1.4.1. If a mix of public and private funding, please explain. __________________________________

A1.4.2. If other (please specify). __________________________________

A1.4.2. If a mix of public and private funding, please explain. __________________________________

A1.4.3. If other (please specify). __________________________________

A1.4.3. If a mix of public and private funding, please explain. __________________________________

A1.4.4. If other (please specify). __________________________________

A1.4.4. If a mix of public and private funding, please explain. __________________________________

A2. Within-country variation

We are interested in understanding within-country variation in kidney failure (or end-stage kidney disease [ESKD]) care delivery as well as between-country variation

A2.1.1. Does the organization or delivery of kidney failure (ESKD) (kidney failure) care differ regionally within your country?

- ◯ Yes (if possible, please provide brief details)
- ◯ No
- ◯ Unknown

A2.1.1. If yes, please provide details

____

A2.1.2. Does cost of kidney failure (ESKD) (kidney failure) care differ regionally within your country?

- ◯ Yes (if possible, please provide brief details)
- ◯ No
- ◯ Unknown

A2.1.2. If yes, please provide details

____

A2.1.3. Does organization or delivery of kidney failure (ESKD) care differ between children and adults in your country?

- ◯ Yes (if possible, please provide brief details)
- ◯ No
- ◯ Unknown

A2.1.3. If yes, please provide details

____

A2.1.4. Does the access to KRT differ between children and adults in your country?

- ◯ Yes (if possible, please provide brief details)
- ◯ No
- ◯ Unknown

A2.1.4. If yes, please provide details

____

A2.2.1 If KRT services are not equal between adults and children, what is the difference in access to hemodialysis?

- ◯ More HD access for adults than for children
- ◯ More HD access for children than for adults
- ◯ HD access available for adults, unavailable for children
- ◯ HD access available for children, unavailable for adults

A2.2.2 If KRT services are not equal between adults and children, what is the difference in access to peritoneal dialysis?

- ◯ More PD access for adults than for children
- ◯ More PD access for children than for adults
- ◯ PD access available for adults, unavailable for children
- ◯ PD access available for children, unavailable for adults

A2.2.3 If KRT services are not equal between adults and children, what is the difference in access to kidney transplant?

- ◯ More KT access for adults than for children
- ◯ More KT access for children than for adults
- ◯ KT access available for adults, unavailable for children
- ◯ KT access available for children, unavailable for adults

A3. Oversight

A3.1. What best describes the management/oversight of kidney care in your country? Please check all that apply.

- ☐ Managed/overseen by a national body
- ☐ Managed/overseen by provincial/regional/state level authorities only
- ☐ Managed by individual hospitals/trusts/organizations
- ☐ Managed by NGOs
- ☐ Other (please specify)
- ☐ No organized system

A3.1. If other, please specify.

____

A3.2. How would you rate the health infrastructure in your country, in terms of adequacy for providing kidney failure (ESKD) care?

- ◯ Extremely poor
- ◯ Poor/below average
- ◯ Fair/Average
- ◯ Good/above average
- ◯ Excellent

B. Health workforce for nephrology care

B1. Clinical responsibility

B1.1. Who bears primary clinical responsibility for the delivery of kidney failure (ESKD) care in your country?

- ◯ Nephrologists
- ◯ Primary care physicians
- ◯ Nurse practitioners
- ◯ Specialized nurses
- ◯ Multidisciplinary teams
- ◯ Health officers/extension workers
- ◯ Other specialists (please specify)

B1.1. If other, please specify.

____

B2.1.1. Approximately how many nephrologists are there in your country? Please leave blank if unknown.
Nephrologists {b2_1_1_neph}
Adult nephrologists {b2_1_1_ad_neph}
Pediatric nephrologists {b2_1_1_paed_neph}

B2.1.2. What is the percentage of female nephrologists (adults and pediatrics combined) in your country? Please leave blank if unknown.

____

B2.2.1. Approximately how many nephrologist trainees are there in your country? Please leave blank if unknown.

____

B2.2.2. Does a training program for adult nephrologists exist in your country?

- ◯ Yes
- ◯ No
- ◯ Not sure

B2.2.3. If yes to question B2.2.2 above, what is the length of the training program?

- ◯ < 1 year
- ◯ 1 - 2 years
- ◯ 2 - 4 years
- ◯ >4 years

B2.2.4. Does a training program for pediatric nephrologists exist in your country?

- ◯ Yes
- ◯ No
- ◯ Not sure

B2.2.5. If yes to question B2.2.4 above, what is the length of the training program?

- ◯ < 1 year
- ◯ 1 - 2 years
- ◯ 2 - 4 years
- ◯ >4 years

B2.2.6. Is the training program for nephrologists (adults or pediatrics) linked with a research component (e.g., PhD, M.Sc., MPhil, MMed, etc)?

- ◯ Yes
- ◯ No
- ◯ Not sure

B2.3. In your opinion, is there a shortage of any of the following providers in your country for kidney care? Please check all that apply.

- ☐ Nephrologists
- ☐ Pediatric nephrologists
- ☐ Transplant surgeons
- ☐ Surgeons or Interventional radiologists (who can put in arteriovenous hemodialysis access)
- ☐ Surgeons or Interventional radiologists (who can put in peritoneal dialysis access)
- ☐ Dietitians
- ☐ Laboratory technicians
- ☐ Radiologists to conduct and interpret kidney ultrasounds
- ☐ Vascular access coordinators
- ☐ Counsellors/psychologists
- ☐ Transplant coordinators
- ☐ Dialysis nurses
- ☐ Renal nurses
- ☐ Dialysis technicians
- ☐ Social workers
- ☐ Palliative care physicians
- ☐ Kidney supportive care nurses
- ☐ No shortage of any of the staff mentioned above

C. Essential medications and health product access for kidney care
C1. Capacity for KRT service provision

C1.1. Is in-centre hemodialysis (adult and pediatric) available in your country?
- ◯ Yes
- ◯ No

C1.1.1. If yes, how many centres in your country provide chronic hemodialysis (HD)?
____

C1.1.2. Is home hemodialysis (adult and pediatric) available in your country?
- ◯ Yes
- ◯ No

C1.2. Is peritoneal dialysis (PD) (adult and pediatric) available in your country?
- ◯ Yes
- ◯ No

C1.2.1 If yes, how many centres in your country provide chronic PD?
____

C1.2.2. If PD is available, under what circumstances can it be accessed in your country? (Select one)
- ◯ Acute PD only
- ◯ Acute and chronic PD
- ◯ Chronic PD only

C1.2.3. Is automated peritoneal dialysis (APD) (adult and pediatric) available in your country?
- ◯ Yes
- ◯ No

C1.3. Is adult kidney transplantation performed in your country?
- ◯ Yes
- ◯ No

C1.4. Is pediatric (age < 18 years) kidney transplantation performed in your country?
- ◯ Yes
- ◯ No

C1.5.1. If yes, what is the source of donated kidneys? (Please choose the most appropriate response)
- ◯ Deceased donors only
- ◯ Live donors only
- ◯ A combination of deceased and live donors

C1.5.2. If kidneys for transplant come from both deceased and live donors, what percentage are live?
____

C1.5.3. If kidney transplantation is available in your country, what kind of kidney transplant waitlist or waitlists are there?
- ◯ National
- ◯ Regional only
- ◯ None

C1.5.4. If kidney transplantation is available in your country, how many centres perform kidney transplantation?
____

C2. Essential medications and health products access

C2.1. Essential medications and technologies for Kidney Replacement Therapy - Accessibility, affordability and reimbursement plans and quality
(choose the most appropriate response for each question)

Reduce font size if the text below is overlapping.

	Publicly funded by government and free at the point of delivery	Publicly funded by government but with some fees at the point of delivery	A mix of publicly funded (whether or not publicly funded component is free at point of delivery) and private systems (please explain)	Solely private and out-of-pocket	Solely private through health insurance providers	Multiple systems - programs provided by government, NGOs, and communities	Other (please specify)
C2.1.1. For all CKD patients (not on dialysis): How are medications funded?	○	○	○	○	○	○	○

C2. Essential medications and health products access

C2.1. Essential medications and technologies for Kidney Replacement Therapy - Accessibility, affordability and reimbursement plans and quality
(choose the most appropriate response for each question)

Reduce font size if the text below is overlapping.

C2.1.2. For all dialysis patients: How are medications funded? ○ ○ ○ ○ ○ ○ ○

C2. Essential medications and health products access

C2.1. Essential medications and technologies for Kidney Replacement Therapy - Accessibility, affordability and reimbursement plans and quality
(choose the most appropriate response for each question)

Reduce font size if the text below is overlapping.

C2.1.3. For all transplant patients: How are medications funded? ○ ○ ○ ○ ○ ○ ○

C2.1.1. If other, please specify. __________________________________

C2.1.1. If a mix of public and private funding, please explain. __________________________________

C2.1.2. If other, please specify. __________________________________

C2.1.2. If a mix of public and private funding, please explain. __________________________________

C2.1.3. If other, please specify. __________________________________

C2.1.3. If a mix of public and private funding, please explain. __________________________________

C3. Preparation for KRT
Optimal kidney failure (ESKD) care: In the context of the ISN Vision, Mission and Values, we believe all patients approaching kidney failure (ESKD) should receive timely preparation for KRT, so the complications and progression of their disease are minimized, and their choice of clinically appropriate treatment options is optimized. The answers to the following questions are important to improve our understanding of current service provision.

C3.1. Management of hemoglobin level

Please indicate the availability of the following services (tests and treatments) for kidney failure (ESKD) care in your country.
'Generally available' means in 50% or more centres (hospitals or clinics) and 'Generally not available' means: in less than 50% of centres (hospitals or clinics)

	Generally available	Generally not available	Never	Unknown
C3.1.1. Measurement of serum hemoglobin	○	○	○	○

C3. Preparation for KRT

Optimal kidney failure (ESKD) care: In the context of the ISN Vision, Mission and Values, we believe all patients approaching kidney failure (ESKD) should receive timely preparation for KRT, so the complications and progression of their disease are minimized, and their choice of clinically appropriate treatment options is optimized. The answers to the following questions are important to improve our understanding of current service provision.

C3.1. Management of hemoglobin level

Please indicate the availability of the following services (tests and treatments) for kidney failure (ESKD) care in your country.
'Generally available' means in 50% or more centres (hospitals or clinics) and 'Generally not available' means: in less than 50% of centres (hospitals or clinics)

C3.1.2. Measurement of iron parameters (iron, ferritin, transferrin saturation)	○	○	○	○

C3. Preparation for KRT
Optimal kidney failure (ESKD) care: In the context of the ISN Vision, Mission and Values, we believe all patients approaching kidney failure (ESKD) should receive timely preparation for KRT, so the complications and progression of their disease are minimized, and their choice of clinically appropriate treatment options is optimized. The answers to the following questions are important to improve our understanding of current service provision.

C3.1. Management of hemoglobin level

Please indicate the availability of the following services (tests and treatments) for kidney failure (ESKD) care in your country.
'Generally available' means in 50% or more centres (hospitals or clinics) and 'Generally not available' means: in less than 50% of centres (hospitals or clinics)

C3.1.3. Measurement of inflammatory markers (for example, serum C-reactive protein)	○	○	○	○

C3. Preparation for KRT
Optimal kidney failure (ESKD) care: In the context of the ISN Vision, Mission and Values, we believe all patients approaching kidney failure (ESKD) should receive timely preparation for KRT, so the complications and progression of their disease are minimized, and their choice of clinically appropriate treatment options is optimized. The answers to the following questions are important to improve our understanding of current service provision.

C3.1. Management of hemoglobin level

Please indicate the availability of the following services (tests and treatments) for kidney failure (ESKD) care in your country.
'Generally available' means in 50% or more centres (hospitals or clinics) and 'Generally not available' means: in less than 50% of centres (hospitals or clinics)

C3.1.4. Oral iron	○	○	○	○

C3. Preparation for KRT
Optimal kidney failure (ESKD) care: In the context of the ISN Vision, Mission and Values, we believe all patients approaching kidney failure (ESKD) should receive timely preparation for KRT, so the complications and progression of their disease are minimized, and their choice of clinically appropriate treatment options is optimized. The answers to the following questions are important to improve our understanding of current service provision.

C3.1. Management of hemoglobin level

Please indicate the availability of the following services (tests and treatments) for kidney failure (ESKD) care in your country.
'Generally available' means in 50% or more centres (hospitals or clinics) and 'Generally not available' means: in less than 50% of centres (hospitals or clinics)

C3.1.5. Parenteral iron	◯	◯	◯	◯

C3. Preparation for KRT
Optimal kidney failure (ESKD) care: In the context of the ISN Vision, Mission and Values, we believe all patients approaching kidney failure (ESKD) should receive timely preparation for KRT, so the complications and progression of their disease are minimized, and their choice of clinically appropriate treatment options is optimized. The answers to the following questions are important to improve our understanding of current service provision.

C3.1. Management of hemoglobin level

Please indicate the availability of the following services (tests and treatments) for kidney failure (ESKD) care in your country.
'Generally available' means in 50% or more centres (hospitals or clinics) and 'Generally not available' means: in less than 50% of centres (hospitals or clinics)

C3.1.6. Erythropoiesis stimulating agent (e.g., Erythropoietin)	○	○	○	○

C3.1.2. Management of mineral bone disease

Please indicate the availability of the following services (tests and treatments) for kidney failure (ESKD) care in your country.
'Generally available' means in 50% or more centres (hospitals or clinics) and 'Generally not available' means: in less than 50% of centres (hospitals or clinics).

	Generally available	Generally not available	Never	Unknown
C3.1.2.1. Measurement of serum calcium	◯	◯	◯	◯

C3.1.2. Management of mineral bone disease

Please indicate the availability of the following services (tests and treatments) for kidney failure (ESKD) care in your country.
'Generally available' means in 50% or more centres (hospitals or clinics) and 'Generally not available' means: in less than 50% of centres (hospitals or clinics).

C3.1.2.2. Measurement of serum phosphorus	○	○	○	○

C3.1.2. Management of mineral bone disease

Please indicate the availability of the following services (tests and treatments) for kidney failure (ESKD) care in your country.
'Generally available' means in 50% or more centres (hospitals or clinics) and 'Generally not available' means: in less than 50% of centres (hospitals or clinics).

C3.1.2.3. Measurement of serum parathyroid hormone (PTH)	◯	◯	◯	◯

C3.1.2. Management of mineral bone disease

Please indicate the availability of the following services (tests and treatments) for kidney failure (ESKD) care in your country.
'Generally available' means in 50% or more centres (hospitals or clinics) and 'Generally not available' means: in less than 50% of centres (hospitals or clinics).

C3.1.2.4. Calcium-based phosphate binders	○	○	○	○

C3.1.2. Management of mineral bone disease

Please indicate the availability of the following services (tests and treatments) for kidney failure (ESKD) care in your country.
'Generally available' means in 50% or more centres (hospitals or clinics) and 'Generally not available' means: in less than 50% of centres (hospitals or clinics).

C3.1.2.5. Non-calcium-based phosphate binders (for example, sevelamer)	◯	◯	◯	◯

C3.1.2. Management of mineral bone disease

Please indicate the availability of the following services (tests and treatments) for kidney failure (ESKD) care in your country.
'Generally available' means in 50% or more centres (hospitals or clinics) and 'Generally not available' means: in less than 50% of centres (hospitals or clinics).

C3.1.2.6. Cinacalcet	○	○	○	○

C3.1.2. Management of mineral bone disease

Please indicate the availability of the following services (tests and treatments) for kidney failure (ESKD) care in your country.
'Generally available' means in 50% or more centres (hospitals or clinics) and 'Generally not available' means: in less than 50% of centres (hospitals or clinics).

C3.1.2.7. Surgical services for parathyroidectomy	○	○	○	○

C3.1.3. Management of electrolyte disorders and chronic metabolic acidosis

Please indicate the availability of the following services (tests and treatments) for kidney failure (ESKD) care in your country.
'Generally available' means in 50% or more centres (hospitals or clinics) and 'Generally not available' means: in less than 50% of centres (hospitals or clinics)

	Generally available	Generally not available	Never	Unknown
C3.1.3.1. Measurement of serum electrolytes (sodium, potassium, chloride, etc.)	◯	◯	◯	◯

C3.1.3. Management of electrolyte disorders and chronic metabolic acidosis

Please indicate the availability of the following services (tests and treatments) for kidney failure (ESKD) care in your country.
'Generally available' means in 50% or more centres (hospitals or clinics) and 'Generally not available' means: in less than 50% of centres (hospitals or clinics)

C3.1.3.2. Measurement of serum bicarbonate	◯	◯	◯	◯

C3.1.3. Management of electrolyte disorders and chronic metabolic acidosis

Please indicate the availability of the following services (tests and treatments) for kidney failure (ESKD) care in your country.
'Generally available' means in 50% or more centres (hospitals or clinics) and 'Generally not available' means: in less than 50% of centres (hospitals or clinics)

C3.1.3.3. Potassium exchange resins (for example, Kayexalate, patiromer sodium zirconium)	○	○	○	○

C3.1.3. Management of electrolyte disorders and chronic metabolic acidosis

Please indicate the availability of the following services (tests and treatments) for kidney failure (ESKD) care in your country.
'Generally available' means in 50% or more centres (hospitals or clinics) and 'Generally not available' means: in less than 50% of centres (hospitals or clinics)

C3.1.3.4. Oral sodium bicarbonate	○	○	○	○

C3.1.4. Management of blood pressure

Please indicate the availability of the following services (tests and treatments) for kidney failure (ESKD) care in your country.
'Generally available' means in 50% or more centres (hospitals or clinics) and 'Generally not available' means: in less than 50% of centres (hospitals or clinics)

	Generally available	Generally not available	Never	Unknown
C3.1.4.1. Analogue BP monitoring	○	○	○	○

C3.1.4. Management of blood pressure

Please indicate the availability of the following services (tests and treatments) for kidney failure (ESKD) care in your country.
'Generally available' means in 50% or more centres (hospitals or clinics) and 'Generally not available' means: in less than 50% of centres (hospitals or clinics)

C3.1.4.2. Automated BP monitoring (home or office)	○	○	○	○

C3.1.4. Management of blood pressure

Please indicate the availability of the following services (tests and treatments) for kidney failure (ESKD) care in your country.
'Generally available' means in 50% or more centres (hospitals or clinics) and 'Generally not available' means: in less than 50% of centres (hospitals or clinics)

C3.1.4.3. Ambulatory BP monitoring (ABPM)	○	○	○	○

C3.1.5. Management of common kidney failure (ESKD)-associated symptoms (uremic pruritus, restless legs, pain)

Please indicate the availability of the following services (tests and treatments) for kidney failure (ESKD) care in your country.
'Generally available' means in 50% or more centres (hospitals or clinics) and 'Generally not available' means: in less than 50% of centres (hospitals or clinics)

	Generally available	Generally not available	Never	Unknown
C3.1.5.1. Gabapentinoids (gabapentin or pregabalin)	○	○	○	○

C3.1.5. Management of common kidney failure (ESKD)-associated symptoms (uremic pruritus, restless legs, pain)

Please indicate the availability of the following services (tests and treatments) for kidney failure (ESKD) care in your country.
'Generally available' means in 50% or more centres (hospitals or clinics) and 'Generally not available' means: in less than 50% of centres (hospitals or clinics)

C3.1.5.2. Non-morphine opioids (e.g., hydromorphone, oxycodone, methadone, and/or sublingual or transdermal fentanyl)	○	○	○	○

C4.1 Nutritional services

C4.1.1. Please indicate the availability of the following nutritional services for kidney care in your country.
'Generally available' means in 50% or more centres (hospitals or clinics) and 'Generally not available' means: in less than 50% of centres (hospitals or clinics)

	Generally available	Generally not available	Never	Unknown
C4.1.1.1. Dietary counselling by a person trained in nutrition (for example, a dietitian)	◯	◯	◯	◯

C4.1 Nutritional services

C4.1.1. Please indicate the availability of the following nutritional services for kidney care in your country.
'Generally available' means in 50% or more centres (hospitals or clinics) and 'Generally not available' means: in less than 50% of centres (hospitals or clinics)

C4.1.1.2. Measurement of serum albumin	○	○	○	○

C4.1 Nutritional services

C4.1.1. Please indicate the availability of the following nutritional services for kidney care in your country.
'Generally available' means in 50% or more centres (hospitals or clinics) and 'Generally not available' means: in less than 50% of centres (hospitals or clinics)

C4.1.1.3. Oral nutrition supplements (for example, vitamins, oral meal supplements)	○	○	○	○

C5. Dialysis treatment - Quality and Choice

C5.1. Modality Choice

Please indicate the availability of the following services for dialysis care in your country. 'Generally available' means in 50% or more centres (hospitals or clinics) and 'Generally not available' means: in less than 50% of centres (hospitals or clinics).

	Generally available	Generally not available	Never	Unknown	N/A (dialysis not provided)
C5.1.1. Centre-based Hemodialysis	◯	◯	◯	◯	◯

C5. Dialysis treatment - Quality and Choice

C5.1. Modality Choice

Please indicate the availability of the following services for dialysis care in your country. 'Generally available' means in 50% or more centres (hospitals or clinics) and 'Generally not available' means: in less than 50% of centres (hospitals or clinics).

C5.1.2. Home hemodialysis	○	○	○	○	○

C5. Dialysis treatment - Quality and Choice

C5.1. Modality Choice

Please indicate the availability of the following services for dialysis care in your country. 'Generally available' means in 50% or more centres (hospitals or clinics) and 'Generally not available' means: in less than 50% of centres (hospitals or clinics).

C5.1.3. Peritoneal dialysis	○	○	○	○	○

C5.1. Quality

Please indicate the availability of the following services for dialysis care in your country. 'Generally available' means in 50% or more centres (hospitals or clinics) and 'Generally not available' means: in less than 50% of centres (hospitals or clinics).

	Generally available	Generally not available	Never	Unknown	N/A (dialysis not provided)
C5.1.4. Centre-based hemodialysis service of adequate frequency (treatment three times a week for three or four hours)	○	○	○	○	○

C5.1. Quality

Please indicate the availability of the following services for dialysis care in your country. 'Generally available' means in 50% or more centres (hospitals or clinics) and 'Generally not available' means: in less than 50% of centres (hospitals or clinics).

C5.1.5. Home hemodialysis (treatment three times a week for three or four hours)	○	○	○	○	○

C5.1. Quality

Please indicate the availability of the following services for dialysis care in your country. 'Generally available' means in 50% or more centres (hospitals or clinics) and 'Generally not available' means: in less than 50% of centres (hospitals or clinics).

C5.1.6. Peritoneal dialysis exchanges of adequate frequency (3-4 per day or equivalent cycles on automated PD)	○	○	○	○	○

C5.1. Quality

Please indicate the availability of the following services for dialysis care in your country. 'Generally available' means in 50% or more centres (hospitals or clinics) and 'Generally not available' means: in less than 50% of centres (hospitals or clinics).

C5.1.7. Determination of the effectiveness of peritoneal dialysis (that is, by measurement of urea reduction ratio [URR] and/or Kt/V)	◯	◯	◯	◯	◯

C5.1. Quality

Please indicate the availability of the following services for dialysis care in your country. 'Generally available' means in 50% or more centres (hospitals or clinics) and 'Generally not available' means: in less than 50% of centres (hospitals or clinics).

C5.1.8. Affordable patient transport services	○	○	○	○	○

C6. Transplant - Quality & Choice
Transplant choice: In the context of the ISN Vision, Mission and Values, we believe all patients with kidney transplant are to receive a high-quality service which supports them in managing their transplant and enables them to achieve the best possible quality of life. The answers to the following questions are important to improve our understanding of current service provision.

C6.1. Please indicate the availability of the following services for transplantation services in your country.
'Generally available' means in 50% or more centres (hospitals or clinics) and 'Generally not available' means: in less than 50% of centres (hospitals or clinics).

If transplantation is NOT available in your country, select N/A.

	Generally available	Generally not available	Never	Unknown	N/A (transplantation not available)
C6.1.1. Early provision of culturally appropriate information to patients, relatives and caregivers about the risks and benefits of transplantation with a clear explanation of tests, procedures, and results	○	○	○	○	○

C6. Transplant - Quality & Choice
Transplant choice: In the context of the ISN Vision, Mission and Values, we believe all patients with kidney transplant are to receive a high-quality service which supports them in managing their transplant and enables them to achieve the best possible quality of life. The answers to the following questions are important to improve our understanding of current service provision.

C6.1. Please indicate the availability of the following services for transplantation services in your country.
'Generally available' means in 50% or more centres (hospitals or clinics) and 'Generally not available' means: in less than 50% of centres (hospitals or clinics).

If transplantation is NOT available in your country, select N/A.

C6.1.2. Effective preventive therapy to control infections (for example, antivirals, antifungals, etc.)	○	○	○	○	○

C6. Transplant - Quality & Choice

Transplant choice: In the context of the ISN Vision, Mission and Values, we believe all patients with kidney transplant are to receive a high-quality service which supports them in managing their transplant and enables them to achieve the best possible quality of life. The answers to the following questions are important to improve our understanding of current service provision.

C6.1. Please indicate the availability of the following services for transplantation services in your country.
'Generally available' means in 50% or more centres (hospitals or clinics) and 'Generally not available' means: in less than 50% of centres (hospitals or clinics).

If transplantation is NOT available in your country, select N/A.

C6.1.3. Timely access to operating space for kidney transplantation	○	○	○	○	○

C6. Transplant - Quality & Choice

Transplant choice: In the context of the ISN Vision, Mission and Values, we believe all patients with kidney transplant are to receive a high-quality service which supports them in managing their transplant and enables them to achieve the best possible quality of life. The answers to the following questions are important to improve our understanding of current service provision.

C6.1. Please indicate the availability of the following services for transplantation services in your country.
'Generally available' means in 50% or more centres (hospitals or clinics) and 'Generally not available' means: in less than 50% of centres (hospitals or clinics).

If transplantation is NOT available in your country, select N/A.

C6.1.4. Appropriate immunosuppression and anti-rejection treatment	○	○	○	○	○

C6. Transplant - Quality & Choice
Transplant choice: In the context of the ISN Vision, Mission and Values, we believe all patients with kidney transplant are to receive a high-quality service which supports them in managing their transplant and enables them to achieve the best possible quality of life. The answers to the following questions are important to improve our understanding of current service provision.

C6.1. Please indicate the availability of the following services for transplantation services in your country.
'Generally available' means in 50% or more centres (hospitals or clinics) and 'Generally not available' means: in less than 50% of centres (hospitals or clinics).

If transplantation is NOT available in your country, select N/A.

C6.1.5. Appropriate facilities to monitor administration of immunosuppression drugs	○	○	○	○	○

C6. Transplant - Quality & Choice
Transplant choice: In the context of the ISN Vision, Mission and Values, we believe all patients with kidney transplant are to receive a high-quality service which supports them in managing their transplant and enables them to achieve the best possible quality of life. The answers to the following questions are important to improve our understanding of current service provision.

C6.1. Please indicate the availability of the following services for transplantation services in your country.
'Generally available' means in 50% or more centres (hospitals or clinics) and 'Generally not available' means: in less than 50% of centres (hospitals or clinics).

If transplantation is NOT available in your country, select N/A.

C6.1.6. Multidisciplinary team to support patients with a kidney transplant	○	○	○	○	○

C6. Transplant - Quality & Choice
Transplant choice: In the context of the ISN Vision, Mission and Values, we believe all patients with kidney transplant are to receive a high-quality service which supports them in managing their transplant and enables them to achieve the best possible quality of life. The answers to the following questions are important to improve our understanding of current service provision.

C6.1. Please indicate the availability of the following services for transplantation services in your country.
'Generally available' means in 50% or more centres (hospitals or clinics) and 'Generally not available' means: in less than 50% of centres (hospitals or clinics).

If transplantation is NOT available in your country, select N/A.

C6.1.7. Standard framework for organ procurement (for example, legislation around brain death)	○	○	○	○	○

C7. Conservative Kidney Management (CKM)

Conservative Kidney Management: Conservative kidney management is defined as the patient's choice for holistic, patient-centered care without the use of KRT for those with CKD stage G5. The goals of conservative kidney management are to support patients with CKD stage G5 who are not receiving KRT by optimizing quality of life, managing symptoms, treating psychosocial distress, facilitating advance care planning and, where appropriate, preserving residual kidney function. This care includes supporting the family and carers of the patient and continues throughout the illness trajectory. This is appropriate for patients who are unlikely to benefit from KRT or who choose not to initiate KRT. We recognize that patients may receive similar conservative care when resource constraints (healthcare system or patient) prevent or limit access to KRT. We term this choice-restricted conservative care.

We would like to know more about the capacity to deliver conservative kidney management or choice restricted conservative care in your country (that is, the capacity to support/manage patients who will not receive KRT despite having CKD stage G5).

C7.1. Considering the definitions above, is conservative care available in your country?

	Generally available	Generally not available	N/A (CKM not available)	Unknown
C7.1.1. Established conservative kidney management that is chosen through shared decision making (where KRT is readily available)	○	○	○	○

C7. Conservative Kidney Management (CKM)

Conservative Kidney Management: Conservative kidney management is defined as the patient's choice for holistic, patient-centered care without the use of KRT for those with CKD stage G5. The goals of conservative kidney management are to support patients with CKD stage G5 who are not receiving KRT by optimizing quality of life, managing symptoms, treating psychosocial distress, facilitating advance care planning and, where appropriate, preserving residual kidney function. This care includes supporting the family and carers of the patient and continues throughout the illness trajectory. This is appropriate for patients who are unlikely to benefit from KRT or who choose not to initiate KRT. We recognize that patients may receive similar conservative care when resource constraints (healthcare system or patient) prevent or limit access to KRT. We term this choice-restricted conservative care.

We would like to know more about the capacity to deliver conservative kidney management or choice restricted conservative care in your country (that is, the capacity to support/manage patients who will not receive KRT despite having CKD stage G5).

C7.1. Considering the definitions above, is conservative care available in your country?

C7.1.2. Established choice-restricted conservative care (where resource constraints to prevent or limit access to KRT)	○	○	○	○

C7. Conservative Kidney Management (CKM)

Conservative Kidney Management: Conservative kidney management is defined as the patient's choice for holistic, patient-centered care without the use of KRT for those with CKD stage G5. The goals of conservative kidney management are to support patients with CKD stage G5 who are not receiving KRT by optimizing quality of life, managing symptoms, treating psychosocial distress, facilitating advance care planning and, where appropriate, preserving residual kidney function. This care includes supporting the family and carers of the patient and continues throughout the illness trajectory. This is appropriate for patients who are unlikely to benefit from KRT or who choose not to initiate KRT. We recognize that patients may receive similar conservative care when resource constraints (healthcare system or patient) prevent or limit access to KRT. We term this choice-restricted conservative care.

We would like to know more about the capacity to deliver conservative kidney management or choice restricted conservative care in your country (that is, the capacity to support/manage patients who will not receive KRT despite having CKD stage G5).

C7.1. Considering the definitions above, is conservative care available in your country?

C7.1.3. Established choice-restricted conservative care (where there are no resource constraints to prevent or limit access to KRT)	◯	◯	◯	◯

C7.2. Please indicate the average likelihood of a nephrologist from your country offering conservative kidney management as a treatment option to patients with CKD stage G5?

- ◯ Always
- ◯ Often
- ◯ Sometimes
- ◯ Rarely
- ◯ Never
- ◯ I don't know

C7.3. Where access to dialysis is choice restricted, what is the principal reason

- ◯ Financial - healthcare system
- ◯ Financial - patient
- ◯ Geographic

C7.4. Please indicate the availability of the structure and process for the delivery of conservative kidney management (i.e., conservative care that is chosen or medically advised where KRT is readily available) for patients with CKD stage G5):
'Generally available' means in 50% or more centres (hospitals or clinics) and 'Generally not available' means: in less than 50% of centres (hospitals or clinics).

	Generally available	Generally not available	Not available	Unknown
C7.4.1. Established infrastructure to support patients on a conservative kidney management pathway	○	○	○	○

C7.4. Please indicate the availability of the structure and process for the delivery of conservative kidney management (i.e., conservative care that is chosen or medically advised where KRT is readily available) for patients with CKD stage G5):
'Generally available' means in 50% or more centres (hospitals or clinics) and 'Generally not available' means: in less than 50% of centres (hospitals or clinics).

C7.4.2. Shared decision-making tools for patients and providers to help make the decision for conservative kidney management	○	○	○	○

C7.4. Please indicate the availability of the structure and process for the delivery of conservative kidney management (i.e., conservative care that is chosen or medically advised where KRT is readily available) for patients with CKD stage G5):
'Generally available' means in 50% or more centres (hospitals or clinics) and 'Generally not available' means: in less than 50% of centres (hospitals or clinics).

C7.4.3. Established services where patients receiving conservative kidney management can be seen in home / care home / hospice if unable to attend hospital or clinic	○	○	○	○

C7.4. Please indicate the availability of the structure and process for the delivery of conservative kidney management (i.e., conservative care that is chosen or medically advised where KRT is readily available) for patients with CKD stage G5):
'Generally available' means in 50% or more centres (hospitals or clinics) and 'Generally not available' means: in less than 50% of centres (hospitals or clinics).

C7.4.4. A written pathway / blueprint / or guidelines for conservative kidney management encompassing preservation of residual kidney function, symptom control, advance care planning, and end of life care	○	○	○	○

C7.4. Please indicate the availability of the structure and process for the delivery of conservative kidney management (i.e., conservative care that is chosen or medically advised where KRT is readily available) for patients with CKD stage G5):
'Generally available' means in 50% or more centres (hospitals or clinics) and 'Generally not available' means: in less than 50% of centres (hospitals or clinics).

C7.4.5. A multidisciplinary team	○	○	○	○

C7.4. Please indicate the availability of the structure and process for the delivery of conservative kidney management (i.e., conservative care that is chosen or medically advised where KRT is readily available) for patients with CKD stage G5):
'Generally available' means in 50% or more centres (hospitals or clinics) and 'Generally not available' means: in less than 50% of centres (hospitals or clinics).

C7.4.6. The multidisciplinary team includes formal links with kidney clinicians trained in conservative care	◯	◯	◯	◯

C7.4. Please indicate the availability of the structure and process for the delivery of conservative kidney management (i.e., conservative care that is chosen or medically advised where KRT is readily available) for patients with CKD stage G5):
'Generally available' means in 50% or more centres (hospitals or clinics) and 'Generally not available' means: in less than 50% of centres (hospitals or clinics).

C7.4.7. The multidisciplinary team includes formal links with palliative care	○	○	○	○

C7.4. Please indicate the availability of the structure and process for the delivery of conservative kidney management (i.e., conservative care that is chosen or medically advised where KRT is readily available) for patients with CKD stage G5):
'Generally available' means in 50% or more centres (hospitals or clinics) and 'Generally not available' means: in less than 50% of centres (hospitals or clinics).

C7.4.8. Regular use of validated screening tools, documentation, and management of symptoms	○	○	○	○

C7.4. Please indicate the availability of the structure and process for the delivery of conservative kidney management (i.e., conservative care that is chosen or medically advised where KRT is readily available) for patients with CKD stage G5):
'Generally available' means in 50% or more centres (hospitals or clinics) and 'Generally not available' means: in less than 50% of centres (hospitals or clinics).

C7.4.9. Availability of essential medicines for pain and palliative care at all levels of care (primary and specialty)	○	○	○	○

C7.4. Please indicate the availability of the structure and process for the delivery of conservative kidney management (i.e., conservative care that is chosen or medically advised where KRT is readily available) for patients with CKD stage G5):
'Generally available' means in 50% or more centres (hospitals or clinics) and 'Generally not available' means: in less than 50% of centres (hospitals or clinics).

C7.4.10. infrastructure to document and share advance care planning conversations including decisions around preferred place of care and death and resuscitation.	○	○	○	○

C7.4. Please indicate the availability of the structure and process for the delivery of conservative kidney management (i.e., conservative care that is chosen or medically advised where KRT is readily available) for patients with CKD stage G5):
'Generally available' means in 50% or more centres (hospitals or clinics) and 'Generally not available' means: in less than 50% of centres (hospitals or clinics).

C7.4.11. Provision of psychological, social, and spiritual support	○	○	○	○

C7.4. Please indicate the availability of the structure and process for the delivery of conservative kidney management (i.e., conservative care that is chosen or medically advised where KRT is readily available) for patients with CKD stage G5):
'Generally available' means in 50% or more centres (hospitals or clinics) and 'Generally not available' means: in less than 50% of centres (hospitals or clinics).

C7.4.12. Training of care providers in symptom management	○	○	○	○

C7.4. Please indicate the availability of the structure and process for the delivery of conservative kidney management (i.e., conservative care that is chosen or medically advised where KRT is readily available) for patients with CKD stage G5):
'Generally available' means in 50% or more centres (hospitals or clinics) and 'Generally not available' means: in less than 50% of centres (hospitals or clinics).

C7.4.13. Training of care providers in advance care planning	◯	◯	◯	◯

C7.4. Please indicate the availability of the structure and process for the delivery of conservative kidney management (i.e., conservative care that is chosen or medically advised where KRT is readily available) for patients with CKD stage G5):
'Generally available' means in 50% or more centres (hospitals or clinics) and 'Generally not available' means: in less than 50% of centres (hospitals or clinics).

C7.4.14. Systematic data collection on numbers of patients receiving conservative kidney management and their outcomes	○	○	○	○

C7.5. Please indicate the availability of the structure and process for the delivery of choice-restricted conservative care (i.e., conservative care for patients in whom resource constraints prevent or limit access to KRT) for patients with CKD stage G5):
'Generally available' means in 50% or more centres (hospitals or clinics) and 'Generally not available' means: in less than 50% of centres (hospitals or clinics).

	Generally available	Generally not available	Not available	Unknown
C7.5.1. Established infrastructure to support patients receiving choice-restricted conservative care	◯	◯	◯	◯

C7.5. Please indicate the availability of the structure and process for the delivery of choice-restricted conservative care (i.e., conservative care for patients in whom resource constraints prevent or limit access to KRT) for patients with CKD stage G5):
'Generally available' means in 50% or more centres (hospitals or clinics) and 'Generally not available' means: in less than 50% of centres (hospitals or clinics).

C7.5.2. Established services where patients receiving choice-restricted conservative care can be seen in home / care home / hospice if unable to attend hospital or clinic	○	○	○	○

C7.5. Please indicate the availability of the structure and process for the delivery of choice-restricted conservative care (i.e., conservative care for patients in whom resource constraints prevent or limit access to KRT) for patients with CKD stage G5):
'Generally available' means in 50% or more centres (hospitals or clinics) and 'Generally not available' means: in less than 50% of centres (hospitals or clinics).

C7.5.3. A written pathway / blueprint / or guidelines for conservative care encompassing preservation of residual kidney function, symptom control, advance care planning, and end of life care	○	○	○	○

C7.5. Please indicate the availability of the structure and process for the delivery of choice-restricted conservative care (i.e., conservative care for patients in whom resource constraints prevent or limit access to KRT) for patients with CKD stage G5):
'Generally available' means in 50% or more centres (hospitals or clinics) and 'Generally not available' means: in less than 50% of centres (hospitals or clinics).

C7.5.4. A multidisciplinary team	○	○	○	○

C7.5. Please indicate the availability of the structure and process for the delivery of choice-restricted conservative care (i.e., conservative care for patients in whom resource constraints prevent or limit access to KRT) for patients with CKD stage G5):
'Generally available' means in 50% or more centres (hospitals or clinics) and 'Generally not available' means: in less than 50% of centres (hospitals or clinics).

C7.5.5. The multidisciplinary team includes formal links with kidney clinicians trained in conservative care	○	○	○	○

C7.5. Please indicate the availability of the structure and process for the delivery of choice-restricted conservative care (i.e., conservative care for patients in whom resource constraints prevent or limit access to KRT) for patients with CKD stage G5):
'Generally available' means in 50% or more centres (hospitals or clinics) and 'Generally not available' means: in less than 50% of centres (hospitals or clinics).

C7.5.6. The multidisciplinary team includes formal links with palliative care	○	○	○	○

C7.5. Please indicate the availability of the structure and process for the delivery of choice-restricted conservative care (i.e., conservative care for patients in whom resource constraints prevent or limit access to KRT) for patients with CKD stage G5):
'Generally available' means in 50% or more centres (hospitals or clinics) and 'Generally not available' means: in less than 50% of centres (hospitals or clinics).

C7.5.7. Regular use of validated screening tools, documentation, and management of symptoms	○	○	○	○

C7.5. Please indicate the availability of the structure and process for the delivery of choice-restricted conservative care (i.e., conservative care for patients in whom resource constraints prevent or limit access to KRT) for patients with CKD stage G5):
'Generally available' means in 50% or more centres (hospitals or clinics) and 'Generally not available' means: in less than 50% of centres (hospitals or clinics).

C7.5.8. Availability of essential medicines for pain and palliative care at all levels of care (primary and specialty)	○	○	○	○

C7.5. Please indicate the availability of the structure and process for the delivery of choice-restricted conservative care (i.e., conservative care for patients in whom resource constraints prevent or limit access to KRT) for patients with CKD stage G5):
'Generally available' means in 50% or more centres (hospitals or clinics) and 'Generally not available' means: in less than 50% of centres (hospitals or clinics).

C7.5.9. Infrastructure to document and share advance care planning conversations including decisions around preferred place of care and death and resuscitation.	○	○	○	○

C7.5. Please indicate the availability of the structure and process for the delivery of choice-restricted conservative care (i.e., conservative care for patients in whom resource constraints prevent or limit access to KRT) for patients with CKD stage G5):
'Generally available' means in 50% or more centres (hospitals or clinics) and 'Generally not available' means: in less than 50% of centres (hospitals or clinics).

C7.5.10. Provision of psychological, social, and spiritual support	○	○	○	○

C7.5. Please indicate the availability of the structure and process for the delivery of choice-restricted conservative care (i.e., conservative care for patients in whom resource constraints prevent or limit access to KRT) for patients with CKD stage G5):
'Generally available' means in 50% or more centres (hospitals or clinics) and 'Generally not available' means: in less than 50% of centres (hospitals or clinics).

C7.5.11. Training of care providers in symptom management	○	○	○	○

C7.5. Please indicate the availability of the structure and process for the delivery of choice-restricted conservative care (i.e., conservative care for patients in whom resource constraints prevent or limit access to KRT) for patients with CKD stage G5):
'Generally available' means in 50% or more centres (hospitals or clinics) and 'Generally not available' means: in less than 50% of centres (hospitals or clinics).

C7.5.12. Training of care providers in advance care planning	○	○	○	○

C7.5. Please indicate the availability of the structure and process for the delivery of choice-restricted conservative care (i.e., conservative care for patients in whom resource constraints prevent or limit access to KRT) for patients with CKD stage G5):
'Generally available' means in 50% or more centres (hospitals or clinics) and 'Generally not available' means: in less than 50% of centres (hospitals or clinics).

C7.5.13. Systematic data collection on numbers of patients receiving choice-restricted conservative care and their outcomes	○	○	○	○

C8. Affordability

C8.1. What is the national average co-payment (including medications but no other ancillaries) for hemodialysis patients in your country (that is, the proportion of the treatment cost paid for directly (out-of-pocket) by the patient?

- ◯ N/A (not available in my country)
- ◯ 0%
- ◯ 1-25%
- ◯ 26-50%
- ◯ 51-75%
- ◯ >75%
- ◯ 100%

C8.1.1. Does this proportion vary in different parts of the country?

- ◯ Yes (please explain below)
- ◯ No
- ◯ Other (please explain below)

C8.1.1. If other, please explain.

____

C8.1.1. If yes, please explain.

____

C8.1.2. Does this proportion vary depending on patients' characteristics (for example, age, gender, employment status)?

- ◯ Yes (please explain below)
- ◯ No
- ◯ Other (please explain below)

C8.1.2. If yes, please explain.

____

C8.1.2. If other, please explain.

____

C8.1.3. What proportion (national average) of patients with kidney failure (ESKD) (kidney failure) on hemodialysis withdraw dialysis within a year due to financial reasons in your country?

- ◯ N/A (not available in my country)
- ◯ 0%
- ◯ 1-10%
- ◯ 11-25%
- ◯ 26-50%
- ◯ >50%

C8.2. What is the national average co-payment (including medications but no other ancillaries) for peritoneal dialysis patients in your country, that is, the proportion of the treatment cost paid for directly (out-of-pocket) by the patient?

- ◯ N/A (not available in my country)
- ◯ 0%
- ◯ 1-25%
- ◯ 26-50%
- ◯ 51-75%
- ◯ >75%
- ◯ 100%

C8.2.1. Does this proportion vary in different parts of the country?

- ◯ Yes (please explain below)
- ◯ No
- ◯ Other (please explain below)

C8.2.1. If yes, please explain.

____

C8.2.1. If other, please explain.

____

C8.2.2. Does this proportion vary depending on patients' characteristics (for example, age, gender, employment status)?

◯ Yes (please explain below)
◯ No
◯ Other (please explain below)

C8.2.2. If yes, please explain.

____

C8.2.2. If other, please explain.

____

C8.3. What is the national average co-payment (including medications but no other ancillaries) for kidney transplant patients in your country, that is, the proportion of the treatment cost paid for directly (out-of-pocket) by the patient?

◯ N/A (not available in my country)
◯ 0%
◯ 1-25%
◯ 26-50%
◯ 51-75%
◯ >75%
◯ 100%

C8.3.1. Does this proportion vary in different parts of the country?

◯ Yes (please explain below)
◯ No
◯ Other (please explain below)

C8.3.1. If yes, please explain.

____

C8.3.1. If other, please explain.

____

C8.3.2. Does this proportion vary depending on patients' characteristics (for example, age, gender, employment status)?

◯ Yes (please explain below)
◯ No
◯ Other (please explain below)

C8.3.2. If yes, please explain.

____

C8.3.2. If other, please explain.

____

C8.4. What proportion (national average) of patients with kidney failure (ESKD) are able to access dialysis in your country?

◯ N/A (not available in my country)
◯ 1-10%
◯ 11-25%
◯ 26-50%
◯ >50%

C8.4.1. Does this proportion vary in different parts of the country?

◯ Yes (please explain below)
◯ No
◯ Other (please explain below)

C8.4.1. If yes, please explain.

____

C8.4.1. If other, please explain.

____

C8.4.2. Does this proportion vary depending on patients' characteristics (for example, age, gender, employment status)?

◯ Yes (please explain below)
◯ No
◯ Other (please explain below)

C8.4.2. If yes, please explain.

____

C8.4.2. If other, please explain.

____

C8.5. Out of those patients in your country who have kidney failure (ESKD) and are able to access dialysis, what proportion usually start with peritoneal dialysis?

◯ N/A - dialysis (of any kind) is not available in my country
◯ 0% (means that there are patients who are able to access some form of dialysis, but none of them start with PD)
◯ 1-10%
◯ 11-25%
◯ 26-50%
◯ >50%

C8.5.1. Does this proportion vary in different parts of the country?

◯ Yes (please explain below)
◯ No
◯ Other (please explain below)

C8.5.1. If yes, please explain.

____

C8.5.1. If other, please explain.

____

C8.5.2. Does this proportion vary depending on patients' characteristics (for example, age, gender, employment status)?

◯ Yes (please explain below)
◯ No
◯ Other (please explain below)

C8.5.2. If yes, please explain.

____

C8.5.2. If other, please explain.

____

C8.6. Out of those patients in your country who have kidney failure (ESKD) (kidney failure) and are suitable for transplant, what proportion are able to access kidney transplantation?	◯ 0% (not available in my country)<br>◯ 1-10%<br>◯ 11-25%<br>◯ 26-50%<br>◯ >50%
C8.6.1. Does this proportion vary in different parts of the country?	◯ Yes (please explain below)<br>◯ No<br>◯ Other (please explain below)
C8.6.1. If yes, please explain.	__________________________________
C8.6.1. If other, please explain.	__________________________________
C8.6.2. Does this proportion vary depending on patients' characteristics (for example, age, gender, employment status)?	◯ Yes (please explain below)<br>◯ No<br>◯ Other (please explain below)
C8.6.2. If yes, please explain.	__________________________________
C8.6.2. If other, please explain.	__________________________________

C9. Peritoneal Dialysis Quality
If peritoneal dialysis is available in your country, what proportion of centres routinely measure and report the following to assess the quality of the dialysis that is provided?

C9.1. What proportion of PD patients start with less than full dose PD (i.e., incremental)? (less than full dose is considered < 8L/day)

- 0%
- 1-10%
- 11-25%
- 26-50%
- >50%

C9.2. Patient-reported outcome measures (for example, fatigue, quality of life, satisfaction, pain):

- 0% (None)
- 1-10% (Few)
- 11-50% (Some)
- 51-75% (Most)
- >75% (Almost all)

C9.3. Blood pressure:

- 0% (None)
- 1-10% (Few)
- 11-50% (Some)
- 51-75% (Most)
- >75% (Almost all)

C9.4. Small solute clearance (for example, Kt/V or creatinine clearance):

- 0% (None)
- 1-10% (Few)
- 11-50% (Some)
- 51-75% (Most)
- >75% (Almost all)

C9.5. Hemoglobin/hematocrit:

- 0% (None)
- 1-10% (Few)
- 11-50% (Some)
- 51-75% (Most)
- >75% (Almost all)

C9.6. Bone mineral markers (calcium, phosphate, parathyroid hormone [PTH]):

- 0% (None)
- 1-10% (Few)
- 11-50% (Some)
- 51-75% (Most)
- >75% (Almost all)

C9.7. Technique survival (Transfer to HD):

- 0% (None)
- 1-10% (Few)
- 11-50% (Some)
- 51-75% (Most)
- >75% (Almost all)

C9.8. Patient survival:

- 0% (None)
- 1-10% (Few)
- 11-50% (Some)
- 51-75% (Most)
- >75% (Almost all)

C9.9 What is the nursing staff to patient ratio per shift in ≥ 50% of peritoneal dialysis centres in your country?

- 1:1 to 1:3
- 1:4 to 1:6
- 1:7 to 1:9
- 1:10 to 1:13
- ≥1:14
- unknown

C10. Hemodialysis quality
If hemodialysis is available in your country, what proportion of centres routinely measure and report the following to assess the quality of the dialysis that is provided?

C10.1. What proportion of HD patients start with less than 3x/week of HD (i.e., incremental HD)?	◯ 0%<br>◯ 1-10%<br>◯ 11-25%<br>◯ 26-50%<br>◯ >50%
C10.2. Patient-reported outcome measures (for example, fatigue, quality of life, satisfaction, pain):	◯ 0% (None)<br>◯ 1-10% (Few)<br>◯ 11-50% (Some)<br>◯ 51-75% (Most)<br>◯ >75% (Almost all)
C10.3. Blood pressure:	◯ 0% (None)<br>◯ 1-10% (Few)<br>◯ 11-50% (Some)<br>◯ 51-75% (Most)<br>◯ >75% (Almost all)
C10.4. Small solute clearance (for example, Kt/V or creatinine clearance):	◯ 0% (None)<br>◯ 1-10% (Few)<br>◯ 11-50% (Some)<br>◯ 51-75% (Most)<br>◯ >75% (Almost all)
C10.5. Hemoglobin/hematocrit:	◯ 0% (None)<br>◯ 1-10% (Few)<br>◯ 11-50% (Some)<br>◯ 51-75% (Most)<br>◯ >75% (Almost all)
C10.6. Bone mineral markers (calcium, phosphate, parathyroid hormone [PTH]):	◯ 0% (None)<br>◯ 1-10% (Few)<br>◯ 11-50% (Some)<br>◯ 51-75% (Most)<br>◯ >75% (Almost all)
C10.7. Technique survival:	◯ 0% (None)<br>◯ 1-10% (Few)<br>◯ 11-50% (Some)<br>◯ 51-75% (Most)<br>◯ >75% (Almost all)
C10.8. Patient survival:	◯ 0% (None)<br>◯ 1-10% (Few)<br>◯ 11-50% (Some)<br>◯ 51-75% (Most)<br>◯ >75% (Almost all)
C10.9. Monitoring Hepatitis B and C and HIV virology at least twice a year:	◯ 0% (None)<br>◯ 1-10% (Few)<br>◯ 11-50% (Some)<br>◯ 51-75% (Most)<br>◯ >75% (Almost all)

C10.10. Regular monitoring of dialysis water quality for bacteria and chemical components as per AAMI or equivalent national regulatory body recommendation:	◯ 0% (None)<br>◯ 1-10% (Few)<br>◯ 11-50% (Some)<br>◯ 51-75% (Most)<br>◯ >75% (Almost all)
C10.11. Regular HD patient review by nephrologist at least once every 3 months:	◯ 0% (None)<br>◯ 1-10% (Few)<br>◯ 11-50% (Some)<br>◯ 51-75% (Most)<br>◯ >75% (Almost all)
C10.12. What is the nursing staff to patient ratio per shift in ≥ 50% of hemodialysis centres in your country?	◯ 1:1 to 1:3<br>◯ 1:4 to 1:6<br>◯ 1:7 to 1:9<br>◯ 1:10 to 1:13<br>◯ ≥1:14<br>◯ unknown

C11. Kidney Transplantation Quality
If kidney transplantation is available in your country, what proportion of centres routinely measure and report the following to assess the quality of the transplantation that is provided?

C11.1. Patient-reported outcome measures (for example, fatigue, quality of life, satisfaction, pain, etc.):	◯ 0% (None)<br>◯ 1-10% (Few)<br>◯ 11-50% (Some)<br>◯ 51-75% (Most)<br>◯ >75% (Almost all)<br>◯ unknown
C11.2. Delayed graft function:	◯ 0% (None)<br>◯ 1-10% (Few)<br>◯ 11-50% (Some)<br>◯ 51-75% (Most)<br>◯ >75% (Almost all)<br>◯ unknown
C11.3. Rejection rates:	◯ 0% (None)<br>◯ 1-10% (Few)<br>◯ 11-50% (Some)<br>◯ 51-75% (Most)<br>◯ >75% (Almost all)<br>◯ unknown
C11.4. Kidney allograft function:	◯ 0% (None)<br>◯ 1-10% (Few)<br>◯ 11-50% (Some)<br>◯ 51-75% (Most)<br>◯ >75% (Almost all)<br>◯ unknown
C11.5. Graft survival:	◯ 0% (None)<br>◯ 1-10% (Few)<br>◯ 11-50% (Some)<br>◯ 51-75% (Most)<br>◯ >75% (Almost all)<br>◯ unknown
C11.6. Patient survival:	◯ 0% (None)<br>◯ 1-10% (Few)<br>◯ 11-50% (Some)<br>◯ 51-75% (Most)<br>◯ >75% (Almost all)<br>◯ unknown

C12. Access

C12.1. For hemodialysis, what proportion of patients routinely start dialysis with a functioning vascular access (AV fistula or graft):

- ◯ 0% (None)
- ◯ 1-10% (Few)
- ◯ 11-50% (Some)
- ◯ 51-75% (Most)
- ◯ >75% (Almost all)
- ◯ unknown

C12.2. For hemodialysis, what proportion of patients routinely start dialysis with a tunnelled dialysis catheter:

- ◯ 0% (None)
- ◯ 1-10% (Few)
- ◯ 11-50% (Some)
- ◯ 51-75% (Most)
- ◯ >75% (Almost all)
- ◯ unknown

C12.3. For hemodialysis, what proportion of patients commonly start dialysis with a temporary dialysis catheter:

- ◯ 0% (None)
- ◯ 1-10% (Few)
- ◯ 11-50% (Some)
- ◯ 51-75% (Most)
- ◯ >75% (Almost all)
- ◯ unknown

C12.4. For hemodialysis, what proportion of prevalent patients dialyse with a functioning vascular access (AV fistula or graft):

- ◯ 0% (None)
- ◯ 1-10% (Few)
- ◯ 11-50% (Some)
- ◯ 51-75% (Most)
- ◯ >75% (Almost all)
- ◯ unknown

Access for all dialysis - answer only if hemodialysis or peritoneal dialysis is available in your country

C12.5. For either hemodialysis or peritoneal dialysis, what proportion of patients routinely receive education about the best means of access and timely surgery (for example, six months before start of hemodialysis, one month before start of peritoneal dialysis):

- ◯ 0% (None)
- ◯ 1-10% (Few)
- ◯ 11-50% (Some)
- ◯ 51-75% (Most)
- ◯ >75% (Almost all)
- ◯ unknown

C13. Outcomes (hemodialysis)

C13.1. What proportion (national average) of patients with kidney failure (ESKD) (kidney failure) on hemodialysis died in the first year of dialysis (first-year mortality) in your country?

- ◯ 0% (None)
- ◯ 1-10% (Few)
- ◯ 11-50% (Some)
- ◯ 51-75% (Most)
- ◯ >75% (Almost all)
- ◯ unknown

C13.2. What is the commonest cause of death among hemodialysis patents in your country?

- ◯ Cardiovascular disease (ischemic heart disease, arrythmia, cerebrovascular disease)
- ◯ Infection (access-related infection, infected AVF/AVG, catheter-related bacteraemia)
- ◯ Infection (other sources, pneumonia, gangrene of limbs, etc.)
- ◯ Malignancy
- ◯ Dialysis withdrawal (due to social reasons)
- ◯ Dialysis withdrawal (due to cost of care)
- ◯ Others (please specify)
- ◯ unknown

C13.2. If other, please specify.

____

C13.3. What proportion (national average) of patients with kidney failure (ESKD) on hemodialysis requires at least one hospitalization in the first year of dialysis (first-year hospitalization) in your country?

- ◯ 1-10%
- ◯ 11-20%
- ◯ 21-30%
- ◯ 31-50%
- ◯ >50%
- ◯ unknown

C13.4. What is commonest cause of hospitalization among hemodialysis patients in your country?

- ◯ Cardiovascular disease (ischemic heart disease, arrythmia, cerebrovascular disease)
- ◯ Access malfunction (malfunction AVF/AVG, or blocked central venous catheter)
- ◯ Access-related infection (infected AVF/AVG, CVC catheter-related bacteraemia)
- ◯ Infection (other sources, pneumonia, gangrene of limbs, etc.)
- ◯ Others (please specify)
- ◯ unknown

C13.4. If other, please specify.

____

C14. Outcomes (peritoneal dialysis)

C14.1. What proportion (national average) of patients with kidney failure (ESKD) on peritoneal dialysis died in the first year of dialysis (first-year mortality) in your country?	◯ 1-10%<br>◯ 11-20%<br>◯ 21-30%<br>◯ 31-50%<br>◯ >50%<br>◯ unknown
C14.2. What is the commonest cause of death among peritoneal dialysis patents in your country?	◯ Cardiovascular disease (ischemic heart disease, arrythmia, cerebrovascular disease)<br>◯ PD-related Infection (PD-related peritonitis, exit-site, or tunnel tract infection)<br>◯ Infection (other sources, pneumonia, gangrene of limbs, etc.)<br>◯ Malignancy<br>◯ Dialysis withdrawal (due to social reasons)<br>◯ Dialysis withdrawal (due to cost of care)<br>◯ Others (please specify)<br>◯ unknown
C14.2. If other, please specify.	______________________________
C14.3 What proportion (national average) of patients with kidney failure (ESKD) on peritoneal dialysis require at least one hospitalization in the first year of dialysis (first-year hospitalization) in your country?	◯ 1-10%<br>◯ 11-20%<br>◯ 21-30%<br>◯ 31-50%<br>◯ >50%<br>◯ unknown
C14.4. What is commonest cause of hospitalization among peritoneal dialysis patients in your country?	◯ Cardiovascular disease (ischemic heart disease, arrythmia, cerebrovascular disease)<br>◯ Access malfunction (PD catheter block, catheter tip migration)<br>◯ PD-related infection (peritonitis, exit-site or tunnel tract infection)<br>◯ Infection (other sources, pneumonia, gangrene of limbs, etc.)<br>◯ Others (please specify)<br>◯ unknown
C14.4. If other, please specify.	______________________________

C15. Demographics

C15.1. What proportion (national average) of patients with kidney failure (ESKD) (kidney failure) are aged above 65 years in your country?

- ◯ 0%
- ◯ 1-10%
- ◯ 11-25%
- ◯ 26-50%
- ◯ >50%
- ◯ unknown

C15.2. What proportion (national average) of patients with kidney failure (ESKD) (kidney failure) are female in your country?

- ◯ 0%
- ◯ 1-10%
- ◯ 11-25%
- ◯ 26-50%
- ◯ >50%
- ◯ unknown

C16. Aetiology of kidney failure (ESKD)

C16.1. What is commonest cause of kidney failure (ESKD) in your country?

- ◯ Diabetes kidney disease
- ◯ Polycystic kidney disease
- ◯ Hypertension
- ◯ Glomerulonephritis
- ◯ Others (please specify)
- ◯ unknown

C16.1. If other, please specify.

____

C16.2. What proportion (national average) of patients with kidney failure (ESKD) are due to diabetes kidney disease in your country?

- ◯ 0%
- ◯ 1-25%
- ◯ 26-50%
- ◯ 51-75%
- ◯ >75%
- ◯ unknown

C16.3. What proportion (national average) of patients with kidney failure (ESKD) (kidney failure) are due to glomerulonephritis in your country?

- ◯ 0%
- ◯ 1-25%
- ◯ 26-50%
- ◯ 51-75%
- ◯ >75%
- ◯ unknown

C16.4. What proportion (national average) of patients with kidney failure (ESKD) (kidney failure) are due to polycystic kidney disease in your country?

- ◯ 0%
- ◯ 1-25%
- ◯ 26-50%
- ◯ 51-75%
- ◯ >75%
- ◯ unknown

C16.5 What proportion (national average) of patients with kidney failure (ESKD) are due to hypertensive kidney disease in your country?

- ◯ 0%
- ◯ 1-25%
- ◯ 26-50%
- ◯ 51-75%
- ◯ >75%
- ◯ unknown

C17 Use of Technology in Kidney Care

C17.1. Is there capacity for telehealth / telenephrology reviews for management of CKD and kidney failure (ESKD) in your country?	◯ Yes (if possible, please provide brief details)<br>◯ No<br>◯ Unknown
C17.1. If yes, please provide details.	__________________________________
C17.2. Do patients have the option to receive communication (i.e. test results, appointment reminders) from clinics / hospitals via text message or email?	◯ Yes (if possible, please provide brief details)<br>◯ No<br>◯ Unknown
C17.2. If yes, please provide details.	__________________________________
C17.3. Is there funding / reimbursement for providers who provide telehealth / telenephrology remote reviews for CKD and kidney failure (ESKD)?	◯ Publicly funded by government and free at the point of delivery<br>◯ Publicly funded by government but with some fees at the point of delivery<br>◯ A mix of publicly funded (whether or not publicly funded component is free at point of delivery) and private systems (please explain)<br>◯ Solely private and out-of-pocket<br>◯ Solely private through health insurance providers<br>◯ Multiple systems - programs provided by government, NGOs, and communities<br>◯ N/A (telehealth / telenephrology is not available in my country)<br>◯ Other (please specify)
C17.3. If other, please specify.	__________________________________
C17.3. If a mix of public and private funding, please explain.	__________________________________
C18.1. Are there guidelines regarding measures that should be taken for disaster preparedness (i.e. at dialysis facilities) in the event of an earthquake / flood / drought in your country?	◯ Yes (if possible, please provide brief details)<br>◯ No<br>◯ Unknown
C18.1. If yes, please provide details.	__________________________________
C18.2. Does your country have a representative in the Renal Disaster Relief Task Force?	◯ Yes (if possible, please provide brief details)<br>◯ No<br>◯ Unknown
C18.2. If yes, please provide details.	__________________________________

C18.3. Are there means of identifying vulnerable populations (i.e. people with housing insecurity, racial/ethnic minorities, people living in poverty, people with food insecurity) in your country	◯ Yes (if possible, please provide brief details)<br>◯ No<br>◯ Unknown
C18.3. If yes, please provide details.	__________________________________

C18.4. What best describes your healthcare system funding structure for treatment of CKD and kidney failure (ESKD) in refugee populations?

Reduce font size if the text below is overlapping.

	Publicly funded by government and free at the point of delivery	Publicly funded by government but with some fees at the point of delivery	A mix of publicly funded (whether or not publicly funded component is free at point of delivery) and private systems (please explain)	Solely private and out-of-pocket	Solely private through health insurance providers	Multiple systems - programs provided by government, NGOs, and communities	Other (please specify)	N/A (refugees do not routinely have access to treatment for kidney disease)
C18.4.1. Hemodialysis (some or all aspects of)	○	○	○	○	○	○	○	○

C18.4. What best describes your healthcare system funding structure for treatment of CKD and kidney failure (ESKD) in refugee populations?

Reduce font size if the text below is overlapping.

C18.4.2. Peritoneal dialysis (some or all aspects of)	○	○	○	○	○	○	○	○

C18.4. What best describes your healthcare system funding structure for treatment of CKD and kidney failure (ESKD) in refugee populations?

Reduce font size if the text below is overlapping.

C18.4.3. Kidney transplantation (some or all aspects of)	○	○	○	○	○	○	○	○

C18.4. What best describes your healthcare system funding structure for treatment of CKD and kidney failure (ESKD) in refugee populations?

Reduce font size if the text below is overlapping.

C18.4.4. Conservative care (some or all aspects of) ○ ○ ○ ○ ○ ○ ○ ○

C18.4.1. If other, please specify. __________________________________

C18.4.1. If a mix of public and private funding, please explain. __________________________________

C18.4.2. If other, please specify. __________________________________

C18.4.2. If a mix of public and private funding, please explain. __________________________________

C18.4.3. If other, please specify. __________________________________

C18.4.3. If a mix of public and private funding, please explain. __________________________________

C18.4.4. If other, please specify. __________________________________

C18.4.4. If a mix of public and private funding, please explain. __________________________________

D. Health information systems and statistics
D1. Registries

Definitions/abbreviations

Registry: A systematic collection of data to evaluate specified outcomes for a defined population in order to serve one or more predetermined scientific, clinical, or policy purposes.

D1.1. For which conditions or treatments is there an 'official' registry in your country?

	Yes	No	Unknown
D1.1.1. CKD (non-KRT)	◯	◯	◯

D. Health information systems and statistics
D1. Registries

Definitions/abbreviations

Registry: A systematic collection of data to evaluate specified outcomes for a defined population in order to serve one or more predetermined scientific, clinical, or policy purposes.

D1.1. For which conditions or treatments is there an 'official' registry in your country?

D1.1.2. Dialysis	○	○	○

D. Health information systems and statistics
D1. Registries

Definitions/abbreviations

Registry: A systematic collection of data to evaluate specified outcomes for a defined population in order to serve one or more predetermined scientific, clinical, or policy purposes.

D1.1. For which conditions or treatments is there an 'official' registry in your country?

D1.1.3. Transplantation	○	○	○

D. Health information systems and statistics
D1. Registries

Definitions/abbreviations

Registry: A systematic collection of data to evaluate specified outcomes for a defined population in order to serve one or more predetermined scientific, clinical, or policy purposes.

D1.1. For which conditions or treatments is there an 'official' registry in your country?

D1.1.4. AKI ◯ ◯ ◯

D. Health information systems and statistics
D1. Registries

Definitions/abbreviations

Registry: A systematic collection of data to evaluate specified outcomes for a defined population in order to serve one or more predetermined scientific, clinical, or policy purposes.

D1.1. For which conditions or treatments is there an 'official' registry in your country?

D1.1.5. Conservative care ◯ ◯ ◯

D1.2. If there is a CKD registry for patients who do not require KRT, what is the basis of participation in the CKD registry?

◯ Voluntary
◯ Mandatory
◯ Unknown

D1.3. If there is a CKD registry for patients who do not require KRT, what is the geographical coverage of the CKD registry? (please check all that apply)

☐ National
☐ Regional/state/provincial
☐ Local/hospital/community

D1.4. If there is a CKD registry for patients who do not require KRT, what does it cover?

◯ The whole spectrum of CKD (stages 1-5)
◯ Advanced CKD only (stages 4/5)

D1.5. If there is a dialysis registry, what is the basis of participation in the dialysis registry?

◯ Voluntary
◯ Mandatory
◯ Unknown

D1.6. If there is a dialysis registry, what is the geographical coverage of the dialysis registry? (please check all that apply)

☐ National
☐ Regional/state/provincial
☐ Local/hospital/community

D1.7. If there is a dialysis registry, what information does the dialysis registry collate? (please check all that apply)

☐ Aetiology of kidney failure (ESKD)
☐ Modality of dialysis
☐ Dialysis prescription
☐ Dialysis access (e.g., vascular access for HD, PD catheter)
☐ Process-based measures (e.g., anaemia, bone disease, BP control markers)
☐ Patient outcome measures (e.g., hospitalizations)
☐ Patient outcome measures (e.g., satisfaction, quality of life)
☐ Patient outcome measures (e.g., mortality)

D1.8. If there is a transplantation registry, what is the basis of participation in the transplant registry?

◯ Voluntary
◯ Mandatory
◯ Unknown

D1.9. If there is a transplantation registry, what is the geographical coverage of the transplant registry? (please check all that apply)

☐ National
☐ Regional/state/provincial
☐ Local/hospital/community

D1.10. If there is a transplantation registry, what information does the transplant registry collate? (please check all that apply)	☐ Aetiology of kidney failure (ESKD)<br>☐ Transplant source (deceased/live donor)<br>☐ Type of immunosuppression<br>☐ Episodes of rejection<br>☐ Types and episodes of infection<br>☐ Patient outcome measures (e.g., hospitalizations)<br>☐ Patient outcome measures (e.g., satisfaction, quality of life)<br>☐ Patient outcome measures (e.g., mortality)
D1.11. If there is an AKI registry, what is the basis of participation in the AKI registry?	○ Voluntary<br>○ Mandatory<br>○ Unknown
D1.12. If there is an AKI registry, what is the geographical coverage of the AKI registry? (please check all that apply)	☐ National<br>☐ Regional/state/provincial<br>☐ Local/hospital/community
D1.13. If there is an AKI registry, what does it cover? (please check all that apply)	○ The whole spectrum of AKI (stages 1-3)<br>○ AKI requiring kidney replacement therapy
D1.14. If there is an AKI registry, what information does the AKI registry collate? (please check all that apply)	☐ Risk factors for AKI<br>☐ Aetiology of AKI<br>☐ Incidence of AKI<br>☐ Patient outcome measures (hospitalizations)<br>☐ Patient outcome measures (requirement for KRT, for example, dialysis or slow dialysis therapies like CKRT)<br>☐ Patient outcome measures (mortality)

D2. Identification of disease (AKI and CKD)
Definitions:
Guidelines: Evidence-based recommended courses of action for prevention or management of disease.
Identification: Measures performed in at-risk populations in order to diagnose individuals who have risk factors or early stages of disease but may not yet have symptoms.
Policy: A specific official decision or set of decisions designed to carry out a course of action endorsed by a government body; including a set of goals, priorities and main directions for attaining these goals. The policy document may include a strategy to give effect to the policy.
Program: A planned set of activities or procedures directed at a specific purpose.

D2.1. For which of the following high-risk groups do practitioners in your country routinely offer testing for CKD? (please check all that apply)

- ☐ Those with hypertension
- ☐ Those with diabetes
- ☐ Those with cardiovascular disease (ischemic heart disease, stroke, peripheral vascular disease, heart failure)
- ☐ Those with autoimmune/multisystem diseases (systemic lupus erythematosus, rheumatoid arthritis)
- ☐ The elderly
- ☐ Those with urological disorders (structural, stone diseases)
- ☐ Chronic users of nephrotoxic medications
- ☐ Members of high-risk ethnic groups (Aboriginal, African, Indo-Asian)
- ☐ Those with a family history of CKD
- ☐ N/A - routine testing for CKD not offered

D2.2. In your country, are there ethnic groups considered to be at increased risk for CKD?

- ○ Yes (please specify below)
- ○ No
- ○ Unknown

D2.2. If yes, please specify.

____

D2.3. In your country, is a CKD detection program in use that is based on national policy or guidelines?

- ○ Yes
- ○ No
- ○ Unknown

D2.3.1. If there is a program, how is it implemented?

- ○ Reactive approach - cases managed as identified through practice
- ○ Active screening of at-risk population through routine health encounters
- ○ Active screening of at-risk population through specific screening processes
- ○ Other (please specify)

D2.3.1 If other, please specify.

____

D2.4. In your country, are there specific groups considered to be at increased risk for AKI?

- ○ Yes (please specify below)
- ○ No
- ○ Unknown

D2.4. If yes, please specify.	__________________________________
D2.5. In your country, is an AKI detection program in use that is based on national policy and/or guidelines?	◯ Yes<br>◯ No<br>◯ Unknown
D2.5.1. If there is an AKI detection program, how is it implemented?	◯ Reactive approach - cases managed as identified through practice<br>◯ Active screening of at-risk population through routine health encounters<br>◯ Active screening of at-risk population through specific screening processes<br>◯ Automated computation by pathology systems with electronic alerts<br>◯ Other (please specify)
D2.5.1. If other, please specify.	__________________________________
D3. Do you have mechanisms to ensure the validity and quality of data contained within health information systems?	◯ Yes<br>◯ No<br>◯ Unknown

D4. Capacity for identification and management of CKD

D4.1. Indicate the availability of the following services for CKD monitoring and management at PRIMARY care level in your country

	Available	Not Available
D4.1.1. Blood pressure measurement	○	○

D4. Capacity for identification and management of CKD

D4.1. Indicate the availability of the following services for CKD monitoring and management at PRIMARY care level in your country

D4.1.2. Height and weight measures to calculate body mass index	○	○

D4. Capacity for identification and management of CKD

D4.1. Indicate the availability of the following services for CKD monitoring and management at PRIMARY care level in your country

D4.1.3. Serum glucose measurement	○	○

D4. Capacity for identification and management of CKD

D4.1. Indicate the availability of the following services for CKD monitoring and management at PRIMARY care level in your country

D4.1.4. HbA1C test ○ ○

D4. Capacity for identification and management of CKD

D4.1. Indicate the availability of the following services for CKD monitoring and management at PRIMARY care level in your country

D4.1.5. Serum cholesterol measurement	○	○

D4. Capacity for identification and management of CKD

D4.1. Indicate the availability of the following services for CKD monitoring and management at PRIMARY care level in your country

D4.1.6. Serum creatinine measurement without automated eGFR reporting	○	○

D4. Capacity for identification and management of CKD

D4.1. Indicate the availability of the following services for CKD monitoring and management at PRIMARY care level in your country

D4.1.7. Serum creatinine measurement with automated eGFR reporting	○	○

D4. Capacity for identification and management of CKD

D4.1. Indicate the availability of the following services for CKD monitoring and management at PRIMARY care level in your country

D4.1.8. Urinalysis using test strips for albumin/protein (qualitative assays)	○	○

D4. Capacity for identification and management of CKD

D4.1. Indicate the availability of the following services for CKD monitoring and management at PRIMARY care level in your country

D4.1.9. Urinalysis using test strips for albumin/protein (quantitative assays)	○	○

D4. Capacity for identification and management of CKD

D4.1. Indicate the availability of the following services for CKD monitoring and management at PRIMARY care level in your country

D4.1.10. Urine albumin: creatinine ratio (ACR) or protein: creatinine (PCR) measurements	○	○

D4.2. Indicate the availability of the following services for CKD monitoring and management at SECONDARY OR TERTIARY care level in your country.

	Available	Not Available
D4.2.1. Blood pressure measurement	○	○

D4.2. Indicate the availability of the following services for CKD monitoring and management at SECONDARY OR TERTIARY care level in your country.

D4.2.2. Height and weight measures to calculate body mass index	○	○

D4.2. Indicate the availability of the following services for CKD monitoring and management at SECONDARY OR TERTIARY care level in your country.

D4.2.3. Serum glucose measurement	○	○

D4.2. Indicate the availability of the following services for CKD monitoring and management at SECONDARY OR TERTIARY care level in your country.

D4.2.4. HbA1C test ◯ ◯

D4.2. Indicate the availability of the following services for CKD monitoring and management at SECONDARY OR TERTIARY care level in your country.

D4.2.5. Serum cholesterol measurement	○	○

D4.2. Indicate the availability of the following services for CKD monitoring and management at SECONDARY OR TERTIARY care level in your country.

D4.2.6. Serum creatinine measurement without automated eGFR reporting	○	○

D4.2. Indicate the availability of the following services for CKD monitoring and management at SECONDARY OR TERTIARY care level in your country.

D4.2.7. Serum creatinine measurement with automated eGFR reporting	○	○

D4.2. Indicate the availability of the following services for CKD monitoring and management at SECONDARY OR TERTIARY care level in your country.

D4.2.8. Urinalysis using test strips for albumin/protein (qualitative assays)	○	○

D4.2. Indicate the availability of the following services for CKD monitoring and management at SECONDARY OR TERTIARY care level in your country.

D4.2.9. Urinalysis using test strips for albumin/protein (quantitative assays)	○	○

D4.2. Indicate the availability of the following services for CKD monitoring and management at SECONDARY OR TERTIARY care level in your country.

D4.2.10. Urine albumin: creatinine ratio (ACR) or protein: creatinine (PCR) measurements	○	○

D4.2. Indicate the availability of the following services for CKD monitoring and management at SECONDARY OR TERTIARY care level in your country.

D4.2.11. Radiological services (e.g. facilities for kidney ultrasound)	○	○

D4.2. Indicate the availability of the following services for CKD monitoring and management at SECONDARY OR TERTIARY care level in your country.

D4.2.12. Pathology services (kidney biopsy interpretation facilities)	○	○

D5: CKD of unknown origin and populations disproportionately affected with CKD

This is a section to understand whether regional hotspots of kidney disease exist (specifically, CKD of unknown origin) in your country.

D.5.1. Do you suspect that there are regional variations in the rate of kidney disease in your country? (By this we mean population clusters with high risk of kidney failure requiring dialysis or transplant, or people dying of kidney failure?)

◯ Yes (please specify)
◯ No

D5.1. If yes, please specify.

____

D.5.2. Age groups affected (select all that apply):

☐ < 18 years of age
☐ 18-44 years of age
☐ 45-64 years of age
☐ 65+ years

D.5.3 In your opinion what are some of the likely causes or contributors to kidney disease in these areas (select all that apply):

☐ Diabetes
☐ Obesity
☐ High blood pressure
☐ Environmental (e.g., water, soil)
☐ Climate (e.g., high temperature)
☐ Genetic
☐ Biological (e.g., high HIV or TB prevalence, stones)
☐ Cultural (e.g., diet, use of NSAIDs, herbs)
☐ Other (please specify)

D.5.3 If other, please specify.

____

D.5.4. Types of industries in this region / these regions (select all that apply):

☐ Agriculture
☐ Manufacturing
☐ Mining
☐ Tourism
☐ Service/Professional
☐ Other (please specify)

D.5.4. If other, please specify.

____

D.5.5. Predominant type of climate in the country (select only one):

◯ Tropical
◯ Semi-arid
◯ Temperate
◯ Other (please specify)

D.5.5. If other, please specify.

____

D.5.6. What is the altitude of the country?	◯ High<br>◯ At Sea-level<br>◯ Low<br>◯ Other (please provide details)
D.5.6. If other, please provide details.	__________________________________

E. National health policy

Definitions:

Policy: A specific official decision or set of decisions designed to carry out a course of action endorsed by a government body; including a set of goals, priorities and main directions for attaining these goals. The policy document may include a strategy to give effect to the policy.

Program: A planned set of activities or procedures directed at a specific purpose.

Strategy: a long-term plan designed to achieve a particular goal.

E1. Policy and strategy

Non-communicable diseases (NCDs): Diseases that cannot be transmitted from person to person, notably cardiovascular diseases (like heart attacks and stroke), cancers, chronic respiratory diseases (such as chronic obstructive pulmonary disease and asthma) and diabetes.

E1.1. Does your country have a national strategy for non-communicable diseases?	◯ Yes, in place (please provide details below)<br>◯ Under development but not yet being implemented (please provide details below)<br>◯ No<br>◯ Unknown
E1.1. If yes, please provide details.	__________________________________
E1.1. If under development, please provide details.	__________________________________
E1.2. Does your country have a national strategy for improving the care of CKD patients?	◯ Yes, a national CKD-specific strategy exists<br>◯ Yes, but the CKD strategy is incorporated into an NCD strategy that includes other diseases.<br>◯ No<br>◯ Unknown
E1.2.1. Please select which populations are covered in the national CKD-specific strategy (check all that apply	☐ Non-dialysis dependent CKD<br>☐ Chronic dialysis<br>☐ Kidney transplantation
E1.2.2. Please select which populations are covered in the national general NCD strategy (check all that apply)	☐ Non-dialysis dependent CKD<br>☐ Chronic dialysis<br>☐ Kidney transplantation
E1.3. Are CKD-specific policies available?	◯ Yes<br>◯ No<br>◯ Unknown
E1.3.1. If yes, please specify which type of CKD policies are available in your country (check all that apply)	☐ National policies<br>☐ Regional policies

E2. Advocacy

E2.1. In your opinion, is CKD recognized as a health priority by the government in your country?

◯ Yes (please provide details below)
◯ No (please explain why not below)

E2.1. If yes, please provide details.

____

E2.1. If no, please explain.

____

E2.2. Is there an advocacy group at the higher levels of government (for example, a parliamentary committee) or an NGO to raise the profile of CKD and its prevention?

◯ Yes (please provide details below)
◯ No (please explain why not below)
◯ Unknown

E2.2. If yes, please provide details.

____

E2.2. If no, please explain.

____

E2.3. In your opinion, is AKI and/or its prevention recognized as a health priority by the government in your country?

◯ Yes (please provide details below)
◯ No (please explain why not below)

E2.3. If yes, please provide details.

____

E2.3. If no, please explain.

____

E2.4. s there an advocacy group at the higher levels of government (for example, a parliamentary committee) or an NGO to raise the profile of AKI and its prevention?

◯ Yes (please provide details below)
◯ No (please explain why not below)
◯ Unknown

E2.4. If yes, please provide details.

____

E2.4. If no, please explain.

____

E2.5. In your opinion, is kidney failure (ESKD) (kidney failure) and/or its treatment by KRT recognized as a health priority by the government in your country?

◯ Yes (please provide details below)
◯ No (please explain why not below)

E2.5. If yes, please provide details.

____

E2.5. If no, please explain.

____

E2.6. Is there an advocacy group at the higher levels of government (for example, a parliamentary committee) or an NGO to raise the profile of kidney failure (ESKD)/KRT?

◯ Yes (please provide details below)
◯ No (please explain why not below)
◯ Unknown

E2.6. If yes, please provide details.

____

E2.6. If no, please explain.

____

E2.7. Are there existing national/regional physician-oriented organizations or patient organizations that provide resources for kidney failure (ESKD) (kidney failure) care?

◯ Yes (please provide details below)
◯ No (please explain why not below)
◯ Unknown

E2.7. If yes, please provide details.

____

E2.7. If no, please explain.

____

E3.1. Are there specific barriers to optimal kidney care in your country? Please check all that apply

☐ Geography (distance from care or prolonged travel time)
☐ Physician (availability, access, knowledge, attitude)
☐ Patient (knowledge, attitude)
☐ Nephrologist (availability)
☐ Healthcare system (availability, access, capability)
☐ Lack of political will and enabling policies
☐ Economic factors (limited funding, poor reimbursement mechanisms)
☐ Other (please specify)

E3.1. If other, please specify.

____

E4. How did you gather the information to complete this questionnaire? Please check all that apply.

☐ Personal opinion/knowledge
☐ Gathered knowledge from other sources (for example, published literature or reports)
☐ Consultation with other colleagues
☐ Other (please specify)

E4. If other, please specify.

____

ISN-Enquête ISN Global Kidney Health Atlas (ISN GKHA)

Please complete the survey below.

Thank you!

Enquête ISN-Global Kidney Health Atlas (ISN-GKHA)

Enquête thématique :

Évaluation du statut mondial des soins de santé rénale : capacité, disponibilité, accessibilité, abordabilité financière et résultats

Profils nationaux de l'évaluation du statut mondial des soins de santé rénale : capacité, disponibilité, accessibilité, abordabilité financière et résultats

L'International Society of Nephrology (ISN) travaille en collaboration avec des organisations et des initiatives aux niveaux international et national, afin de promouvoir la détection précoce et le traitement efficace des maladies rénales et d'améliorer ainsi la santé et la qualité de vie des patients. À travers la compréhension et la contribution éventuelle à la mise au point de politiques, pratiques et infrastructures de santé pertinentes, l'ISN vise à faciliter la mise en œuvre de soins équitables et éthiques pour les patients atteints d'une maladie rénale, dans toutes les régions et tous les pays du monde.

Les Global Kidney Health Atlas 2017 (1re édition)) et 2019 (2e édition) de l'ISN présentent les résultats de l'étude sur le statut des soins des patients atteints d'une maladie rénale dans tous les pays du monde ;).

L'ISN-GKHA a mis en évidence une variabilité inter et intrarégionale importante dans le domaine des soins rénaux à l'échelle mondiale, avec des lacunes significatives en termes de personnel de santé rénale, de prestation de services de santé, de médicaments et technologies essentiels, de financement de la santé, de leadership et de gouvernance, de systèmes d'information de santé, de stratégies et de cadres politiques, ainsi que de capacité de recherche et de développement, en particulier dans les pays à revenu faible et intermédiaire. En identifiant les lacunes dans les domaines des soins de santé universels, ces résultats ont fourni une plate-forme de sensibilisation à la maladie rénale chronique (MRC) et une base pour un réseau mondial de surveillance et de benchmarking de la MRC.

La troisième édition de l'enquête de l'ISN vise à comprendre, comparer et surveiller comment différents pays dans le monde détectent, traitent, surveillent et défendent les personnes atteintes d'une maladie rénale, en mettant l'accent sur la capacité, la disponibilité, l'accessibilité, l'abordabilité financière et les résultats.

Elle tentera de déterminer la capacité et l'état de préparation des nations à permettre un accès universel à des soins rénaux intégrés équitables (y compris le traitement de suppléance rénale et les soins conservateurs). Cette édition comprend également une enquête auprès des patients, qui permet de connaître le point de vue de ceux-ci sur l'accès et la qualité des soins qui leur sont dispensés.

L'enquête actuelle porte sur les principaux domaines qui fournissent des informations sur les aspects de la couverture sanitaire universelle spécifiques aux soins rénaux intégrés : financement de la santé, main-d'œuvre, accès aux médicaments et produits de santé essentiels, systèmes d'information et statistiques relatifs à la santé, politiques, prestation de services et sécurité, ainsi que sur la réponse de la communauté néphrologique et la capacité de recherche et de développement dans le domaine des soins rénaux.

Après consultation des dix comités régionaux de l'ISN, l'enquête originale rédigée en anglais a été traduite en français et en espagnol, ces langues étant représentatives de celles parlées et comprises par la majorité des répondants à cette enquête.

Si vous avez des questions concernant la participation à l'enquête, veuillez contacter : Sandrine Damster (e-mail : GlobalAtlas@theisn.org).

Nous vous remercions pour votre implication et votre disposition à participer à cette enquête.

Professeur Agnès Fogo

Présidente, International Society of Nephrology (ISN)

Liste des abréviations :

LRA : lésion rénale aiguë Fistule AV : fistule artério-veineuse
DPA : dialyse péritonéale automatisée
PA : pression artérielle MRC : maladie rénale chronique
GRC : gestion rénale conservatrice
MRST : maladie rénale au stade terminal TSR : traitement de suppléance rénale (hémodialyse, dialyse péritonéale, transplantation rénale)
MNT : maladie non transmissible ONG : organisation non gouvernementale
HD : hémodialyse
DP : dialyse péritonéale
HPT : hormone parathyroïdienne

1. Identifiant de l'enquête (communiqué par e-mail) : ______________________

2. Fonction actuelle :	__________________________________
3. Rôle : Veuillez cocher toutes les réponses qui s'appliquent.	◯ Néphrologue<br>◯ Néphrologue pédiatrique<br>◯ Médecin autre que néphrologue<br>◯ Professionnel de santé (non-médecin) (préciser)<br>◯ Administrateur/décideur politique/fonctionnaire<br>◯ Autre (veuillez préciser)
3. Si autre, veuillez préciser	____

4. Dans quel pays résidez-vous ?

- ◯ Afghanistan
- ◯ Åland Islands
- ◯ Albania
- ◯ Algeria
- ◯ American Samoa
- ◯ Andorra
- ◯ Angola
- ◯ Anguilla
- ◯ Antarctica
- ◯ Antigua And Barbuda
- ◯ Argentina
- ◯ Armenia
- ◯ Aruba
- ◯ Australia
- ◯ Austria
- ◯ Azerbaijan
- ◯ Bahamas
- ◯ Bahrain
- ◯ Bangladesh
- ◯ Barbados
- ◯ Belarus
- ◯ Belgium
- ◯ Belize
- ◯ Benin
- ◯ Bermuda
- ◯ Bhutan
- ◯ Bolivia
- ◯ Bosnia And Herzegovina
- ◯ Botswana
- ◯ Bouvet Island
- ◯ Brazil
- ◯ British Indian Ocean Territory
- ◯ Brunei Darussalam
- ◯ Bulgaria
- ◯ Burkina Faso
- ◯ Burundi
- ◯ Cambodia
- ◯ Cameroon
- ◯ Canada
- ◯ Cape Verde
- ◯ Cayman Islands
- ◯ Central African Republic
- ◯ Chad
- ◯ Chile
- ◯ China
- ◯ Christmas Island
- ◯ Cocos (Keeling) Islands
- ◯ Colombia
- ◯ Comoros
- ◯ Congo
- ◯ Congo (The Democratic Republic Of The)
- ◯ Cook Islands
- ◯ Costa Rica
- ◯ Cote D'ivoire
- ◯ Croatia
- ◯ Cuba
- ◯ Cyprus
- ◯ Czechia
- ◯ Denmark
- ◯ Djibouti
- ◯ Dominica
- ◯ Dominican Republic
- ◯ Ecuador
- ◯ Egypt
- ◯ El Salvador
- ◯ Equatorial Guinea
- ◯ Eritrea
- ◯ Estonia
- ◯ Ethiopia

◯ Falkland Islands (Malvinas)
◯ Faroe Islands
◯ Fiji
◯ Finland
◯ France
◯ French Guiana
◯ French Polynesia
◯ French Southern Territories
◯ Gabon
◯ Gambia
◯ Georgia
◯ Germany
◯ Ghana
◯ Gibraltar
◯ Greece
◯ Greenland
◯ Grenada
◯ Guadeloupe
◯ Guam
◯ Guatemala
◯ Guernsey
◯ Guinea
◯ Guinea-bissau
◯ Guyana
◯ Haiti
◯ Heard Island And Mcdonald Islands
◯ Holy See (Vatican City State)
◯ Honduras
◯ Hong Kong
◯ Hungary
◯ Iceland
◯ India
◯ Indonesia
◯ Iran, Islamic Republic Of
◯ Iraq
◯ Ireland
◯ Isle Of Man
◯ Israel
◯ Italy
◯ Jamaica
◯ Japan
◯ Jersey
◯ Jordan
◯ Kazakhstan
◯ Kenya
◯ Kiribati
◯ Korea: Democratic People's Republic Of
◯ Korea: Republic Of
◯ Kosovo
◯ Kuwait
◯ Kyrgyzstan
◯ Lao People's Democratic Republic
◯ Latvia
◯ Lebanon
◯ Lesotho
◯ Liberia
◯ Libyan Arab Jamahiriya
◯ Liechtenstein
◯ Lithuania
◯ Luxembourg
◯ Macao
◯ Macedonia
◯ Madagascar
◯ Malawi
◯ Malaysia
◯ Maldives
◯ Mali
◯ Malta
◯ Marshall Islands
◯ Martinique
◯ Mauritania

◯ Mauritius
◯ Mayotte
◯ Mexico
◯ Micronesia
◯ Moldova, Republic Of
◯ Monaco
◯ Mongolia
◯ Montenegro
◯ Montserrat
◯ Morocco
◯ Mozambique
◯ Myanmar
◯ Namibia
◯ Nauru
◯ Nepal
◯ Netherlands
◯ Netherlands Antilles
◯ New Caledonia
◯ New Zealand
◯ Nicaragua
◯ Niger
◯ Nigeria
◯ Niue
◯ Norfolk Island
◯ Northern Mariana Islands
◯ Norway
◯ Oman
◯ Pakistan
◯ Palau
◯ Palestinian Territory, Occupied
◯ Panama
◯ Papua New Guinea
◯ Paraguay
◯ Peru
◯ Philippines
◯ Pitcairn
◯ Poland
◯ Portugal
◯ Puerto Rico
◯ Qatar
◯ Reunion
◯ Romania
◯ Russian Federation
◯ Rwanda
◯ Saint Helena
◯ Saint Kitts And Nevis
◯ Saint Lucia
◯ Saint Pierre And Miquelon
◯ Saint Vincent And The Grenadines
◯ Samoa
◯ San Marino
◯ Sao Tome And Principe
◯ Saudi Arabia
◯ Senegal
◯ Serbia
◯ Seychelles
◯ Sierra Leone
◯ Singapore
◯ Slovakia
◯ Slovenia
◯ Solomon Islands
◯ Somalia
◯ South Africa
◯ South Georgia And The South Sandwich Islands
◯ South Sudan
◯ Spain
◯ Sri Lanka
◯ Sudan
◯ Suriname
◯ Svalbard And Jan Mayen
◯ Swaziland

◯ Sweden
◯ Switzerland
◯ Syrian Arab Republic
◯ Taiwan
◯ Tajikistan
◯ Tanzania, United Republic Of
◯ Thailand
◯ Timor-leste
◯ Togo
◯ Tokelau
◯ Tonga
◯ Trinidad And Tobago
◯ Tunisia
◯ Turkey
◯ Turkmenistan
◯ Turks And Caicos Islands
◯ Tuvalu
◯ Uganda
◯ Ukraine
◯ United Arab Emirates
◯ United Kingdom
◯ United States
◯ United States Minor Outlying Islands
◯ Uruguay
◯ Uzbekistan
◯ Vanuatu
◯ Venezuela
◯ Viet Nam
◯ Virgin Islands, British
◯ Virgin Islands, U.S.
◯ Wallis And Futuna
◯ Western Sahara
◯ Yemen
◯ Zambia
◯ Zimbabwe

---

5. Dans quelle ville résidez-vous ?

____

A. Financement de la santé et prestation de services de santé

A1. Système de soins de santé et mécanisme de financement

A1.1. De manière générale, qu'est-ce qui décrit le mieux la structure de financement de votre système de soins de santé en ce qui concerne la MRC (maladie rénale chronique) sans dialyse ? (veuillez sélectionner la réponse la plus appropriée)

- ◯ Financement public par le gouvernement et gratuit au lieu d'administration
- ◯ Financement public par le gouvernement, mais certains frais au lieu d'administration
- ◯ Combinaison d'un système financé par des fonds publics (que la composante financée par des fonds publics soit gratuite ou pas au lieu d'administration) et d'un système privé (veuillez expliquer) Zone de texte
- ◯ Uniquement privé et payant
- ◯ Uniquement privé via les compagnies d'assurance maladie
- ◯ Différents systèmes - programmes fournis par le gouvernement, des Organisations Non Gouvernementales (ONGs) et des communautés
- ◯ Autre (veuillez préciser)

A1.1. Si autre, veuillez préciser

____

A1.1. S'il s'agit d'un mélange de fonds publics et privés, veuillez expliquer.

____

A1.2. De manière générale, qu'est-ce qui décrit le mieux la structure de financement de votre système de soins de santé en ce qui concerne le TSR (traitement de suppléance rénale) ? (veuillez sélectionner la réponse la plus appropriée)

Réduisez la taille de la police si le texte ci-dessous se chevauche.

	Financement public par le gouvernement et gratuit au lieu d'administration	Financement public par le gouvernement, mais certains frais au lieu d'administration	Combinaison d'un système financé par des fonds publics (que la composante financée par des fonds publics soit gratuite ou pas au lieu d'administration) et d'un système privé (veuillez préciser)	Uniquement privé et payant	Uniquement privé via les compagnies d'assurance maladie	Différents systèmes - programmes fournis par le gouvernement, des ONG et des communautés	Autre (veuillez préciser)	S.O. (cette modalité n'est pas disponible dans mon pays)
A1.2.1. Dialyse aiguë pour une LRA (hémodialyse ou dialyse péritonéale)	○	○	○	○	○	○	○	○

A1.2. De manière générale, qu'est-ce qui décrit le mieux la structure de financement de votre système de soins de santé en ce qui concerne le TSR (traitement de suppléance rénale) ? (veuillez sélectionner la réponse la plus appropriée)

Réduisez la taille de la police si le texte ci-dessous se chevauche.

A1.2.2. Hémodialyse chronique	○	○	○	○	○	○	○	○

A1.2. De manière générale, qu'est-ce qui décrit le mieux la structure de financement de votre système de soins de santé en ce qui concerne le TSR (traitement de suppléance rénale) ? (veuillez sélectionner la réponse la plus appropriée)

Réduisez la taille de la police si le texte ci-dessous se chevauche.

A1.2.3. Dialyse péritonéale chronique	○	○	○	○	○	○	○	○

A1.2. De manière générale, qu'est-ce qui décrit le mieux la structure de financement de votre système de soins de santé en ce qui concerne le TSR (traitement de suppléance rénale) ? (veuillez sélectionner la réponse la plus appropriée)

Réduisez la taille de la police si le texte ci-dessous se chevauche.

A1.2.4. Médicaments pour une transplantation rénale ○ ○ ○ ○ ○ ○ ○ ○

A1.2.1. Si autre, veuillez préciser

____

A1.2.1. S'il s'agit d'un mélange de fonds publics et privés, veuillez expliquer.

____

A1.2.2. Si autre, veuillez préciser

____

A1.2.2. S'il s'agit d'un mélange de fonds publics et privés, veuillez expliquer.

____

A1.2.3. Si autre, veuillez préciser

____

A1.2.3. S'il s'agit d'un mélange de fonds publics et privés, veuillez expliquer.

____

A1.2.4. Si autre, veuillez préciser

____

A1.2.4. S'il s'agit d'un mélange de fonds publics et privés, veuillez expliquer.

____

A1.3.1. Si le TSR est financé (en tout ou en partie) par des fonds publics, cette couverture est-elle universelle (ce qui signifie que tous les résidents de votre pays y sont admissibles) ?

- ○ Oui, tous les résidents (y compris les enfants) sont inclus dans la couverture
- ○ Non, tous les résidents ne sont pas inclus (veuillez préciser)

A1.3.1. Si non, veuillez préciser

____

A1.3.2. Si le TSR est financé (en tout ou en partie) par des fonds publics, les populations vulnérables (réfugiés, populations déplacées) y sont-elles admissibles ?

- ○ Oui, toutes les populations vulnérables sont incluses dans la couverture
- ○ Non, toutes les populations vulnérables ne sont pas incluses (veuillez fournir des détails)

A1.3.2. Si non, veuillez préciser

____

A1.3.3. Si le TSR est financé (en tout ou en partie) par des fonds publics, quels aspects des soins ne sont-ils pas inclus dans la couverture ? Veuillez cocher toutes les réponses qui s'appliquent.

- ☐ Dialyse
- ☐ Transplantation
- ☐ Soins conservateurs complets (services de soutien et palliatifs rénaux)
- ☐ Gestion des complications associées (anémie, maladie osseuse, malnutrition)
- ☐ Autre (veuillez préciser)

A1.3.3. Si autre, veuillez préciser

____

A1.4. Qu'est-ce qui décrit le mieux la couverture de votre système de soins de santé en ce qui concerne les services chirurgicaux dans le cadre du TSR ? (veuillez sélectionner la réponse la plus appropriée sur chaque ligne) (si le TSR n'est pas disponible dans votre pays, passez cette rubrique)

Réduisez la taille de la police si le texte ci-dessous se chevauche.

	Financement public par le gouvernement et gratuit au lieu d'administration	Financement public par le gouvernement, mais certains frais au lieu d'administration	Combinaison d'un système financé par des fonds publics (que la composante financée par des fonds publics soit gratuite ou pas au lieu d'administration) et d'un système privé (veuillez préciser)	Uniquement privé et payant	Uniquement privé via les compagnies d'assurance maladie	Différents systèmes - programmes fournis par le gouvernement, des ONG et des communautés	Autre (veuillez préciser)
A1.4.1. Accès vasculaire pour l'hémodialyse (cathéters veineux centraux)	○	○	○	○	○	○	○

A1.4. Qu'est-ce qui décrit le mieux la couverture de votre système de soins de santé en ce qui concerne les services chirurgicaux dans le cadre du TSR ? (veuillez sélectionner la réponse la plus appropriée sur chaque ligne) (si le TSR n'est pas disponible dans votre pays, passez cette rubrique)

Réduisez la taille de la police si le texte ci-dessous se chevauche.

A1.4.2. Accès vasculaire pour l'hémodialyse (création d'une fistule ou d'un greffon)	○	○	○	○	○	○	○

A1.4. Qu'est-ce qui décrit le mieux la couverture de votre système de soins de santé en ce qui concerne les services chirurgicaux dans le cadre du TSR ? (veuillez sélectionner la réponse la plus appropriée sur chaque ligne) (si le TSR n'est pas disponible dans votre pays, passez cette rubrique)

Réduisez la taille de la police si le texte ci-dessous se chevauche.

A1.4.3. Chirurgie d'accès pour la dialyse péritonéale (insertion d'un cathéter de DP)	○	○	○	○	○	○	○

A1.4. Qu'est-ce qui décrit le mieux la couverture de votre système de soins de santé en ce qui concerne les services chirurgicaux dans le cadre du TSR ? (veuillez sélectionner la réponse la plus appropriée sur chaque ligne) (si le TSR n'est pas disponible dans votre pays, passez cette rubrique)

Réduisez la taille de la police si le texte ci-dessous se chevauche.

A1.4.4. Chirurgie de transplantation rénale ○ ○ ○ ○ ○ ○ ○

A1.4.1. Si autre, veuillez préciser

____

A1.4.1. S'il s'agit d'un mélange de fonds publics et privés, veuillez expliquer.

____

A1.4.2. Si autre, veuillez préciser

____

A1.4.2. S'il s'agit d'un mélange de fonds publics et privés, veuillez expliquer.

____

A1.4.3. Si autre, veuillez préciser

____

A1.4.3. S'il s'agit d'un mélange de fonds publics et privés, veuillez expliquer.

____

A1.4.4. Si autre, veuillez préciser

____

A1.4.4. S'il s'agit d'un mélange de fonds publics et privés, veuillez expliquer.

____

A2. Variation intra-pays

Nous souhaitons comprendre la variation de la prestation des soins dans l'insuffisance rénale (ou maladie rénale au stade terminal [MRST]) au sein d'un même pays, ainsi que la variation entre les pays.

A2.1.1. L'organisation ou la prestation des soins dans l'insuffisance rénale (MRST) diffère-t-elle au niveau régional dans votre pays ?

- ◯ Oui (si possible, veuillez préciser brièvement)
- ◯ Non
- ◯ Inconnu

A2.1.1. Si oui, veuillez préciser

____

A2.1.2. Le coût des soins dans l'insuffisance rénale (MRST) diffère-t-il au niveau régional dans votre pays ?

- ◯ Oui (si possible, veuillez préciser brièvement)
- ◯ Non
- ◯ Inconnu

A2.1.2. Si oui, veuillez préciser

____

A2.1.3. L'organisation ou la prestation des soins dans l'insuffisance rénale (MRST) diffère-t-elle entre les enfants et les adultes dans votre pays ?

- ◯ Oui (si possible, veuillez préciser brièvement)
- ◯ Non
- ◯ Inconnu

A2.1.3. Si oui, veuillez préciser

____

A2.1.4. L'accès au TSR diffère-t-il entre les enfants et les adultes dans votre pays ?

- ◯ Oui (si possible, veuillez préciser brièvement)
- ◯ Non
- ◯ Inconnu

A2.1.4. Si oui, veuillez préciser

____

A2.2.1 Si les services de traitement de suppléance rénale (TSR) ne sont pas égaux entre les adultes et les enfants, quelle est la différence en termes d'accès à l'hémodialyse ?

- ◯ Plus d'accès TSR pour les adultes que pour les enfants
- ◯ Plus d'accès TSR pour les enfants que pour les adultes
- ◯ Accès TSR disponible pour les adultes, indisponible pour les enfants
- ◯ Accès TSR disponible pour les enfants, indisponible pour les adultes

A2.2.2 Si les services de TSR varient entre adultes et enfants, quelle est la différence en termes d'accès à la dialyse péritonéale ?

- ◯ Plus d'accès TSR pour les adultes que pour les enfants
- ◯ Plus d'accès TSR pour les enfants que pour les adultes
- ◯ Accès TSR disponible pour les adultes, non disponible pour les enfants
- ◯ Accès TSR disponible pour les enfants, non disponible pour les adultes

A2.2.3 Si les services de TSR varient entre adultes et enfants, quelle est la différence en termes d'accès à la transplantation rénale ?

- ◯ Plus d'accès TSR pour les adultes que pour les enfants
- ◯ Plus d'accès TSR pour les enfants que pour les adultes
- ◯ Accès TSR disponible pour les adultes, indisponible pour les enfants
- ◯ Accès TSR disponible pour les enfants, indisponible pour les adultes

A3. Supervision

A3.1. Qu'est-ce qui décrit le mieux la gestion/supervision des soins rénaux dans votre pays ? Veuillez cocher toutes les réponses qui s'appliquent.

- ☐ Gérés/supervisés par un organisme national
- ☐ Gérés/supervisés par les autorités provinciales/régionales/départementales uniquement
- ☐ Gérés par des hôpitaux/groupements/organisations individuels
- ☐ Gérés par des ONG
- ☐ Autre (veuillez préciser)
- ☐ Pas de système organisé

A3.1. Si autre, veuillez préciser

____

A3.2. Comment évalueriez-vous l'infrastructure de santé dans votre pays, en termes d'aptitude à fournir des soins dans l'insuffisance rénale (MRST) ?

- ◯ Extrêmement médiocre
- ◯ Médiocre/inférieure à la moyenne
- ◯ Correcte/dans la moyenne
- ◯ Bonne/supérieure à la moyenne
- ◯ Excellente

B. Personnel de santé dans les soins néphrologiques

B1. Responsabilité clinique

B1.1. Qui est le principal responsable clinique de l'administration des soins dans l'insuffisance rénale (MRST) dans votre pays ?

- ◯ Néphrologues
- ◯ Médecins généralistes
- ◯ Infirmiers praticiens
- ◯ Infirmiers spécialisés
- ◯ Équipes pluridisciplinaires
- ◯ Agents de santé/vulgarisateurs
- ◯ Autres spécialistes (veuillez préciser)

B1.1. Si autre, veuillez préciser

____

B2.1.1. Combien de néphrologues y a-t-il environ dans votre pays ? Si le chiffre est inconnu, veuillez ne rien indiquer.

Néphrologues {b2_1_1_neph_fre}
Néphrologues pour adultes {b2_1_1_ad_neph_fre}
Néphrologues pédiatriques {b2_1_1_paed_neph_fre}

B2.1.2. Quel est le pourcentage de néphrologues (pour adultes et pédiatriques confondus) féminins dans votre pays ? Si le chiffre est inconnu, veuillez ne rien indiquer.

____

B2.2.1. Combien de stagiaires en néphrologie y a-t-il environ dans votre pays ? Si le chiffre est inconnu, veuillez ne rien indiquer.

____

B2.2.2. Existe-t-il un programme de formation pour les néphrologues pour adultes dans votre pays ?

- ◯ Oui
- ◯ Non
- ◯ Je n'en suis pas certain(e)

B2.2.3. Si vous avez répondu > à la question B2.2.2 ci-dessus, quelle est la durée du programme de formation ?

- ◯ < 1 an
- ◯ 1 - 2 ans
- ◯ 2 - 4 ans
- ◯ > 4 ans

B2.2.4. Existe-t-il un programme de formation pour les néphrologues pédiatriques dans votre pays ?

- ◯ Oui
- ◯ Non
- ◯ Je n'en suis pas certain(e)

B2.2.5. Si vous avez répondu > à la question B2.2.4 ci-dessus, quelle est la durée du programme de formation ?

- ◯ < 1 an
- ◯ 1 - 2 ans
- ◯ 2 - 4 ans
- ◯ > 4 ans

B2.2.6. Le programme de formation des néphrologues (pour adultes ou pédiatriques) est-il lié à un volet de recherche (par ex., PhD, M.Sc., MPhil, MMed, etc.) ?

- ◯ Oui
- ◯ Non
- ◯ Je n'en suis pas certain(e)

B2.3. Selon vous, existe-t-il une pénurie de prestataires de soins rénaux suivants dans votre pays ? Veuillez cocher toutes les réponses qui s'appliquent.

- ☐ Néphrologues
- ☐ Néphrologues pédiatriques
- ☐ Chirurgiens en transplantation
- ☐ Chirurgiens ou radiologues interventionnels (pouvant créer un accès artério-veineux pour l'hémodialyse)
- ☐ Chirurgiens ou radiologues interventionnels (pouvant créer un accès pour la dialyse péritonéale)
- ☐ Diététiciens
- ☐ Techniciens de laboratoire
- ☐ Radiologues qui réalisent et interprètent des échographies rénales
- ☐ Coordinateurs d'accès vasculaire
- ☐ Conseillers/psychologues
- ☐ Coordinateurs de transplantation
- ☐ Infirmiers en dialyse
- ☐ Infirmiers rénaux
- ☐ Techniciens en dialyse
- ☐ Travailleurs sociaux
- ☐ Médecins en soins palliatifs
- ☐ Infirmiers en soins de soutien rénaux
- ☐ Pas de pénurie du personnel précité

C. Accès aux médicaments et produits de santé essentiels dans les soins rénaux
C1. Capacité de prestation de services de TSR

C1.1. L'hémodialyse en centre (pour adultes et pédiatrique) est-elle disponible dans votre pays ?	◯ Oui<br>◯ Non
C1.1.1. Si oui, combien de centres offrent-ils une hémodialyse (HD) chronique dans votre pays ?	______________________
C1.1.2 L'hémodialyse à domicile (pour adultes et pédiatrique) est-elle disponible dans votre pays ?	◯ Oui<br>◯ Non
C1.2. La dialyse péritonéale (DP) (pour adultes et pédiatrique) est-elle disponible dans votre pays ?	◯ Oui<br>◯ Non
C1.2.1 Si oui, combien de centres offrent-ils une DP chronique dans votre pays ?	______________________
C1.2.2. Si la DP est disponible, dans quelles conditions est-elle accessible dans votre pays ? (Sélectionnez une seule réponse)	◯ DP aiguë uniquement<br>◯ DP aiguë et chronique<br>◯ DP chronique uniquement
C1.2.3. La dialyse péritonéale automatisée (DPA) (pour adultes et pédiatrique) est-elle disponible dans votre pays ?	◯ Oui<br>◯ Non
C1.3. La transplantation rénale chez les adultes est-elle pratiquée dans votre pays ?	◯ Oui<br>◯ Non
C1.4. La transplantation rénale pédiatrique (moins de 18 ans) est-elle pratiquée dans votre pays ?	◯ Oui<br>◯ Non
C1.5.1. Si oui, quelle est l'origine des reins donnés ? (Veuillez sélectionner la réponse la plus appropriée)	◯ Donneurs décédés uniquement<br>◯ Donneurs vivants uniquement<br>◯ Donneurs décédés et vivants
C1.5.2. Si les reins destinés à la transplantation proviennent à la fois de donneurs décédés et de donneurs vivants, quel est le pourcentage de donneurs vivants ?	______________________
C1.5.3. Si la transplantation rénale est disponible dans votre pays, quel est le type de liste(s) d'attente de transplantation rénale ?	◯ Nationale<br>◯ Régionale uniquement<br>◯ Aucune
C1.5.4. Si la transplantation rénale est disponible dans votre pays, combien de centres la pratiquent-ils ?	______________________

C2. Accès aux médicaments et produits de santé essentiels

C2.1. Médicaments et technologies essentiels dans le TSR - Accessibilité, abordabilité financière, plans de remboursement et qualité
(sélectionnez la réponse la plus appropriée à chaque question)

Réduisez la taille de la police si le texte ci-dessous se chevauche.

	Financement public par le gouvernement et gratuit au lieu d'administration	Financement public par le gouvernement, mais certains frais au lieu d'administration	Combinaison d'un système financé par des fonds publics (que la composante financée par des fonds publics soit gratuite ou pas au lieu d'administration) et d'un système privé (veuillez expliquer) Zone de texte	Uniquement privé et payant	Uniquement privé via les compagnies d'assurance maladie	Différents systèmes - programmes fournis par le gouvernement, des ONG et des communautés	Autre (veuillez préciser)
C2.1.1. Pour tous les patients atteints d'une MRC (non dialysés) : Comment les médicaments sont-ils financés ?	○	○	○	○	○	○	○

C2. Accès aux médicaments et produits de santé essentiels

C2.1. Médicaments et technologies essentiels dans le TSR - Accessibilité, abordabilité financière, plans de remboursement et qualité
(sélectionnez la réponse la plus appropriée à chaque question)

Réduisez la taille de la police si le texte ci-dessous se chevauche.

C2.1.2. Pour tous les patients dialysés : Comment les médicaments sont-ils financés ?	○	○	○	○	○	○	○

C2. Accès aux médicaments et produits de santé essentiels

C2.1. Médicaments et technologies essentiels dans le TSR - Accessibilité, abordabilité financière, plans de remboursement et qualité
(sélectionnez la réponse la plus appropriée à chaque question)

Réduisez la taille de la police si le texte ci-dessous se chevauche.

C2.1.3. Pour tous les patients transplantés : Comment les médicaments sont-ils financés ? ○ ○ ○ ○ ○ ○ ○

C2.1.1. Si autre, veuillez préciser __________________________________

C2.1.1. S'il s'agit d'un mélange de fonds publics et privés, veuillez expliquer. __________________________________

C2.1.2. Si autre, veuillez préciser __________________________________

C2.1.2. S'il s'agit d'un mélange de fonds publics et privés, veuillez expliquer. __________________________________

C2.1.3. Si autre, veuillez préciser __________________________________

C2.1.3. S'il s'agit d'un mélange de fonds publics et privés, veuillez expliquer. __________________________________

C3. Préparation au TSR
Soins optimaux de l'insuffisance rénale (MRST) : dans le contexte de la vision, de la mission et des valeurs de l'ISN, nous pensons que tous les patients approchant de l'insuffisance rénale (MRST) doivent bénéficier en temps opportun d'une préparation au TSR, de sorte que les complications et la progression de leur maladie soient atténuées et que leur choix d'options thérapeutiques cliniquement appropriées soit optimisé. Les réponses aux questions suivantes sont importantes pour améliorer notre compréhension de la prestation de services actuelle.

C3.1. Veuillez indiquer la disponibilité des services suivants (tests et traitements) dans le cadre des soins de l'insuffisance rénale (MRST) dans votre pays.

> signifie dans 50 % ou plus des centres (hôpitaux ou cliniques) et > signifie dans moins de 50 % des centres (hôpitaux ou cliniques)

C3.1.1 Gestion du taux d'hémoglobine

	Généralement disponible	Généralement indisponible	Jamais	Inconnu
C3.1.1. Mesure de l'hémoglobine sanguine	◯	◯	◯	◯

C3. Préparation au TSR

Soins optimaux de l'insuffisance rénale (MRST) : dans le contexte de la vision, de la mission et des valeurs de l'ISN, nous pensons que tous les patients approchant de l'insuffisance rénale (MRST) doivent bénéficier en temps opportun d'une préparation au TSR, de sorte que les complications et la progression de leur maladie soient atténuées et que leur choix d'options thérapeutiques cliniquement appropriées soit optimisé. Les réponses aux questions suivantes sont importantes pour améliorer notre compréhension de la prestation de services actuelle.

C3.1. Veuillez indiquer la disponibilité des services suivants (tests et traitements) dans le cadre des soins de l'insuffisance rénale (MRST) dans votre pays.

> signifie dans 50 % ou plus des centres (hôpitaux ou cliniques) et > signifie dans moins de 50 % des centres (hôpitaux ou cliniques)

C3.1.1 Gestion du taux d'hémoglobine

C3.1.2. Mesure des paramètres du fer (fer, ferritine, saturation de la transferrine)	○	○	○	○

C3. Préparation au TSR
Soins optimaux de l'insuffisance rénale (MRST) : dans le contexte de la vision, de la mission et des valeurs de l'ISN, nous pensons que tous les patients approchant de l'insuffisance rénale (MRST) doivent bénéficier en temps opportun d'une préparation au TSR, de sorte que les complications et la progression de leur maladie soient atténuées et que leur choix d'options thérapeutiques cliniquement appropriées soit optimisé. Les réponses aux questions suivantes sont importantes pour améliorer notre compréhension de la prestation de services actuelle.

C3.1. Veuillez indiquer la disponibilité des services suivants (tests et traitements) dans le cadre des soins de l'insuffisance rénale (MRST) dans votre pays.

> signifie dans 50 % ou plus des centres (hôpitaux ou cliniques) et > signifie dans moins de 50 % des centres (hôpitaux ou cliniques)

C3.1.1 Gestion du taux d'hémoglobine

C3.1.3. Mesure des marqueurs de l'inflammation (par ex. protéine C-réactive sérique)	○	○	○	○

C3. Préparation au TSR

Soins optimaux de l'insuffisance rénale (MRST) : dans le contexte de la vision, de la mission et des valeurs de l'ISN, nous pensons que tous les patients approchant de l'insuffisance rénale (MRST) doivent bénéficier en temps opportun d'une préparation au TSR, de sorte que les complications et la progression de leur maladie soient atténuées et que leur choix d'options thérapeutiques cliniquement appropriées soit optimisé. Les réponses aux questions suivantes sont importantes pour améliorer notre compréhension de la prestation de services actuelle.

C3.1. Veuillez indiquer la disponibilité des services suivants (tests et traitements) dans le cadre des soins de l'insuffisance rénale (MRST) dans votre pays.

> signifie dans 50 % ou plus des centres (hôpitaux ou cliniques) et > signifie dans moins de 50 % des centres (hôpitaux ou cliniques)

C3.1.1 Gestion du taux d'hémoglobine

C3.1.4. Fer par voie orale	○	○	○	○

C3. Préparation au TSR

Soins optimaux de l'insuffisance rénale (MRST) : dans le contexte de la vision, de la mission et des valeurs de l'ISN, nous pensons que tous les patients approchant de l'insuffisance rénale (MRST) doivent bénéficier en temps opportun d'une préparation au TSR, de sorte que les complications et la progression de leur maladie soient atténuées et que leur choix d'options thérapeutiques cliniquement appropriées soit optimisé. Les réponses aux questions suivantes sont importantes pour améliorer notre compréhension de la prestation de services actuelle.

C3.1. Veuillez indiquer la disponibilité des services suivants (tests et traitements) dans le cadre des soins de l'insuffisance rénale (MRST) dans votre pays.

> signifie dans 50 % ou plus des centres (hôpitaux ou cliniques) et > signifie dans moins de 50 % des centres (hôpitaux ou cliniques)

C3.1.1 Gestion du taux d'hémoglobine

C3.1.5. Fer par voie parentérale	○	○	○	○

C3. Préparation au TSR
Soins optimaux de l'insuffisance rénale (MRST) : dans le contexte de la vision, de la mission et des valeurs de l'ISN, nous pensons que tous les patients approchant de l'insuffisance rénale (MRST) doivent bénéficier en temps opportun d'une préparation au TSR, de sorte que les complications et la progression de leur maladie soient atténuées et que leur choix d'options thérapeutiques cliniquement appropriées soit optimisé. Les réponses aux questions suivantes sont importantes pour améliorer notre compréhension de la prestation de services actuelle.

C3.1. Veuillez indiquer la disponibilité des services suivants (tests et traitements) dans le cadre des soins de l'insuffisance rénale (MRST) dans votre pays.

> signifie dans 50 % ou plus des centres (hôpitaux ou cliniques) et > signifie dans moins de 50 % des centres (hôpitaux ou cliniques)

C3.1.1 Gestion du taux d'hémoglobine

C3.1.6. Agent stimulant l'érythropoïèse (par ex. érythropoïétine)	○	○	○	○

C3.1.2. Gestion des troubles minéraux osseux

Veuillez indiquer la disponibilité des services suivants (tests et traitements) dans le cadre des soins de l'insuffisance rénale (MRST) dans votre pays.

> signifie dans 50 % ou plus des centres (hôpitaux ou cliniques) et > signifie dans moins de 50 % des centres (hôpitaux ou cliniques)

	Généralement disponible	Généralement indisponible	Jamais	Inconnu
C3.1.2.1. Mesure du calcium sérique	○	○	○	○

C3.1.2. Gestion des troubles minéraux osseux

Veuillez indiquer la disponibilité des services suivants (tests et traitements) dans le cadre des soins de l'insuffisance rénale (MRST) dans votre pays.

> signifie dans 50 % ou plus des centres (hôpitaux ou cliniques) et > signifie dans moins de 50 % des centres (hôpitaux ou cliniques)

C3.1.2.2. Mesure du phosphore sérique	○	○	○	○

C3.1.2. Gestion des troubles minéraux osseux

Veuillez indiquer la disponibilité des services suivants (tests et traitements) dans le cadre des soins de l'insuffisance rénale (MRST) dans votre pays.

> signifie dans 50 % ou plus des centres (hôpitaux ou cliniques) et > signifie dans moins de 50 % des centres (hôpitaux ou cliniques)

C3.1.2.3. Mesure de l'hormone parathyroïdienne (HPT) sérique	○	○	○	○

C3.1.2. Gestion des troubles minéraux osseux

Veuillez indiquer la disponibilité des services suivants (tests et traitements) dans le cadre des soins de l'insuffisance rénale (MRST) dans votre pays.

> signifie dans 50 % ou plus des centres (hôpitaux ou cliniques) et > signifie dans moins de 50 % des centres (hôpitaux ou cliniques)

C3.1.2.4. Chélateurs calciques du phosphate	○	○	○	○

C3.1.2. Gestion des troubles minéraux osseux

Veuillez indiquer la disponibilité des services suivants (tests et traitements) dans le cadre des soins de l'insuffisance rénale (MRST) dans votre pays.

> signifie dans 50 % ou plus des centres (hôpitaux ou cliniques) et > signifie dans moins de 50 % des centres (hôpitaux ou cliniques)

C3.1.2.5. Chélateurs non calciques du phosphate (par ex. sévélamer)	○	○	○	○

C3.1.2. Gestion des troubles minéraux osseux

Veuillez indiquer la disponibilité des services suivants (tests et traitements) dans le cadre des soins de l'insuffisance rénale (MRST) dans votre pays.

> signifie dans 50 % ou plus des centres (hôpitaux ou cliniques) et > signifie dans moins de 50 % des centres (hôpitaux ou cliniques)

C3.1.2.6. Cinacalcet	○	○	○	○

C3.1.2. Gestion des troubles minéraux osseux

Veuillez indiquer la disponibilité des services suivants (tests et traitements) dans le cadre des soins de l'insuffisance rénale (MRST) dans votre pays.

> signifie dans 50 % ou plus des centres (hôpitaux ou cliniques) et > signifie dans moins de 50 % des centres (hôpitaux ou cliniques)

C3.1.2.7. Services chirurgicaux de parathyroïdectomie	○	○	○	○

C3.1.3. Gestion des troubles électrolytiques et de l'acidose métabolique chronique

Veuillez indiquer la disponibilité des services suivants (tests et traitements) dans le cadre des soins de l'insuffisance rénale (MRST) dans votre pays.
> signifie dans 50 % ou plus des centres (hôpitaux ou cliniques) et > signifie dans moins de 50 % des centres (hôpitaux ou cliniques)

	Généralement disponible	Généralement indisponible	Jamais	Inconnu
C3.1.3.1. Mesure des électrolytes sériques (sodium, potassium, chlorure, etc.)	○	○	○	○

C3.1.3. Gestion des troubles électrolytiques et de l'acidose métabolique chronique

Veuillez indiquer la disponibilité des services suivants (tests et traitements) dans le cadre des soins de l'insuffisance rénale (MRST) dans votre pays.
> signifie dans 50 % ou plus des centres (hôpitaux ou cliniques) et > signifie dans moins de 50 % des centres (hôpitaux ou cliniques)

C3.1.3.2. Mesure du bicarbonate sérique	○	○	○	○

C3.1.3. Gestion des troubles électrolytiques et de l'acidose métabolique chronique

Veuillez indiquer la disponibilité des services suivants (tests et traitements) dans le cadre des soins de l'insuffisance rénale (MRST) dans votre pays.
> signifie dans 50 % ou plus des centres (hôpitaux ou cliniques) et > signifie dans moins de 50 % des centres (hôpitaux ou cliniques)

C3.1.3.3. Résines échangeuses de potassium (par ex. Kayexalate, patiromer, zirconium sodique)	○	○	○	○

C3.1.3. Gestion des troubles électrolytiques et de l'acidose métabolique chronique

Veuillez indiquer la disponibilité des services suivants (tests et traitements) dans le cadre des soins de l'insuffisance rénale (MRST) dans votre pays.
> signifie dans 50 % ou plus des centres (hôpitaux ou cliniques) et > signifie dans moins de 50 % des centres (hôpitaux ou cliniques)

C3.1.3.4. Bicarbonate de sodium par voie orale	○	○	○	○

C3.1.4. Gestion de la pression artérielle

Veuillez indiquer la disponibilité des services suivants (tests et traitements) dans le cadre des soins de l'insuffisance rénale (MRST) dans votre pays.
> signifie dans 50 % ou plus des centres (hôpitaux ou cliniques) et > signifie dans moins de 50 % des centres (hôpitaux ou cliniques)

	Généralement disponible	Généralement indisponible	Jamais	Inconnu
C3.1.4.1. Surveillance analogique de la PA	○	○	○	○

C3.1.4. Gestion de la pression artérielle

Veuillez indiquer la disponibilité des services suivants (tests et traitements) dans le cadre des soins de l'insuffisance rénale (MRST) dans votre pays.
> signifie dans 50 % ou plus des centres (hôpitaux ou cliniques) et > signifie dans moins de 50 % des centres (hôpitaux ou cliniques)

C3.1.4.2. Surveillance automatisée de la PA (à domicile ou au cabinet)	○	○	○	○

C3.1.4. Gestion de la pression artérielle

Veuillez indiquer la disponibilité des services suivants (tests et traitements) dans le cadre des soins de l'insuffisance rénale (MRST) dans votre pays.
> signifie dans 50 % ou plus des centres (hôpitaux ou cliniques) et > signifie dans moins de 50 % des centres (hôpitaux ou cliniques)

C3.1.4.3. Surveillance ambulatoire de la PA (SAPA)	○	○	○	○

C3.1.5. Gestion des symptômes courants associés à l'insuffisance rénale (MRST) (prurit urémique, jambes sans repos, douleur)

Veuillez indiquer la disponibilité des services suivants (tests et traitements) dans le cadre des soins de l'insuffisance rénale (MRST) dans votre pays.
> signifie dans 50 % ou plus des centres (hôpitaux ou cliniques) et > signifie dans moins de 50 % des centres (hôpitaux ou cliniques)

	Généralement disponible	Généralement indisponible	Jamais	Inconnu
C3.1.5.1. Gabapentinoïdes (gabapentine ou prégabaline)	○	○	○	○

C3.1.5. Gestion des symptômes courants associés à l'insuffisance rénale (MRST) (prurit urémique, jambes sans repos, douleur)

Veuillez indiquer la disponibilité des services suivants (tests et traitements) dans le cadre des soins de l'insuffisance rénale (MRST) dans votre pays.
> signifie dans 50 % ou plus des centres (hôpitaux ou cliniques) et > signifie dans moins de 50 % des centres (hôpitaux ou cliniques)

C3.1.5.2. Opioïdes non morphiniques (par ex. hydromorphone, oxycodone, méthadone et/ou fentanyl sublingual ou transdermique)	○	○	○	○

C4.1 Services nutritionnels

C4.1.1. Veuillez indiquer la disponibilité des services nutritionnels suivants dans le cadre des soins rénaux dans votre pays.
> signifie dans 50 % ou plus des centres (hôpitaux ou cliniques) et > signifie dans moins de 50 % des centres (hôpitaux ou cliniques)

	Généralement disponible	Généralement indisponible	Jamais	Inconnu
C4.1.1.1. Conseils diététiques par une personne qualifiée en nutrition (par ex. un diététicien)	○	○	○	○

C4.1 Services nutritionnels

C4.1.1. Veuillez indiquer la disponibilité des services nutritionnels suivants dans le cadre des soins rénaux dans votre pays.
> signifie dans 50 % ou plus des centres (hôpitaux ou cliniques) et > signifie dans moins de 50 % des centres (hôpitaux ou cliniques)

C4.1.1.2. Mesure de l'albumine sérique	○	○	○	○

C4.1 Services nutritionnels

C4.1.1. Veuillez indiquer la disponibilité des services nutritionnels suivants dans le cadre des soins rénaux dans votre pays.
> signifie dans 50 % ou plus des centres (hôpitaux ou cliniques) et > signifie dans moins de 50 % des centres (hôpitaux ou cliniques)

C4.1.1.3. Compléments alimentaires par voie orale (par ex. vitamines, compléments nutritionnels oraux)	○	○	○	○

C5. Traitement par dialyse - Qualité et choix

C5.1. Choix de la modalité

Veuillez indiquer la disponibilité des services suivants dans le cadre des soins par dialyse dans votre pays.
> signifie dans 50 % ou plus des centres (hôpitaux ou cliniques) et > signifie dans moins de 50 % des centres (hôpitaux ou cliniques).

	Généralement disponible	Généralement indisponible	Jamais	Inconnu	S.O. (dialyse indisponible)
C5.1.1. Hémodialyse en centre	○	○	○	○	○

C5. Traitement par dialyse - Qualité et choix

C5.1. Choix de la modalité

Veuillez indiquer la disponibilité des services suivants dans le cadre des soins par dialyse dans votre pays.
> signifie dans 50 % ou plus des centres (hôpitaux ou cliniques) et > signifie dans moins de 50 % des centres (hôpitaux ou cliniques).

C5.1.2. Hémodialyse à domicile	○	○	○	○	○

C5. Traitement par dialyse - Qualité et choix

C5.1. Choix de la modalité

Veuillez indiquer la disponibilité des services suivants dans le cadre des soins par dialyse dans votre pays.
> signifie dans 50 % ou plus des centres (hôpitaux ou cliniques) et > signifie dans moins de 50 % des centres (hôpitaux ou cliniques).

C5.1.3. Dialyse péritonéale	○	○	○	○	○

C5.1. Qualité

Veuillez indiquer la disponibilité des services suivants dans le cadre des soins par dialyse dans votre pays.
> signifie dans 50 % ou plus des centres (hôpitaux ou cliniques) et > signifie dans moins de 50 % des centres (hôpitaux ou cliniques).

	Généralement disponible	Généralement indisponible	Jamais	Inconnu	S.O. (dialyse indisponible)
C5.1.4. Service d'hémodialyse en centre à une fréquence adéquate (traitement trois fois par semaine pendant trois ou quatre heures)	○	○	○	○	○

C5.1. Qualité

Veuillez indiquer la disponibilité des services suivants dans le cadre des soins par dialyse dans votre pays.
> signifie dans 50 % ou plus des centres (hôpitaux ou cliniques) et > signifie dans moins de 50 % des centres (hôpitaux ou cliniques).

C5.1.5. Hémodialyse à domicile (traitement trois fois par semaine pendant trois ou quatre heures)	○	○	○	○	○

C5.1. Qualité

Veuillez indiquer la disponibilité des services suivants dans le cadre des soins par dialyse dans votre pays.
> signifie dans 50 % ou plus des centres (hôpitaux ou cliniques) et > signifie dans moins de 50 % des centres (hôpitaux ou cliniques).

C5.1.6. Échanges en dialyse péritonéale à une fréquence adéquate (3 à 4 par jour ou cycles équivalents en DP automatisée)	○	○	○	○	○

C5.1. Qualité

Veuillez indiquer la disponibilité des services suivants dans le cadre des soins par dialyse dans votre pays.
> signifie dans 50 % ou plus des centres (hôpitaux ou cliniques) et > signifie dans moins de 50 % des centres (hôpitaux ou cliniques).

C5.1.7. Détermination de l'efficacité de la dialyse péritonéale (à savoir par mesure du pourcentage de réduction d'urée [PRU] et/ou Kt/V)	○	○	○	○	○

C5.1. Qualité

Veuillez indiquer la disponibilité des services suivants dans le cadre des soins par dialyse dans votre pays.
> signifie dans 50 % ou plus des centres (hôpitaux ou cliniques) et > signifie dans moins de 50 % des centres (hôpitaux ou cliniques).

C5.1.8. Services financièrement abordables de transport des patients	○	○	○	○	○

C6. Transplantation - Qualité et choix
Choix de la transplantation : dans le contexte de la vision, de la mission et des valeurs de l'ISN, nous pensons que tous les patients transplantés rénaux doivent recevoir un service de qualité qui les aide à gérer leur organe transplanté et leur permet d'avoir la meilleure qualité de vie possible. Les réponses aux questions suivantes sont importantes pour améliorer notre compréhension de la prestation de services actuelle.

C6.1. Veuillez indiquer la disponibilité des services suivants dans le cadre de la transplantation dans votre pays.
> signifie dans 50 % ou plus des centres (hôpitaux ou cliniques) et > signifie dans moins de 50 % des centres (hôpitaux ou cliniques).

Si la transplantation n'est PAS disponible dans votre pays, sélectionnez S.O.

	Généralement disponible	Généralement indisponible	Jamais	Inconnu	S.O. (transplantation indisponible)
C6.1.1. Mise à disposition précoce d'informations culturellement adaptées, destinées aux patients, aux proches et aux soignants, sur les risques et les avantages de la transplantation, ainsi que d'explications claires relatives aux tests, procédures et résultats	○	○	○	○	○

C6. Transplantation - Qualité et choix
Choix de la transplantation : dans le contexte de la vision, de la mission et des valeurs de l'ISN, nous pensons que tous les patients transplantés rénaux doivent recevoir un service de qualité qui les aide à gérer leur organe transplanté et leur permet d'avoir la meilleure qualité de vie possible. Les réponses aux questions suivantes sont importantes pour améliorer notre compréhension de la prestation de services actuelle.

C6.1. Veuillez indiquer la disponibilité des services suivants dans le cadre de la transplantation dans votre pays.
> signifie dans 50 % ou plus des centres (hôpitaux ou cliniques) et > signifie dans moins de 50 % des centres (hôpitaux ou cliniques).

Si la transplantation n'est PAS disponible dans votre pays, sélectionnez S.O.

C6.1.2. Traitement préventif efficace en vue du contrôle des infections (par ex. antiviraux, antifongiques, etc.)	○	○	○	○	○

C6. Transplantation - Qualité et choix
Choix de la transplantation : dans le contexte de la vision, de la mission et des valeurs de l'ISN, nous pensons que tous les patients transplantés rénaux doivent recevoir un service de qualité qui les aide à gérer leur organe transplanté et leur permet d'avoir la meilleure qualité de vie possible. Les réponses aux questions suivantes sont importantes pour améliorer notre compréhension de la prestation de services actuelle.

C6.1. Veuillez indiquer la disponibilité des services suivants dans le cadre de la transplantation dans votre pays.
> signifie dans 50 % ou plus des centres (hôpitaux ou cliniques) et > signifie dans moins de 50 % des centres (hôpitaux ou cliniques).

Si la transplantation n'est PAS disponible dans votre pays, sélectionnez S.O.

C6.1.3. Accès en temps opportun à l'espace d'opération de transplantation rénale	○	○	○	○	○

C6. Transplantation - Qualité et choix

Choix de la transplantation : dans le contexte de la vision, de la mission et des valeurs de l'ISN, nous pensons que tous les patients transplantés rénaux doivent recevoir un service de qualité qui les aide à gérer leur organe transplanté et leur permet d'avoir la meilleure qualité de vie possible. Les réponses aux questions suivantes sont importantes pour améliorer notre compréhension de la prestation de services actuelle.

C6.1. Veuillez indiquer la disponibilité des services suivants dans le cadre de la transplantation dans votre pays.
> signifie dans 50 % ou plus des centres (hôpitaux ou cliniques) et > signifie dans moins de 50 % des centres (hôpitaux ou cliniques).

Si la transplantation n'est PAS disponible dans votre pays, sélectionnez S.O.

C6.1.4. Traitement immunosuppresseur et antirejet adapté	○	○	○	○	○

C6. Transplantation - Qualité et choix
Choix de la transplantation : dans le contexte de la vision, de la mission et des valeurs de l'ISN, nous pensons que tous les patients transplantés rénaux doivent recevoir un service de qualité qui les aide à gérer leur organe transplanté et leur permet d'avoir la meilleure qualité de vie possible. Les réponses aux questions suivantes sont importantes pour améliorer notre compréhension de la prestation de services actuelle.

C6.1. Veuillez indiquer la disponibilité des services suivants dans le cadre de la transplantation dans votre pays.
> signifie dans 50 % ou plus des centres (hôpitaux ou cliniques) et > signifie dans moins de 50 % des centres (hôpitaux ou cliniques).

Si la transplantation n'est PAS disponible dans votre pays, sélectionnez S.O.

C6.1.5. Équipements appropriés de surveillance de l'administration des médicaments immunosuppresseurs	○	○	○	○	○

C6. Transplantation - Qualité et choix
Choix de la transplantation : dans le contexte de la vision, de la mission et des valeurs de l'ISN, nous pensons que tous les patients transplantés rénaux doivent recevoir un service de qualité qui les aide à gérer leur organe transplanté et leur permet d'avoir la meilleure qualité de vie possible. Les réponses aux questions suivantes sont importantes pour améliorer notre compréhension de la prestation de services actuelle.

C6.1. Veuillez indiquer la disponibilité des services suivants dans le cadre de la transplantation dans votre pays.
> signifie dans 50 % ou plus des centres (hôpitaux ou cliniques) et > signifie dans moins de 50 % des centres (hôpitaux ou cliniques).

Si la transplantation n'est PAS disponible dans votre pays, sélectionnez S.O.

C6.1.6. Équipe pluridisciplinaire pour soutenir les patients transplantés rénaux	○	○	○	○	○

C6. Transplantation - Qualité et choix
Choix de la transplantation : dans le contexte de la vision, de la mission et des valeurs de l'ISN, nous pensons que tous les patients transplantés rénaux doivent recevoir un service de qualité qui les aide à gérer leur organe transplanté et leur permet d'avoir la meilleure qualité de vie possible. Les réponses aux questions suivantes sont importantes pour améliorer notre compréhension de la prestation de services actuelle.

C6.1. Veuillez indiquer la disponibilité des services suivants dans le cadre de la transplantation dans votre pays.
> signifie dans 50 % ou plus des centres (hôpitaux ou cliniques) et > signifie dans moins de 50 % des centres (hôpitaux ou cliniques).

Si la transplantation n'est PAS disponible dans votre pays, sélectionnez S.O.

C6.1.7. Cadre standard pour l'obtention d'organes (par ex. législation relative à la mort cérébrale)	○	○	○	○	○

C7. Gestion rénale conservatrice (GRC)
Gestion rénale conservatrice : la gestion rénale conservatrice est définie comme le choix du patient pour des soins globaux, centrés sur le patient, sans recours à un TSR (Traitement de Suppléance Rénale) pour les patients atteints d'une MRC de stade G5. Les objectifs de la gestion rénale conservatrice sont de soutenir les patients atteints d'une MRC de stade G5 qui ne reçoivent pas de TSR, en optimisant la qualité de vie, en gérant les symptômes, en traitant la détresse psychosociale, en facilitant la planification préalable des soins et, le cas échéant, en préservant la fonction rénale résiduelle. Ces soins incluent le soutien de la famille et des soignants du patient et se poursuivent tout au long de la maladie. La GRC est appropriée pour les patients qui sont peu susceptibles de retirer un bénéfice d'un TSR ou qui choisissent de ne pas débuter un TSR. Nous reconnaissons que les patients peuvent recevoir des soins conservateurs similaires lorsque des contraintes liées aux ressources (système de soins de santé ou patient) empêchent ou limitent l'accès au TSR. Nous utilisons alors le terme de soins conservateurs par choix restreint.
Nous souhaitons en savoir plus sur la capacité à fournir une gestion rénale conservatrice ou des soins conservateurs par choix restreint dans votre pays (c'est-à-dire la capacité à soutenir/gérer les patients qui ne reçoivent pas de TSR malgré une MRC de stade G5).

C7.1. Compte tenu des définitions ci-dessus, des soins conservateurs sont-ils disponibles dans votre pays?

	Généralement disponible	Généralement indisponible	S.O. (GRC indisponible)	Inconnu
C7.1.1. Gestion rénale conservatrice établie choisie à la suite d'une prise de décision partagée (lorsque le TSR est facilement disponible)	○	○	○	○

C7. Gestion rénale conservatrice (GRC)
Gestion rénale conservatrice : la gestion rénale conservatrice est définie comme le choix du patient pour des soins globaux, centrés sur le patient, sans recours à un TSR (Traitement de Suppléance Rénale) pour les patients atteints d'une MRC de stade G5. Les objectifs de la gestion rénale conservatrice sont de soutenir les patients atteints d'une MRC de stade G5 qui ne reçoivent pas de TSR, en optimisant la qualité de vie, en gérant les symptômes, en traitant la détresse psychosociale, en facilitant la planification préalable des soins et, le cas échéant, en préservant la fonction rénale résiduelle. Ces soins incluent le soutien de la famille et des soignants du patient et se poursuivent tout au long de la maladie. La GRC est appropriée pour les patients qui sont peu susceptibles de retirer un bénéfice d'un TSR ou qui choisissent de ne pas débuter un TSR. Nous reconnaissons que les patients peuvent recevoir des soins conservateurs similaires lorsque des contraintes liées aux ressources (système de soins de santé ou patient) empêchent ou limitent l'accès au TSR. Nous utilisons alors le terme de soins conservateurs par choix restreint.
Nous souhaitons en savoir plus sur la capacité à fournir une gestion rénale conservatrice ou des soins conservateurs par choix restreint dans votre pays (c'est-à-dire la capacité à soutenir/gérer les patients qui ne reçoivent pas de TSR malgré une MRC de stade G5).

C7.1. Compte tenu des définitions ci-dessus, des soins conservateurs sont-ils disponibles dans votre pays?

C7.1.2. Soins conservateurs par choix restreint (lorsque des contraintes liées aux ressources empêchent ou limitent l'accès au TSR)	○	○	○	○

C7. Gestion rénale conservatrice (GRC)

Gestion rénale conservatrice : la gestion rénale conservatrice est définie comme le choix du patient pour des soins globaux, centrés sur le patient, sans recours à un TSR (Traitement de Suppléance Rénale) pour les patients atteints d'une MRC de stade G5. Les objectifs de la gestion rénale conservatrice sont de soutenir les patients atteints d'une MRC de stade G5 qui ne reçoivent pas de TSR, en optimisant la qualité de vie, en gérant les symptômes, en traitant la détresse psychosociale, en facilitant la planification préalable des soins et, le cas échéant, en préservant la fonction rénale résiduelle. Ces soins incluent le soutien de la famille et des soignants du patient et se poursuivent tout au long de la maladie. La GRC est appropriée pour les patients qui sont peu susceptibles de retirer un bénéfice d'un TSR ou qui choisissent de ne pas débuter un TSR. Nous reconnaissons que les patients peuvent recevoir des soins conservateurs similaires lorsque des contraintes liées aux ressources (système de soins de santé ou patient) empêchent ou limitent l'accès au TSR. Nous utilisons alors le terme de soins conservateurs par choix restreint.

Nous souhaitons en savoir plus sur la capacité à fournir une gestion rénale conservatrice ou des soins conservateurs par choix restreint dans votre pays (c'est-à-dire la capacité à soutenir/gérer les patients qui ne reçoivent pas de TSR malgré une MRC de stade G5).

C7.1. Compte tenu des définitions ci-dessus, des soins conservateurs sont-ils disponibles dans votre pays?

C7.1.3. Soins conservateurs par choix restreint (lorsqu'il n'y a pas de contraintes liées aux ressources qui empêchent ou limitent l'accès au TSR)	○	○	○	○

C7.2. Veuillez indiquer la probabilité moyenne qu'un néphrologue de votre pays propose une gestion rénale conservatrice comme option thérapeutique aux patients atteints d'une MRC de stade G5 ?

○ Toujours
○ Souvent
○ Parfois
○ Rarement
○ Jamais
○ Inconnu

C7.3. Lorsque l'accès à la dialyse repose sur un choix restreint, quelle est la principale raison ?

○ Financière - système de soins de santé
○ Financière - patient
○ Géographique

C7.4. Veuillez indiquer la disponibilité de la structure et du processus de prestation de la gestion rénale conservatrice (à savoir des soins conservateurs choisis ou médicalement conseillés lorsque le TSR est facilement disponible) pour les patients atteints d'une MRC de stade G5) :

> signifie dans 50 % ou plus des centres (hôpitaux ou cliniques) et > signifie dans moins de 50 % des centres (hôpitaux ou cliniques).

	Généralement disponible	Généralement indisponible	indisponible	Inconnu
C7.4.1. Infrastructure établie pour aider les patients à suivre un trajet de gestion rénale conservatrice	○	○	○	○

C7.4. Veuillez indiquer la disponibilité de la structure et du processus de prestation de la gestion rénale conservatrice (à savoir des soins conservateurs choisis ou médicalement conseillés lorsque le TSR est facilement disponible) pour les patients atteints d'une MRC de stade G5) :

> signifie dans 50 % ou plus des centres (hôpitaux ou cliniques) et > signifie dans moins de 50 % des centres (hôpitaux ou cliniques).

C7.4.2. Outils de prise de décision partagée pour les patients et les prestataires afin d'aider à prendre la décision de gestion conservatrice du rein	○	○	○	○

C7.4. Veuillez indiquer la disponibilité de la structure et du processus de prestation de la gestion rénale conservatrice (à savoir des soins conservateurs choisis ou médicalement conseillés lorsque le TSR est facilement disponible) pour les patients atteints d'une MRC de stade G5) :

> signifie dans 50 % ou plus des centres (hôpitaux ou cliniques) et > signifie dans moins de 50 % des centres (hôpitaux ou cliniques).

C7.4.3. Services établis où les patients bénéficiant d'une gestion rénale conservatrice peuvent être vus à domicile, dans une maison de soins ou dans une maison de repos s'ils ne sont pas en mesure de se rendre à l'hôpital ou à la clinique	○	○	○	○

C7.4. Veuillez indiquer la disponibilité de la structure et du processus de prestation de la gestion rénale conservatrice (à savoir des soins conservateurs choisis ou médicalement conseillés lorsque le TSR est facilement disponible) pour les patients atteints d'une MRC de stade G5) :

> signifie dans 50 % ou plus des centres (hôpitaux ou cliniques) et > signifie dans moins de 50 % des centres (hôpitaux ou cliniques).

C7.4.4. Instructions écrites, plan ou directives de gestion conservatrice du rein couvrant la préservation de la fonction rénale résiduelle, le contrôle des symptômes, la planification préalable des soins et les soins de fin de vie	○	○	○	○

C7.4. Veuillez indiquer la disponibilité de la structure et du processus de prestation de la gestion rénale conservatrice (à savoir des soins conservateurs choisis ou médicalement conseillés lorsque le TSR est facilement disponible) pour les patients atteints d'une MRC de stade G5) :

> signifie dans 50 % ou plus des centres (hôpitaux ou cliniques) et > signifie dans moins de 50 % des centres (hôpitaux ou cliniques).

C7.4.5. Équipe pluridisciplinaire	○	○	○	○

C7.4. Veuillez indiquer la disponibilité de la structure et du processus de prestation de la gestion rénale conservatrice (à savoir des soins conservateurs choisis ou médicalement conseillés lorsque le TSR est facilement disponible) pour les patients atteints d'une MRC de stade G5) :

> signifie dans 50 % ou plus des centres (hôpitaux ou cliniques) et > signifie dans moins de 50 % des centres (hôpitaux ou cliniques).

C7.4.6. L'équipe pluridisciplinaire inclut des liens formels avec des cliniciens rénaux formés aux soins conservateurs	○	○	○	○

C7.4. Veuillez indiquer la disponibilité de la structure et du processus de prestation de la gestion rénale conservatrice (à savoir des soins conservateurs choisis ou médicalement conseillés lorsque le TSR est facilement disponible) pour les patients atteints d'une MRC de stade G5) :

> signifie dans 50 % ou plus des centres (hôpitaux ou cliniques) et > signifie dans moins de 50 % des centres (hôpitaux ou cliniques).

C7.4.7. L'équipe pluridisciplinaire inclut des liens formels avec les soins palliatifs	○	○	○	○

C7.4. Veuillez indiquer la disponibilité de la structure et du processus de prestation de la gestion rénale conservatrice (à savoir des soins conservateurs choisis ou médicalement conseillés lorsque le TSR est facilement disponible) pour les patients atteints d'une MRC de stade G5) :

> signifie dans 50 % ou plus des centres (hôpitaux ou cliniques) et > signifie dans moins de 50 % des centres (hôpitaux ou cliniques).

C7.4.8. Utilisation régulière d'outils de dépistage validés, de documentation et de la gestion des symptômes	○	○	○	○

C7.4. Veuillez indiquer la disponibilité de la structure et du processus de prestation de la gestion rénale conservatrice (à savoir des soins conservateurs choisis ou médicalement conseillés lorsque le TSR est facilement disponible) pour les patients atteints d'une MRC de stade G5) :

> signifie dans 50 % ou plus des centres (hôpitaux ou cliniques) et > signifie dans moins de 50 % des centres (hôpitaux ou cliniques).

C7.4.9. Disponibilité de médicaments essentiels contre la douleur et pour les soins palliatifs à tous les niveaux de soins (primaires et spécialisés)	○	○	○	○

C7.4. Veuillez indiquer la disponibilité de la structure et du processus de prestation de la gestion rénale conservatrice (à savoir des soins conservateurs choisis ou médicalement conseillés lorsque le TSR est facilement disponible) pour les patients atteints d'une MRC de stade G5) :

> signifie dans 50 % ou plus des centres (hôpitaux ou cliniques) et > signifie dans moins de 50 % des centres (hôpitaux ou cliniques).

C7.4.10. Infrastructure pour documenter et partager les entretiens sur la planification préalable des soins, y compris les décisions concernant le lieu de prédilection des soins, le décès et la réanimation	○	○	○	○

C7.4. Veuillez indiquer la disponibilité de la structure et du processus de prestation de la gestion rénale conservatrice (à savoir des soins conservateurs choisis ou médicalement conseillés lorsque le TSR est facilement disponible) pour les patients atteints d'une MRC de stade G5) :

> signifie dans 50 % ou plus des centres (hôpitaux ou cliniques) et > signifie dans moins de 50 % des centres (hôpitaux ou cliniques).

C7.4.11. Fourniture d'un soutien psychologique, social et spirituel	○	○	○	○

C7.4. Veuillez indiquer la disponibilité de la structure et du processus de prestation de la gestion rénale conservatrice (à savoir des soins conservateurs choisis ou médicalement conseillés lorsque le TSR est facilement disponible) pour les patients atteints d'une MRC de stade G5) :

> signifie dans 50 % ou plus des centres (hôpitaux ou cliniques) et > signifie dans moins de 50 % des centres (hôpitaux ou cliniques).

C7.4.12. Formation des prestataires de soins à la gestion des symptômes	○	○	○	○

C7.4. Veuillez indiquer la disponibilité de la structure et du processus de prestation de la gestion rénale conservatrice (à savoir des soins conservateurs choisis ou médicalement conseillés lorsque le TSR est facilement disponible) pour les patients atteints d'une MRC de stade G5) :

> signifie dans 50 % ou plus des centres (hôpitaux ou cliniques) et > signifie dans moins de 50 % des centres (hôpitaux ou cliniques).

C7.4.13. Formation des prestataires de soins à la planification préalable des soins	○	○	○	○

C7.4. Veuillez indiquer la disponibilité de la structure et du processus de prestation de la gestion rénale conservatrice (à savoir des soins conservateurs choisis ou médicalement conseillés lorsque le TSR est facilement disponible) pour les patients atteints d'une MRC de stade G5) :

> signifie dans 50 % ou plus des centres (hôpitaux ou cliniques) et > signifie dans moins de 50 % des centres (hôpitaux ou cliniques).

C7.4.14. Collecte systématique de données sur le nombre de patients bénéficiant d'une gestion rénale conservatrice et leurs résultats	○	○	○	○

C7.5. Veuillez indiquer la disponibilité de la structure et du processus de prestation de soins conservateurs par choix restreint (à savoir des soins conservateurs destinés aux patients pour lesquels des contraintes liées aux ressources empêchent ou limitent l'accès au TSR) pour les patients atteints d'une MRC de stade G5) :

> signifie dans 50 % ou plus des centres (hôpitaux ou cliniques) et > signifie dans moins de 50 % des centres (hôpitaux ou cliniques).

	Généralement disponible	Généralement indisponible	indisponible	Inconnu
C7.5.1. Infrastructure établie pour soutenir les patients recevant des soins conservateurs par choix restreint	○	○	○	○

C7.5. Veuillez indiquer la disponibilité de la structure et du processus de prestation de soins conservateurs par choix restreint (à savoir des soins conservateurs destinés aux patients pour lesquels des contraintes liées aux ressources empêchent ou limitent l'accès au TSR) pour les patients atteints d'une MRC de stade G5) :

> signifie dans 50 % ou plus des centres (hôpitaux ou cliniques) et > signifie dans moins de 50 % des centres (hôpitaux ou cliniques).

C7.5.2. Services établis où les patients recevant des soins conservateurs par choix restreint peuvent être vus à domicile, dans une maison de soins ou dans une maison de repos s'ils ne sont pas en mesure de se rendre à l'hôpital ou à la clinique	○	○	○	○

C7.5. Veuillez indiquer la disponibilité de la structure et du processus de prestation de soins conservateurs par choix restreint (à savoir des soins conservateurs destinés aux patients pour lesquels des contraintes liées aux ressources empêchent ou limitent l'accès au TSR) pour les patients atteints d'une MRC de stade G5) :

> signifie dans 50 % ou plus des centres (hôpitaux ou cliniques) et > signifie dans moins de 50 % des centres (hôpitaux ou cliniques).

C7.5.3. Instructions écrites, plan ou directives de soins conservateurs couvrant la préservation de la fonction rénale résiduelle, le contrôle des symptômes, la planification préalable des soins et les soins de fin de vie	◯	◯	◯	◯

C7.5. Veuillez indiquer la disponibilité de la structure et du processus de prestation de soins conservateurs par choix restreint (à savoir des soins conservateurs destinés aux patients pour lesquels des contraintes liées aux ressources empêchent ou limitent l'accès au TSR) pour les patients atteints d'une MRC de stade G5) :

> signifie dans 50 % ou plus des centres (hôpitaux ou cliniques) et > signifie dans moins de 50 % des centres (hôpitaux ou cliniques).

C7.5.4. Équipe pluridisciplinaire	○	○	○	○

C7.5. Veuillez indiquer la disponibilité de la structure et du processus de prestation de soins conservateurs par choix restreint (à savoir des soins conservateurs destinés aux patients pour lesquels des contraintes liées aux ressources empêchent ou limitent l'accès au TSR) pour les patients atteints d'une MRC de stade G5) :

> signifie dans 50 % ou plus des centres (hôpitaux ou cliniques) et > signifie dans moins de 50 % des centres (hôpitaux ou cliniques).

C7.5.5. L'équipe pluridisciplinaire inclut des liens formels avec des cliniciens rénaux formés aux soins conservateurs	○	○	○	○

C7.5. Veuillez indiquer la disponibilité de la structure et du processus de prestation de soins conservateurs par choix restreint (à savoir des soins conservateurs destinés aux patients pour lesquels des contraintes liées aux ressources empêchent ou limitent l'accès au TSR) pour les patients atteints d'une MRC de stade G5) :

> signifie dans 50 % ou plus des centres (hôpitaux ou cliniques) et > signifie dans moins de 50 % des centres (hôpitaux ou cliniques).

C7.5.6. L'équipe pluridisciplinaire inclut des liens formels avec les soins palliatifs	○	○	○	○

C7.5. Veuillez indiquer la disponibilité de la structure et du processus de prestation de soins conservateurs par choix restreint (à savoir des soins conservateurs destinés aux patients pour lesquels des contraintes liées aux ressources empêchent ou limitent l'accès au TSR) pour les patients atteints d'une MRC de stade G5) :

> signifie dans 50 % ou plus des centres (hôpitaux ou cliniques) et > signifie dans moins de 50 % des centres (hôpitaux ou cliniques).

C7.5.7. Utilisation régulière d'outils de dépistage validés, de documentation et de la gestion des symptômes	○	○	○	○

C7.5. Veuillez indiquer la disponibilité de la structure et du processus de prestation de soins conservateurs par choix restreint (à savoir des soins conservateurs destinés aux patients pour lesquels des contraintes liées aux ressources empêchent ou limitent l'accès au TSR) pour les patients atteints d'une MRC de stade G5) :

> signifie dans 50 % ou plus des centres (hôpitaux ou cliniques) et > signifie dans moins de 50 % des centres (hôpitaux ou cliniques).

C7.5.8. Disponibilité de médicaments essentiels contre la douleur et pour les soins palliatifs à tous les niveaux de soins (primaires et spécialisés)	○	○	○	○

C7.5. Veuillez indiquer la disponibilité de la structure et du processus de prestation de soins conservateurs par choix restreint (à savoir des soins conservateurs destinés aux patients pour lesquels des contraintes liées aux ressources empêchent ou limitent l'accès au TSR) pour les patients atteints d'une MRC de stade G5) :

> signifie dans 50 % ou plus des centres (hôpitaux ou cliniques) et > signifie dans moins de 50 % des centres (hôpitaux ou cliniques).

C7.5.9. Infrastructure pour documenter et partager les entretiens sur la planification préalable des soins, y compris les décisions concernant le lieu de prédilection des soins, le décès et la réanimation	○	○	○	○

C7.5. Veuillez indiquer la disponibilité de la structure et du processus de prestation de soins conservateurs par choix restreint (à savoir des soins conservateurs destinés aux patients pour lesquels des contraintes liées aux ressources empêchent ou limitent l'accès au TSR) pour les patients atteints d'une MRC de stade G5) :

> signifie dans 50 % ou plus des centres (hôpitaux ou cliniques) et > signifie dans moins de 50 % des centres (hôpitaux ou cliniques).

C7.5.10. Fourniture d'un soutien psychologique, social et spirituel	○	○	○	○

C7.5. Veuillez indiquer la disponibilité de la structure et du processus de prestation de soins conservateurs par choix restreint (à savoir des soins conservateurs destinés aux patients pour lesquels des contraintes liées aux ressources empêchent ou limitent l'accès au TSR) pour les patients atteints d'une MRC de stade G5) :

> signifie dans 50 % ou plus des centres (hôpitaux ou cliniques) et > signifie dans moins de 50 % des centres (hôpitaux ou cliniques).

C7.5.11. Formation des prestataires de soins à la gestion des symptômes	○	○	○	○

C7.5. Veuillez indiquer la disponibilité de la structure et du processus de prestation de soins conservateurs par choix restreint (à savoir des soins conservateurs destinés aux patients pour lesquels des contraintes liées aux ressources empêchent ou limitent l'accès au TSR) pour les patients atteints d'une MRC de stade G5) :

> signifie dans 50 % ou plus des centres (hôpitaux ou cliniques) et > signifie dans moins de 50 % des centres (hôpitaux ou cliniques).

C7.5.12. Formation des prestataires de soins à la planification préalable des soins	○	○	○	○

C7.5. Veuillez indiquer la disponibilité de la structure et du processus de prestation de soins conservateurs par choix restreint (à savoir des soins conservateurs destinés aux patients pour lesquels des contraintes liées aux ressources empêchent ou limitent l'accès au TSR) pour les patients atteints d'une MRC de stade G5) :

> signifie dans 50 % ou plus des centres (hôpitaux ou cliniques) et > signifie dans moins de 50 % des centres (hôpitaux ou cliniques).

C7.5.13. Collecte systématique de données sur le nombre de patients recevant des soins conservateurs par choix restreint et leurs résultats	○	○	○	○

C8. Abordabilité financière

C8.1. Quelle est la quote-part nationale moyenne (y compris les médicaments mais pas d'autre matériel connexe) des patients hémodialysés dans votre pays (c.-à-d. la part du coût du traitement payée directement par le patient) ?

- ◯ S.O. (indisponible dans mon pays)
- ◯ 0%
- ◯ 1-25%
- ◯ 26-50%
- ◯ 51-75%
- ◯ >75%
- ◯ 100%

C8.1.1. Cette part varie-t-elle selon les régions du pays ?

- ◯ Oui (veuillez expliquer ci-dessous)
- ◯ Non
- ◯ Autre (veuillez expliquer ci-dessous)

C8.1.1. Si oui, veuillez préciser

____

C8.1.1. Si autre, veuillez préciser

____

C8.1.2. Cette part varie-t-elle en fonction des caractéristiques des patients (par ex. âge, sexe, statut professionnel) ?

- ◯ Oui (veuillez expliquer ci-dessous)
- ◯ Non
- ◯ Autre (veuillez expliquer ci-dessous)

C8.1.2. Si oui, veuillez préciser

____

C8.1.2. Si autre, veuillez préciser

____

C8.1.3. Quelle part (moyenne nationale) de patients atteints d'insuffisance rénale (MRST) sous hémodialyse cesse-t-elle la dialyse dans l'année pour des raisons financières dans votre pays ?

- ◯ S.O. (indisponible dans mon pays)
- ◯ 0%
- ◯ 1-10%
- ◯ 11-25%
- ◯ 26-50%
- ◯ >50%

C8.2. Quelle est la quote-part nationale moyenne (y compris les médicaments mais pas d'autre matériel connexe) des patients sous dialyse péritonéale dans votre pays (c.-à-d. la part du coût du traitement payée directement par le patient) ?

- ◯ S.O. (indisponible dans mon pays)
- ◯ 0%
- ◯ 1-25%
- ◯ 26-50%
- ◯ 51-75%
- ◯ >75%
- ◯ 100%

C8.2.1. Cette part varie-t-elle selon les régions du pays ?

- ◯ Oui (veuillez expliquer ci-dessous)
- ◯ Non
- ◯ Autre (veuillez expliquer ci-dessous)

C8.2.1. Si oui, veuillez préciser

____

C8.2.1. Si autre, veuillez préciser

____

C8.2.2. Cette part varie-t-elle en fonction des caractéristiques des patients (par ex. âge, sexe, statut professionnel) ?

◯ Oui (veuillez expliquer ci-dessous)
◯ Non
◯ Autre (veuillez expliquer ci-dessous)

C8.2.2. Si oui, veuillez préciser

____

C8.2.2. Si autre, veuillez préciser

____

C8.3. Quelle est la quote-part nationale moyenne (y compris les médicaments mais pas d'autre matériel connexe) des patients transplantés rénaux dans votre pays (c.-à-d. la part du coût du traitement payée directement par le patient) ?

◯ S.O. (indisponible dans mon pays)
◯ 0%
◯ 1-25%
◯ 26-50%
◯ 51-75%
◯ >75%
◯ 100%

C8.3.1. Cette part varie-t-elle selon les régions du pays ?

◯ Oui (veuillez expliquer ci-dessous)
◯ Non
◯ Autre (veuillez expliquer ci-dessous)

C8.3.1. Si oui, veuillez préciser

____

C8.3.1. Si autre, veuillez préciser

____

C8.3.2. Cette part varie-t-elle en fonction des caractéristiques des patients (par ex. âge, sexe, statut professionnel) ?

◯ Oui (veuillez expliquer ci-dessous)
◯ Non
◯ Autre (veuillez expliquer ci-dessous)

C8.3.2. Si oui, veuillez préciser

____

C8.3.2. Si autre, veuillez préciser

____

C8.4. Quelle part (moyenne nationale) de patients atteints d'insuffisance rénale (MRST) peut-elle accéder à la dialyse dans votre pays ?

◯ S.O. (indisponible dans mon pays)
◯ 1-10%
◯ 11-25%
◯ 26-50%
◯ >50%

C8.4.1. Cette part varie-t-elle selon les régions du pays ?

◯ Oui (veuillez expliquer ci-dessous)
◯ Non
◯ Autre (veuillez expliquer ci-dessous)

C8.4.1. Si oui, veuillez préciser

____

C8.4.1. Si autre, veuillez préciser

____

C8.4.2. Cette part varie-t-elle en fonction des caractéristiques des patients (par ex. âge, sexe, statut professionnel) ?

- ◯ Oui (veuillez expliquer ci-dessous)
- ◯ Non
- ◯ Autre (veuillez expliquer ci-dessous)

C8.4.2. Si oui, veuillez préciser

____

C8.4.2. Si autre, veuillez préciser

____

C8.5. Parmi les patients de votre pays atteints d'insuffisance rénale (MRST) qui sont en mesure d'accéder à la dialyse, quelle part commence-t-elle généralement par la dialyse péritonéale ?

- ◯ S.O. - la dialyse (quelle qu'elle soit) n'est pas disponible dans mon pays
- ◯ 0 % (ce qui signifie que des patients peuvent accéder à une forme quelconque de dialyse, mais aucun ne commence par la DP)
- ◯ 1-10%
- ◯ 11-25%
- ◯ 26-50%
- ◯ >50%

C8.5.1. Cette part varie-t-elle selon les régions du pays ?

- ◯ Oui (veuillez expliquer ci-dessous)
- ◯ Non
- ◯ Autre (veuillez expliquer ci-dessous)

C8.5.1. Si oui, veuillez préciser

____

C8.5.1. Si autre, veuillez préciser

____

C8.5.2. Cette part varie-t-elle en fonction des caractéristiques des patients (par ex. âge, sexe, statut professionnel) ?

- ◯ Oui (veuillez expliquer ci-dessous)
- ◯ Non
- ◯ Autre (veuillez expliquer ci-dessous)

C8.5.2. Si oui, veuillez préciser

____

C8.5.2. Si autre, veuillez préciser

____

C8.6. Parmi les patients de votre pays atteints d'insuffisance rénale (MRST) qui sont admissibles à la transplantation, quelle part est-elle en mesure d'accéder à la transplantation rénale ?	◯ S.O. (indisponible dans mon pays)<br>◯ 1-10%<br>◯ 11-25%<br>◯ 26-50%<br>◯ >50%
C8.6.1. Cette part varie-t-elle selon les régions du pays ?	◯ Oui (veuillez expliquer ci-dessous)<br>◯ Non<br>◯ Autre (veuillez expliquer ci-dessous)
C8.6.1. Si oui, veuillez préciser	__________________________________
C8.6.1. Si autre, veuillez préciser	__________________________________
C8.6.2. Cette part varie-t-elle en fonction des caractéristiques des patients (par ex. âge, sexe, statut professionnel) ?	◯ Oui (veuillez expliquer ci-dessous)<br>◯ Non<br>◯ Autre (veuillez expliquer ci-dessous)
C8.6.2. Si oui, veuillez préciser	__________________________________
C8.6.2. Si autre, veuillez préciser	__________________________________

C9. Qualité de la dialyse péritonéale
Si la dialyse péritonéale est disponible dans votre pays, quelle proportion de centres mesure et rapporte systématiquement ce qui suit pour évaluer la qualité de la dialyse fournie ?

C9.1. Quelle part de patients sous DP commence-t-elle par une DP inférieure à la dose complète (c.-à-d. incrémentale) ? (< 8 l/jour est considéré comme inférieur à la dose complète)	◯ 0%<br>◯ 1-10%<br>◯ 11-25%<br>◯ 26-50%<br>◯ >50%
C9.2. Mesure des résultats rapportés par les patients (par ex. fatigue, qualité de vie, satisfaction, douleur) :	◯ 0% (aucun)<br>◯ 1-10% (peu)<br>◯ 11-50% (quelques-uns)<br>◯ 51-75% (la plupart)<br>◯ >75% (presque tous)
C9.3. Pression artérielle :	◯ 0% (aucun)<br>◯ 1-10% (peu)<br>◯ 11-50% (quelques-uns)<br>◯ 51-75% (la plupart)<br>◯ >75% (presque tous)
C9.4. Clairance des petits solutés (par ex. Kt/V ou clairance de la créatinine) :	◯ 0% (aucun)<br>◯ 1-10% (peu)<br>◯ 11-50% (quelques-uns)<br>◯ 51-75% (la plupart)<br>◯ >75% (presque tous)
C9.5. Hémoglobine/hématocrite :	◯ 0% (aucun)<br>◯ 1-10% (peu)<br>◯ 11-50% (quelques-uns)<br>◯ 51-75% (la plupart)<br>◯ >75% (presque tous)
C9.6. BMarqueurs minéraux osseux (calcium, phosphate, hormone parathyroïdienne [HPT]) :	◯ 0% (aucun)<br>◯ 1-10% (peu)<br>◯ 11-50% (quelques-uns)<br>◯ 51-75% (la plupart)<br>◯ >75% (presque tous)
C9.7. Survie technique (transfert en HD) :	◯ 0% (aucun)<br>◯ 1-10% (peu)<br>◯ 11-50% (quelques-uns)<br>◯ 51-75% (la plupart)<br>◯ >75% (presque tous)
C9.8. Survie du patient :	◯ 0% (aucun)<br>◯ 1-10% (peu)<br>◯ 11-50% (quelques-uns)<br>◯ 51-75% (la plupart)<br>◯ >75% (presque tous)
C9.9. Quel est le rapport personnel infirmier/patient par équipe dans ≥ 50 % des centres de dialyse péritonéale dans votre pays ?	◯ 1:1 à 1:3<br>◯ 1:4 à 1:6<br>◯ 1:7 à 1:9<br>◯ 1:10 à 1:13<br>◯ ≥1:14<br>◯ Inconnu

C10. Qualité de l'hémodialyse
Si l'hémodialyse est disponible dans votre pays, quelle proportion de centres mesure et rapporte systématiquement ce qui suit pour évaluer la qualité de la dialyse fournie ?

C10.1. Quelle part de patients HD commence-t-elle par une HD moins de 3 x/semaine (c.-à-d. HD incrémentale) ?	◯ 0%<br>◯ 1-10%<br>◯ 11-25%<br>◯ 26-50%<br>◯ >50%
C10.2. Mesure des résultats rapportés par les patients (par ex. fatigue, qualité de vie, satisfaction, douleur, etc.) :	◯ 0% (aucun)<br>◯ 1-10% (peu)<br>◯ 11-50% (quelques-uns)<br>◯ 51-75% (la plupart)<br>◯ >75% (presque tous)
C10.3. Pression artérielle :	◯ 0% (aucun)<br>◯ 1-10% (peu)<br>◯ 11-50% (quelques-uns)<br>◯ 51-75% (la plupart)<br>◯ >75% (presque tous)
C10.4. Clairance des petits solutés (par ex. Kt/V ou clairance de la créatinine) :	◯ 0% (aucun)<br>◯ 1-10% (peu)<br>◯ 11-50% (quelques-uns)<br>◯ 51-75% (la plupart)<br>◯ >75% (presque tous)
C10.5. Hémoglobine/hématocrite :	◯ 0% (aucun)<br>◯ 1-10% (peu)<br>◯ 11-50% (quelques-uns)<br>◯ 51-75% (la plupart)<br>◯ >75% (presque tous)
C10.6. Marqueurs minéraux osseux (calcium, phosphate, HPT) :	◯ 0% (aucun)<br>◯ 1-10% (peu)<br>◯ 11-50% (quelques-uns)<br>◯ 51-75% (la plupart)<br>◯ >75% (presque tous)
C10.7. Survie technique :	◯ 0% (aucun)<br>◯ 1-10% (peu)<br>◯ 11-50% (quelques-uns)<br>◯ 51-75% (la plupart)<br>◯ >75% (presque tous)
C10.8. Survie du patient :	◯ 0% (aucun)<br>◯ 1-10% (peu)<br>◯ 11-50% (quelques-uns)<br>◯ 51-75% (la plupart)<br>◯ >75% (presque tous)
C10.9. Surveillance des hépatites B et C et de la virologie du VIH au moins deux fois par an :	◯ 0% (aucun)<br>◯ 1-10% (peu)<br>◯ 11-50% (quelques-uns)<br>◯ 51-75% (la plupart)<br>◯ >75% (presque tous)

C10.10. Surveillance régulière de la qualité de l'eau de dialyse en ce qui concerne les bactéries et les composants chimiques, conformément à la recommandation de l'AAMI ou d'un organisme national de réglementation équivalent :	◯ 0% (aucun)<br>◯ 1-10% (peu)<br>◯ 11-50% (quelques-uns)<br>◯ 51-75% (la plupart)<br>◯ >75% (presque tous)
C10.11. Revue régulière des patients HD par un néphrologue au moins une fois tous les 3 mois :	◯ 0% (aucun)<br>◯ 1-10% (peu)<br>◯ 11-50% (quelques-uns)<br>◯ 51-75% (la plupart)<br>◯ >75% (presque tous)
C10.12. Quel est le rapport personnel infirmier/patient par équipe dans ≥ 50 % des centres d'hémodialyse dans votre pays ?	◯ 1:1 à 1:3<br>◯ 1:4 à 1:6<br>◯ 1:7 à 1:9<br>◯ 1:10 à 1:13<br>◯ ≥1:14<br>◯ Inconnu

C11. Qualité de la transplantation rénale

Si la transplantation rénale est disponible dans votre pays, quelle proportion de centres mesure et rapporte systématiquement ce qui suit pour évaluer la qualité de la transplantation fournie ?

C11.1. Mesure des résultats rapportés par les patients (par ex. fatigue, qualité de vie, satisfaction, douleur, etc.) :	◯ 0% (aucun)<br>◯ 1-10% (peu)<br>◯ 11-50% (quelques-uns)<br>◯ 51-75% (la plupart)<br>◯ >75% (presque tous)<br>◯ Inconnu
C11.2. Retard de reprise de la fonction rénale :	◯ 0% (aucun)<br>◯ 1-10% (peu)<br>◯ 11-50% (quelques-uns)<br>◯ 51-75% (la plupart)<br>◯ >75% (presque tous)<br>◯ Inconnu
C11.3. Taux de rejet :	◯ 0% (aucun)<br>◯ 1-10% (peu)<br>◯ 11-50% (quelques-uns)<br>◯ 51-75% (la plupart)<br>◯ >75% (presque tous)<br>◯ Inconnu
C11.4. Fonction de l'allogreffe rénale :	◯ 0% (aucun)<br>◯ 1-10% (peu)<br>◯ 11-50% (quelques-uns)<br>◯ 51-75% (la plupart)<br>◯ >75% (presque tous)<br>◯ Inconnu
C11.5. Survie du greffon :	◯ 0% (aucun)<br>◯ 1-10% (peu)<br>◯ 11-50% (quelques-uns)<br>◯ 51-75% (la plupart)<br>◯ >75% (presque tous)<br>◯ Inconnu
C11.6. Survie du patient :	◯ 0% (aucun)<br>◯ 1-10% (peu)<br>◯ 11-50% (quelques-uns)<br>◯ 51-75% (la plupart)<br>◯ >75% (presque tous)<br>◯ Inconnu

C12. Accès

C12.1. Concernant l'hémodialyse, quelle proportion de patients commence-t-elle systématiquement la dialyse avec un accès vasculaire fonctionnel (fistule ou greffon AV) ?	◯ 0% (aucun)<br>◯ 1-10% (peu)<br>◯ 11-50% (quelques-uns)<br>◯ 51-75% (la plupart)<br>◯ >75% (presque tous)<br>◯ Inconnu
C12.2. Concernant l'hémodialyse, quelle proportion de patients commence-t-elle systématiquement la dialyse avec un cathéter de dialyse tunnelisé ?	◯ 0% (aucun)<br>◯ 1-10% (peu)<br>◯ 11-50% (quelques-uns)<br>◯ 51-75% (la plupart)<br>◯ >75% (presque tous)<br>◯ Inconnu
C12.3. Concernant l'hémodialyse, quelle proportion de patients commence-t-elle généralement la dialyse avec un cathéter de dialyse temporaire ?	◯ 0% (aucun)<br>◯ 1-10% (peu)<br>◯ 11-50% (quelques-uns)<br>◯ 51-75% (la plupart)<br>◯ >75% (presque tous)<br>◯ Inconnu
C12.4. Concernant l'hémodialyse, quelle est la proportion de patients dialysés prévalents ayant un accès vasculaire fonctionnel (fistule ou greffon AV) ?	◯ 0% (aucun)<br>◯ 1-10% (peu)<br>◯ 11-50% (quelques-uns)<br>◯ 51-75% (la plupart)<br>◯ >75% (presque tous)<br>◯ Inconnu

Accès pour la dialyse entière - répondez uniquement si l'hémodialyse ou la dialyse péritonéale est disponible dans votre pays

C12.5. Concernant l'hémodialyse ou la dialyse péritonéale, quelle proportion de patients reçoit-elle systématiquement des informations sur les meilleurs moyens d'accès et la chirurgie en temps voulu (par ex. six mois avant le début de l'hémodialyse, un mois avant le début de la dialyse péritonéale) ?	◯ 0% (aucun)<br>◯ 1-10% (peu)<br>◯ 11-50% (quelques-uns)<br>◯ 51-75% (la plupart)<br>◯ >75% (presque tous)<br>◯ Inconnu

C13. Résultats (hémodialyse)

C13.1. Quelle proportion (moyenne nationale) de patients atteints d'insuffisance rénale (MRST) hémodialysés est-elle décédée au cours de la première année de dialyse (mortalité durant la première année) dans votre pays ?

- ◯ 1-10%
- ◯ 11-20%
- ◯ 21-30%
- ◯ 31-50%
- ◯ >50%
- ◯ Inconnu

C13.2. Quelle est la cause de décès la plus fréquente parmi les patients hémodialysés dans votre pays ?

- ◯ Maladie cardiovasculaire (cardiopathie ischémique, arythmie, maladie vasculaire cérébrale)
- ◯ Infection (infection liée à l'accès, FAV/GAV infectés, bactériémie liée au cathéter)
- ◯ Infection (autres sources, pneumonie, gangrène des membres, etc.)
- ◯ Tumeur maligne
- ◯ Arrêt de la dialyse (pour des raisons sociales)
- ◯ Arrêt de la dialyse (en raison du coût des soins)
- ◯ Autres (veuillez préciser)
- ◯ Inconnu

C13.2. Si autre, veuillez préciser

____

C13.3. Quelle proportion (moyenne nationale) de patients atteints d'insuffisance rénale (MRST) hémodialysés nécessite-t-elle au moins une hospitalisation au cours de la première année de dialyse (hospitalisation durant la première année) dans votre pays ?

- ◯ 1-10%
- ◯ 11-20%
- ◯ 21-30%
- ◯ 31-50%
- ◯ >50%
- ◯ Inconnu

C13.4. Quelle est la cause d'hospitalisation la plus fréquente parmi les patients hémodialysés dans votre pays ?

- ◯ Maladie cardiovasculaire (cardiopathie ischémique, arythmie, maladie vasculaire cérébrale)
- ◯ Dysfonctionnement de l'accès (dysfonctionnement de la FAV/du GAV ou cathéter veineux central obstrué)
- ◯ Infection liée à l'accès (FAV/GAV infectés, bactériémie liée au cathéter)
- ◯ Infection (autres sources, pneumonie, gangrène des membres, etc.)
- ◯ Autres (veuillez préciser)
- ◯ Inconnu

C13.4. Si autre, veuillez préciser

____

C14. Résultats (dialyse péritonéale)

C14.1. Quelle proportion (moyenne nationale) de patients atteints d'insuffisance rénale (MRST) sous dialyse péritonéale est-elle décédée au cours de la première année de dialyse (mortalité durant la première année) dans votre pays ?

- ◯ 1-10%
- ◯ 11-20%
- ◯ 21-30%
- ◯ 31-50%
- ◯ >50%
- ◯ Inconnu

C14.2. Quelle est la cause de décès la plus fréquente parmi les patients sous dialyse péritonéale dans votre pays ?

- ◯ Maladie cardiovasculaire (cardiopathie ischémique, arythmie, maladie vasculaire cérébrale)
- ◯ Infection liée à la DP (péritonite liée à la DP, infection du site de sortie ou du tunnel)
- ◯ Infection (autres sources, pneumonie, gangrène des membres, etc.)
- ◯ Tumeur maligne
- ◯ Arrêt de la dialyse (pour des raisons sociales)
- ◯ Arrêt de la dialyse (en raison du coût des soins
- ◯ Autres (veuillez préciser)
- ◯ Inconnu

C14.2. Si autre, veuillez préciser

____

C14.3 Quelle proportion (moyenne nationale) de patients atteints d'insuffisance rénale (MRST) sous dialyse péritonéale nécessite-t-elle au moins une hospitalisation au cours de la première année de dialyse (hospitalisation durant la première année) dans votre pays ?

- ◯ 1-10%
- ◯ 11-20%
- ◯ 21-30%
- ◯ 31-50%
- ◯ >50%
- ◯ Inconnu

C14.4. Quelle est la cause d'hospitalisation la plus fréquente parmi les patients sous dialyse péritonéale dans votre pays ?

- ◯ Maladie cardiovasculaire (cardiopathie ischémique, arythmie, maladie vasculaire cérébrale)
- ◯ Dysfonctionnement de l'accès (cathéter de DP obstrué, migration de l'embout du cathéter)
- ◯ Infection liée à la DP (péritonite, infection du site de sortie ou du tunnel)
- ◯ Infection (autres sources, pneumonie, gangrène des membres, etc.)
- ◯ Autres (veuillez préciser)
- ◯ Inconnu

C14.4. Si autre, veuillez préciser

____

C15. Données démographiques

C15.1. Quelle proportion (moyenne nationale) de patients atteints d'insuffisance rénale (MRST) est-elle âgée de plus de 65 ans dans votre pays ?

- ◯ 0%
- ◯ 1-10%
- ◯ 11-25%
- ◯ 26-50%
- ◯ >50%
- ◯ Inconnu

C15.2. Quelle proportion (moyenne nationale) de patients atteints d'insuffisance rénale (MRST) est-elle composée de femmes dans votre pays ?

- ◯ 0%
- ◯ 1-10%
- ◯ 11-25%
- ◯ 26-50%
- ◯ >50%
- ◯ Inconnu

C16. Étiologie de l'insuffisance rénale (MRST)

C16.1. Quelle est la cause la plus fréquente d'insuffisance rénale (MRST) dans votre pays ?

- ◯ Néphropathie diabétique
- ◯ Polykystose rénale
- ◯ Hypertension artérielle
- ◯ Glomérulonéphrite
- ◯ Autres (veuillez préciser)
- ◯ Inconnu

C16.1. Si autre, veuillez préciser

____

C16.2. Quelle proportion (moyenne nationale) de patients atteints d'insuffisance rénale (MRST) est-elle due à une néphropathie diabétique dans votre pays ?

- ◯ 0%
- ◯ 1-25%
- ◯ 26-50%
- ◯ 51-75%
- ◯ >75%
- ◯ Inconnu

C16.3. Quelle proportion (moyenne nationale) de patients atteints d'insuffisance rénale (MRST) est-elle due à une glomérulonéphrite dans votre pays ?

- ◯ 0%
- ◯ 1-25%
- ◯ 26-50%
- ◯ 51-75%
- ◯ >75%
- ◯ Inconnu

C16.4. Quelle proportion (moyenne nationale) de patients atteints d'insuffisance rénale (MRST) est-elle due à une polykystose rénale dans votre pays ?

- ◯ 0%
- ◯ 1-25%
- ◯ 26-50%
- ◯ 51-75%
- ◯ >75%
- ◯ Inconnu

C16.5 Quelle proportion (moyenne nationale) de patients atteints d'insuffisance rénale (MRST) est-elle due à une néphropathie hypertensive dans votre pays ?

- ◯ 0%
- ◯ 1-25%
- ◯ 26-50%
- ◯ 51-75%
- ◯ >75%
- ◯ Inconnu

C17 Utilisation de la technologie dans les soins rénaux

C17.1. Existe-t-il une capacité pour les examens de télésanté/télénéphrologie dans la gestion de la MRC et de l'insuffisance rénale (MRST) dans votre pays ?

- ◯ Oui (si possible, veuillez préciser brièvement)
- ◯ Non
- ◯ Inconnu

C17.1. Si oui, veuillez préciser

____

C17.2. Les patients ont-ils la possibilité de recevoir des communications (c.-à-d. des résultats de tests, des rappels de rendez-vous) de la part des cliniques/hôpitaux par texto (SMS) ou par e-mail ?

- ◯ Oui (si possible, veuillez préciser brièvement)
- ◯ Non
- ◯ Inconnu

C17.2. Si oui, veuillez préciser

____

C17.3.Existe-t-il un financement/remboursement pour les prestataires qui fournissent des examens à distance de télésanté/télénéphrologie dans la MRC et l'insuffisance rénale (MRST) ?

- ◯ Financement public par le gouvernement et gratuit au lieu d'administration
- ◯ Financement public par le gouvernement, mais certains frais au lieu d'administration
- ◯ Combinaison d'un système financé par des fonds publics (que la composante financée par des fonds publics soit gratuite ou pas au lieu d'administration) et d'un système privé (veuillez expliquer)
- ◯ Uniquement privé et payant
- ◯ Uniquement privé via les compagnies d'assurance maladie
- ◯ Différents systèmes - programmes fournis par le gouvernement, des ONG et des communautés
- ◯ S.O. (la télésanté/télénéphrologie n'est pas disponible dans mon pays)
- ◯ Autre (veuillez préciser)

C17.3. Si autre, veuillez préciser

____

C17.3. S'il s'agit d'un mélange de fonds publics et privés, veuillez expliquer.

____

C18 Gestion des catastrophes/Populations vulnérables

C18.1.Existe-t-il des directives relatives aux mesures à prendre en vue de la préparation aux catastrophes (à savoir dans les installations de dialyse) en cas de séisme, inondation ou sécheresse dans votre pays ?

- ◯ Oui (si possible, veuillez préciser brièvement)
- ◯ Non
- ◯ Inconnu

C18.1. Si oui, veuillez préciser

____

C18.2. Votre pays dispose-t-il d'un représentant au sein de la Renal Disaster Relief Task Force ?

- ◯ Oui (si possible, veuillez préciser brièvement)
- ◯ Non
- ◯ Inconnu

C18.2. Si oui, veuillez préciser

____

C18.3. Existe-t-il des moyens d'identifier les populations vulnérables (c.-à-d. les personnes en précarité de logement, les minorités raciales/ethniques, les personnes vivant dans la pauvreté, les personnes en insécurité alimentaire) dans votre pays ?

- ◯ Oui (si possible, veuillez préciser brièvement)
- ◯ Non
- ◯ Inconnu

C18.3. Si oui, veuillez préciser

____

C18.4. Qu'est-ce qui décrit le mieux la structure de financement de votre système de soins de santé en ce qui concerne le traitement de la MRC et de l'insuffisance rénale (MRST) chez les populations de réfugiés ?

Réduisez la taille de la police si le texte ci-dessous se chevauche.

	Financement public par le gouvernement et gratuit au lieu d'administration	Financement public par le gouvernement, mais certains frais au lieu d'administration	Combinaison d'un système financé par des fonds publics (que la composante financée par des fonds publics soit gratuite ou pas au lieu d'administration) et d'un système privé (veuillez expliquer)	Uniquement privé et payant	Uniquement privé via les compagnies d'assurance maladie	Différents systèmes - programmes fournis par le gouvernement, des Organisations Non Gouvernementales (ONGs) et des communautés	Autre (veuillez préciser)	S.O. (les réfugiés n'ont pas systématiquement accès au traitement de la maladie rénale)
C18.4.1. Hémodialyse (en tout ou en partie)	○	○	○	○	○	○	○	○

C18.4. Qu'est-ce qui décrit le mieux la structure de financement de votre système de soins de santé en ce qui concerne le traitement de la MRC et de l'insuffisance rénale (MRST) chez les populations de réfugiés ?

Réduisez la taille de la police si le texte ci-dessous se chevauche.

C18.4.2. Dialyse péritonéale (en tout ou en partie)	○	○	○	○	○	○	○	○

C18.4. Qu'est-ce qui décrit le mieux la structure de financement de votre système de soins de santé en ce qui concerne le traitement de la MRC et de l'insuffisance rénale (MRST) chez les populations de réfugiés ?

Réduisez la taille de la police si le texte ci-dessous se chevauche.

C18.4.3. Transplantation rénale (en tout ou en partie)	○	○	○	○	○	○	○	○

C18.4. Qu'est-ce qui décrit le mieux la structure de financement de votre système de soins de santé en ce qui concerne le traitement de la MRC et de l'insuffisance rénale (MRST) chez les populations de réfugiés ?

Réduisez la taille de la police si le texte ci-dessous se chevauche.

C18.4.4. Soins conservateurs (en tout ou en partie) ○ ○ ○ ○ ○ ○ ○ ○

C18.4.1. Si autre, veuillez préciser __________________________________

C18.4.1. S'il s'agit d'un mélange de fonds publics et privés, veuillez expliquer. __________________________________

C18.4.2. Si autre, veuillez préciser __________________________________

C18.4.2. S'il s'agit d'un mélange de fonds publics et privés, veuillez expliquer. __________________________________

C18.4.3. Si autre, veuillez préciser __________________________________

C18.4.3. S'il s'agit d'un mélange de fonds publics et privés, veuillez expliquer. __________________________________

C18.4.4. Si autre, veuillez préciser __________________________________

C18.4.4. S'il s'agit d'un mélange de fonds publics et privés, veuillez expliquer. __________________________________

D. Systèmes d'information et statistiques de santé
D1. Registres

Définitions/abréviations

Registre : collecte systématique de données visant à évaluer des résultats spécifiés pour une population définie, afin de servir un ou plusieurs objectifs scientifiques, cliniques ou politiques prédéterminés.

LRA : lésion rénale aiguë

D1.1. Pour quelles affections ou quels traitements existe-t-il un registre > dans votre pays ?

	Oui	Non	Inconnu
D1.1.1. MRC (sans TSR)	○	○	○

D. Systèmes d'information et statistiques de santé
D1. Registres

Définitions/abréviations

Registre : collecte systématique de données visant à évaluer des résultats spécifiés pour une population définie, afin de servir un ou plusieurs objectifs scientifiques, cliniques ou politiques prédéterminés.

LRA : lésion rénale aiguë

D1.1. Pour quelles affections ou quels traitements existe-t-il un registre > dans votre pays ?

D1.1.2. Dialyse ○ ○ ○

D. Systèmes d'information et statistiques de santé
D1. Registres

Définitions/abréviations

Registre : collecte systématique de données visant à évaluer des résultats spécifiés pour une population définie, afin de servir un ou plusieurs objectifs scientifiques, cliniques ou politiques prédéterminés.

LRA : lésion rénale aiguë

D1.1. Pour quelles affections ou quels traitements existe-t-il un registre > dans votre pays ?

D1.1.3. Transplantation	○	○	○

D. Systèmes d'information et statistiques de santé
D1. Registres

Définitions/abréviations

Registre : collecte systématique de données visant à évaluer des résultats spécifiés pour une population définie, afin de servir un ou plusieurs objectifs scientifiques, cliniques ou politiques prédéterminés.

LRA : lésion rénale aiguë

D1.1. Pour quelles affections ou quels traitements existe-t-il un registre > dans votre pays ?

D1.1.4. LRA ○ ○ ○

D. Systèmes d'information et statistiques de santé
D1. Registres

Définitions/abréviations

Registre : collecte systématique de données visant à évaluer des résultats spécifiés pour une population définie, afin de servir un ou plusieurs objectifs scientifiques, cliniques ou politiques prédéterminés.

LRA : lésion rénale aiguë

D1.1. Pour quelles affections ou quels traitements existe-t-il un registre > dans votre pays ?

D1.1.5. Soins conservateurs ◯ ◯ ◯

D1.2. S'il existe un registre de la MRC pour les patients qui ne nécessitent pas de TSR, quelle est la base de la participation à ce registre ?
- ◯ Volontaire
- ◯ Obligatoire
- ◯ Inconnu

D1.3. S'il existe un registre de la MRC pour les patients qui ne nécessitent pas de TSR, quelle est la couverture géographique de ce registre ? (Veuillez cocher toutes les réponses qui s'appliquent.)
- ☐ Nationale
- ☐ Régionale/départementale/provinciale
- ☐ Locale/hospitalière/communautaire

D1.4. S'il existe un registre de la MRC pour les patients qui ne nécessitent pas de TSR, que couvre-t-il ? (Veuillez cocher toutes les réponses qui s'appliquent.)
- ◯ Tout le spectre de la MRC (stades 1 à 5)
- ◯ MRC avancée uniquement (stades 4/5)

D1.5. S'il existe un registre de la dialyse, quelle est la base de la participation à ce registre ?
- ◯ Volontaire
- ◯ Obligatoire
- ◯ Inconnu

D1.6. S'il existe un registre de la dialyse, quelle est la couverture géographique de ce registre ? (Veuillez cocher toutes les réponses qui s'appliquent.)
- ☐ Nationale
- ☐ Régionale/départementale/provinciale
- ☐ Locale/hospitalière/communautaire

D1.7. S'il existe un registre de la dialyse, quelles informations collecte-t-il ? (Veuillez cocher toutes les réponses qui s'appliquent.)
- ☐ Étiologie de l'insuffisance rénale (MRST
- ☐ Modalité de la dialyse
- ☐ Prescription de la dialyse
- ☐ Accès de la dialyse (par ex. accès vasculaire pour l'HD, cathéter de DP)
- ☐ Mesures basées sur le processus (par ex. anémie, maladie osseuse, marqueurs de contrôle de la PA)
- ☐ Mesures des résultats des patients (par ex. hospitalisations)
- ☐ Mesures des résultats des patients (par ex. satisfaction, qualité de vie)
- ☐ Mesures des résultats des patients (par ex. mortalité)

D1.8. S'il existe un registre des transplantations, quelle est la base de la participation à ce registre ?	◯ Volontaire<br>◯ Obligatoire<br>◯ Inconnu
D1.9.S'il existe un registre des transplantations, quelle est la couverture géographique de ce registre ? (Veuillez cocher toutes les réponses qui s'appliquent.)	☐ Nationale<br>☐ Régionale/départementale/provinciale<br>☐ Locale/hospitalière/communautaire
D1.10. S'il existe un registre des transplantations, quelles informations collecte-t-il ? (Veuillez cocher toutes les réponses qui s'appliquent.)	☐ Étiologie de l'insuffisance rénale (MRST)<br>☐ Source de l'organe transplanté (donneur décédé/vivant)<br>☐ Type d'immunosuppression<br>☐ Épisodes de rejet<br>☐ Types et épisodes d'infection<br>☐ Mesures des résultats des patients (par ex. hospitalisations)<br>☐ Mesures des résultats des patients (par ex. satisfaction, qualité de vie)<br>☐ Mesures des résultats des patients (par ex. mortalité)
D1.11. S'il existe un registre des LRA, quelle est la base de la participation à ce registre ?	◯ Volontaire<br>◯ Obligatoire<br>◯ Inconnu
D1.12. S'il existe un registre des LRA, quelle est la couverture géographique de ce registre ? (Veuillez cocher toutes les réponses qui s'appliquent.)	☐ Nationale<br>☐ Régionale/départementale/provinciale<br>☐ Locale/hospitalière/communautaire
D1.13. S'il existe un registre des LRA, que couvre-t-il ? (Veuillez cocher toutes les réponses qui s'appliquent.)	◯ Tout le spectre des LRA (stades 1 à 3)<br>◯ Les LRA nécessitant un traitement de suppléance rénale
D1.14. S'il existe un registre des LRA, quelles informations collecte-t-il ? (Veuillez cocher toutes les réponses qui s'appliquent.)	☐ Facteurs de risque de LRA<br>☐ Étiologie des LRA<br>☐ Incidence des LRA<br>☐ Mesures des résultats des patients (hospitalisations)<br>☐ Mesures des résultats des patients (nécessité d'un TSR, par ex. dialyse ou dialyse lente comme TCSR)<br>☐ Mesures des résultats des patients (mortalité)

D2. Identification de la maladie (LRA et MRC)
Définitions:

Directives: plan d'action recommandé basé sur des preuves, en vue de la prévention ou de la gestion de la maladie.

Identification: mesures exécutées parmi les populations à risque afin de diagnostiquer les personnes qui présentent des facteurs de risque ou un stade précoce de la maladie mais éventuellement pas encore de symptômes.

Politique: décision officielle spécifique ou ensemble de décisions conçues pour mener à bien un plan d'action approuvé par un organisme gouvernemental, y compris un ensemble d'objectifs, de priorités et de grandes orientations pour atteindre ces objectifs. Le document de politique peut inclure une stratégie pour donner effet à la politique.

Programme: ensemble planifié d'activités ou de procédures visant un objectif spécifique.

D2.1. Pour lesquels des groupes à haut risque suivants les praticiens de votre pays proposent-ils systématiquement des tests de la MRC ? (Veuillez cocher toutes les réponses qui s'appliquent.)

- ☐ Personnes hypertendues
- ☐ Diabétiques
- ☐ Personnes atteintes d'une maladie cardiovasculaire (cardiopathie ischémique, accident vasculaire cérébral, maladie vasculaire périphérique, insuffisance cardiaque)
- ☐ Personnes atteintes d'une maladie auto-immune/multisystémique (lupus érythémateux disséminé, polyarthrite rhumatoïde)
- ☐ Personnes âgées
- ☐ Personnes atteintes de troubles urologiques (maladies lithiasiques, structurelles)
- ☐ Utilisateurs chroniques de médicaments néphrotoxiques
- ☐ Membres de groupes ethniques à haut risque (aborigènes, africains, indo-asiatiques)
- ☐ Personnes ayant des antécédents familiaux de MRC
- ☐ S.O. - tests de routine de la MRC non proposés

D2.2. Dans votre pays, certains groupes ethniques sont-ils considérés comme présentant un risque accru de MRC ?

- ◯ Oui (veuillez préciser ci-dessous)
- ◯ Non
- ◯ Inconnu

D2.2. Si oui, veuillez préciser

____

D2.3. Dans votre pays, existe-t-il un programme de détection de la MRC basé sur une politique ou des directives nationales ?

- ◯ Oui
- ◯ Non
- ◯ Inconnu

D2.3.1. S'il existe un programme, comment est-il mis en œuvre (veuillez cocher toutes les réponses qui s'appliquent) ?	◯ Approche réactive - cas gérés à mesure qu'ils sont identifiés dans la pratique<br>◯ Dépistage actif de la population à risque lors de consultations médicales de routine<br>◯ Dépistage actif de la population à risque au moyen de processus de dépistage spécifiques<br>◯ Autre (veuillez préciser)
D2.3.1 Si autre, veuillez préciser	__________________________________
D2.4. Dans votre pays, certains groupes sont-ils considérés comme présentant un risque accru de LRA ?	◯ Oui (veuillez préciser ci-dessous)<br>◯ Non<br>◯ Inconnu
D2.4. Si oui, veuillez préciser	__________________________________
D2.5. Dans votre pays, existe-t-il un programme de détection des LRA basé sur une politique ou des directives nationales ?	◯ Oui<br>◯ Non<br>◯ Inconnu
D2.5.1. S'il existe un programme de détection des LRA, comment est-il mis en œuvre ? (Veuillez cocher toutes les réponses qui s'appliquent.)	◯ Approche réactive - cas gérés à mesure qu'ils sont identifiés par la pratique<br>◯ Dépistage actif de la population à risque lors de consultations médicales de routine<br>◯ Dépistage actif de la population à risque au moyen de processus de dépistage spécifiques<br>◯ Calcul automatisé par des systèmes médicaux générant des alertes électroniques<br>◯ Autre (veuillez préciser)
D2.5.1. Si autre, veuillez préciser	__________________________________
D3. Disposez-vous de mécanismes pour garantir la validité et la qualité des données contenues dans les systèmes d'information de santé ?	◯ Oui<br>◯ Non<br>◯ Inconnu

D4. Capacité d'identification et de gestion de la MRC

D4.1. Indiquez la disponibilité des services suivants dans le cadre de la surveillance et de la gestion de la MRC au niveau des soins PRIMAIRES dans votre pays.

	Disponible	Indisponible
D4.1.1. Mesure de la pression artérielle	○	○

D4. Capacité d'identification et de gestion de la MRC

D4.1. Indiquez la disponibilité des services suivants dans le cadre de la surveillance et de la gestion de la MRC au niveau des soins PRIMAIRES dans votre pays.

D4.1.2. Mesure de la taille et du poids pour calculer l'indice de masse corporelle	○	○

D4. Capacité d'identification et de gestion de la MRC

D4.1. Indiquez la disponibilité des services suivants dans le cadre de la surveillance et de la gestion de la MRC au niveau des soins PRIMAIRES dans votre pays.

D4.1.3. Mesure de la glycémie	○	○

D4. Capacité d'identification et de gestion de la MRC

D4.1. Indiquez la disponibilité des services suivants dans le cadre de la surveillance et de la gestion de la MRC au niveau des soins PRIMAIRES dans votre pays.

D4.1.4. Mesure de l'HbA1C	○	○

D4. Capacité d'identification et de gestion de la MRC

D4.1. Indiquez la disponibilité des services suivants dans le cadre de la surveillance et de la gestion de la MRC au niveau des soins PRIMAIRES dans votre pays.

D4.1.5. Mesure de la cholestérolémie	○	○

D4. Capacité d'identification et de gestion de la MRC

D4.1. Indiquez la disponibilité des services suivants dans le cadre de la surveillance et de la gestion de la MRC au niveau des soins PRIMAIRES dans votre pays.

D4.1.6. Mesure de la créatininémie sans rapport automatisé du DFGe	○	○

D4. Capacité d'identification et de gestion de la MRC

D4.1. Indiquez la disponibilité des services suivants dans le cadre de la surveillance et de la gestion de la MRC au niveau des soins PRIMAIRES dans votre pays.

D4.1.7. Mesure de la créatininémie avec rapport automatisé du DFGe	○	○

D4. Capacité d'identification et de gestion de la MRC

D4.1. Indiquez la disponibilité des services suivants dans le cadre de la surveillance et de la gestion de la MRC au niveau des soins PRIMAIRES dans votre pays.

D4.1.8. Analyse d'urine à l'aide de bandelettes réactives pour l'albumine/les protéines (tests qualitatifs)	○	○

D4. Capacité d'identification et de gestion de la MRC

D4.1. Indiquez la disponibilité des services suivants dans le cadre de la surveillance et de la gestion de la MRC au niveau des soins PRIMAIRES dans votre pays.

D4.1.9. Analyse d'urine à l'aide de bandelettes réactives pour l'albumine/les protéines (tests quantitatifs)	○	○

D4. Capacité d'identification et de gestion de la MRC

D4.1. Indiquez la disponibilité des services suivants dans le cadre de la surveillance et de la gestion de la MRC au niveau des soins PRIMAIRES dans votre pays.

D4.1.10. Albumine urinaire: taux de créatinine (RAC) ou protéines: mesures de la créatinine (PCR)	○	○

D4.2. Indiquez la disponibilité des services suivants dans le cadre de la surveillance et de la gestion de la MRC au niveau des soins SECONDAIRES OU TERTIAIRES dans votre pays.

	Disponible	Indisponible
D4.2.1. Mesure de la pression artérielle	○	○

D4.2. Indiquez la disponibilité des services suivants dans le cadre de la surveillance et de la gestion de la MRC au niveau des soins SECONDAIRES OU TERTIAIRES dans votre pays.

D4.2.2. Mesure de la taille et du poids pour calculer l'indice de masse corporelle	○	○

D4.2. Indiquez la disponibilité des services suivants dans le cadre de la surveillance et de la gestion de la MRC au niveau des soins SECONDAIRES OU TERTIAIRES dans votre pays.

D4.2.3. Mesure de la glycémie	○	○

D4.2. Indiquez la disponibilité des services suivants dans le cadre de la surveillance et de la gestion de la MRC au niveau des soins SECONDAIRES OU TERTIAIRES dans votre pays.

D4.2.4. Mesure de l'HbA1C	○	○

**D4.2. Indiquez la disponibilité des services suivants dans le cadre de la surveillance et de la gestion de la MRC au niveau des soins SECONDAIRES OU TERTIAIRES dans votre pays.**		
D4.2.5. Mesure de la cholestérolémie	○	○

D4.2. Indiquez la disponibilité des services suivants dans le cadre de la surveillance et de la gestion de la MRC au niveau des soins SECONDAIRES OU TERTIAIRES dans votre pays.

D4.2.6. Mesure de la créatininémie sans rapport automatisé du DFGe	○	○

D4.2. Indiquez la disponibilité des services suivants dans le cadre de la surveillance et de la gestion de la MRC au niveau des soins SECONDAIRES OU TERTIAIRES dans votre pays.

D4.2.7. Mesure de la créatininémie avec rapport automatisé du DFGe	○	○

D4.2. Indiquez la disponibilité des services suivants dans le cadre de la surveillance et de la gestion de la MRC au niveau des soins SECONDAIRES OU TERTIAIRES dans votre pays.

D4.2.8. Analyse d'urine à l'aide de bandelettes réactives pour l'albumine/les protéines (tests qualitatifs)	○	○

D4.2. Indiquez la disponibilité des services suivants dans le cadre de la surveillance et de la gestion de la MRC au niveau des soins SECONDAIRES OU TERTIAIRES dans votre pays.

D4.2.9. Analyse d'urine à l'aide de bandelettes réactives pour l'albumine/les protéines (tests quantitatifs)	○	○

D4.2. Indiquez la disponibilité des services suivants dans le cadre de la surveillance et de la gestion de la MRC au niveau des soins SECONDAIRES OU TERTIAIRES dans votre pays.

D4.2.10. Albumine urinaire: taux de créatinine (RAC) ou protéines: mesures de la créatinine (PCR)	○	○

D4.2. Indiquez la disponibilité des services suivants dans le cadre de la surveillance et de la gestion de la MRC au niveau des soins SECONDAIRES OU TERTIAIRES dans votre pays.

D4.2.11. Services radiologiques (par ex. équipements d'échographie rénale)	○	○

D4.2. Indiquez la disponibilité des services suivants dans le cadre de la surveillance et de la gestion de la MRC au niveau des soins SECONDAIRES OU TERTIAIRES dans votre pays.

D4.2.12. Services de pathologie (équipements d'interprétation de biopsie rénale)	○	○

D5: MRC d'origine inconnue et populations affectées de manière disproportionnée par la MRC

Cette rubrique a pour but de comprendre s'il existe des foyers régionaux de maladie rénale (MRC d'origine inconnue, en particulier) dans votre pays.

D.5.1. Suspectez-vous l'existence de variations régionales du taux de maladie rénale dans votre pays ? (Nous entendons par là des groupes de la population présentant un risque élevé d'insuffisance rénale nécessitant une dialyse ou une transplantation ou des personnes décédant d'une insuffisance rénale.)	◯ Oui (veuillez préciser)<br>◯ Non
D5.1. Si oui, veuillez préciser	______________________
D.5.2. Catégories d'âge touchées (sélectionnez toutes les réponses qui s'appliquent):	☐ < 18 ans<br>☐ 18-44 ans<br>☐ 45-64 ans<br>☐ 65 ans et plus
D.5.3. Selon vous, quelles sont certaines des causes ou certains des facteurs contributifs probables de la maladie rénale dans ces régions (sélectionnez toutes les réponses qui s'appliquent) ?	☐ Diabète<br>☐ Obésité<br>☐ Hypertension artérielle<br>☐ Environnement (par ex. eau, sol)<br>☐ Climat (par ex., température élevée)<br>☐ Génétique<br>☐ Biologie (par ex. forte prévalence du VIH ou de la tuberculose, calculs)<br>☐ Culture (par ex. alimentation, utilisation d'AINS, plantes aromatiques)<br>☐ Autre (précisez)
D.5.3. Si autre, veuillez préciser	______________________
D.5.4. Types d'industrie dans cette région (sélectionnez toutes les réponses qui s'appliquent):	☐ Agriculture<br>☐ Manufactures<br>☐ Exploitation minière<br>☐ Tourisme<br>☐ Services/professionnel<br>☐ Autre (précisez)
D.5.4. Si autre, veuillez préciser	______________________
D.5.5. Type de climat prédominant dans le pays:	◯ Tropical<br>◯ Semi-aride<br>◯ Tempéré<br>◯ Autre (précisez)
D.5.5. Si autre, veuillez préciser	______________________

D.5.6. Quelle est l'altitude du pays ?	◯ Élevée<br>◯ Au niveau de la mer<br>◯ Basse<br>◯ Autre (veuillez préciser)
D.5.6. Si autre, veuillez préciser	__________________________________

E. Politique nationale de santé

Définitions :
Politique : décision officielle spécifique ou ensemble de décisions conçues pour mener à bien un plan d'action approuvé par un organisme gouvernemental, y compris un ensemble d'objectifs, de priorités et de grandes orientations pour atteindre ces objectifs. Le document de politique peut inclure une stratégie pour donner effet à la politique.
Programme : ensemble planifié d'activités ou de procédures visant un objectif spécifique.
Stratégie : plan à long terme conçu pour atteindre un objectif particulier.

E1. Politique et stratégie
Maladies non transmissibles (MNT) : maladies qui ne peuvent pas être transmises d'une personne à une autre, notamment les maladies cardiovasculaires (comme les crises cardiaques et les accidents vasculaires cérébraux), les cancers, les maladies respiratoires chroniques (comme la bronchopneumopathie chronique obstructive et l'asthme) et le diabète.

E1.1. Votre pays dispose-t-il d'une stratégie nationale en matière de maladies non transmissibles ?	◯ Oui, en place (veuillez préciser)<br>◯ En cours de développement mais pas encore en place (veuillez préciser ci-dessous)<br>◯ Non<br>◯ Inconnu
E1.1. Si oui, veuillez préciser	______________________________
E1.1. Si en cours de développement, veuillez fournir des détails.	______________________________
E1.2. Votre pays dispose-t-il d'une stratégie nationale d'amélioration des soins des patients atteints d'une MRC ?	◯ Oui, il existe une stratégie nationale spécifique à la MRC<br>◯ Oui, mais la stratégie concernant la MRC est intégrée dans une stratégie relative aux MNT qui inclut d'autres maladies.<br>◯ Non<br>◯ Inconnu
E1.2.1. Veuillez sélectionner les populations couvertes par la stratégie nationale spécifique à la MRC (cochez toutes les réponses qui s'appliquent).	☐ MRC non dépendante à la dialyse<br>☐ Dialyse chronique<br>☐ Transplantation rénale
E1.2.2. Veuillez sélectionner les populations couvertes par la stratégie nationale générale en matière de MNT (cochez toutes les réponses qui s'appliquent).	☐ MRC non dépendante à la dialyse<br>☐ Dialyse chronique<br>☐ Transplantation rénale
E1.3. Des politiques spécifiques à la MRC sont-elles disponibles ?	◯ Oui<br>◯ Non<br>◯ Inconnu

E1.3.1. Si oui, veuillez préciser quels types de politique spécifique à la MRC sont disponibles dans votre pays (cochez toutes les réponses qui s'appliquent).	☐ Politiques nationales<br>☐ Politiques régionales

E2. Défense

E2.1. Selon vous, la MRC est-elle reconnue comme une priorité sanitaire par le gouvernement de votre pays ?

◯ Oui (veuillez préciser ci-dessous)
◯ Non (veuillez expliquer pourquoi ci-dessous)

E2.1. Si oui, veuillez préciser

____

E2.1. Si non, veuillez préciser

____

E2.2. Existe-t-il un groupe de défense aux niveaux supérieurs du gouvernement (par ex. une commission parlementaire) ou une ONG pour accroître la visibilité de la MRC et de sa prévention ?

◯ Oui (veuillez préciser ci-dessous)
◯ Non (veuillez expliquer pourquoi ci-dessous)
◯ Inconnu

E2.2. Si oui, veuillez préciser

____

E2.2. Si non, veuillez préciser

____

E2.3. Selon vous, les LRA et/ou leur prévention sont-elles reconnues comme une priorité sanitaire par le gouvernement de votre pays ?

◯ Oui (veuillez préciser ci-dessous)
◯ Non (veuillez expliquer pourquoi ci-dessous)

E2.3. Si oui, veuillez préciser

____

E2.3. Si non, veuillez préciser

____

E2.4. Existe-t-il un groupe de défense aux niveaux supérieurs du gouvernement (par ex. une commission parlementaire) ou une ONG pour accroître la visibilité des LRA et de leur prévention ?

◯ Oui (veuillez préciser ci-dessous)
◯ Non (veuillez expliquer pourquoi ci-dessous)
◯ Inconnu

E2.4. Si oui, veuillez préciser

____

E2.4. Si non, veuillez préciser

____

E2.5. Selon vous, l'insuffisance rénale (MRST) et/ou son traitement par TSR sont-ils reconnus comme une priorité sanitaire par le gouvernement de votre pays ?

◯ Oui (veuillez préciser ci-dessous)
◯ Non (veuillez expliquer pourquoi ci-dessous)

E2.5. Si oui, veuillez préciser

____

E2.5. Si non, veuillez préciser

____

E2.6. Existe-t-il un groupe de défense aux niveaux supérieurs du gouvernement (par ex. une commission parlementaire) ou une ONG pour accroître la visibilité de l'insuffisance rénale (MRST)/du TSR ?

○ Oui (veuillez préciser ci-dessous)
○ Non (veuillez expliquer pourquoi ci-dessous)
○ Inconnu

E2.6. Si oui, veuillez préciser

____

E2.6. Si non, veuillez préciser

____

E2.7. Existe-t-il des organisations nationales/régionales tournées vers les médecins ou des organisations de patients qui fournissent des ressources en matière de soins de l'insuffisance rénale (MRST) ?

○ Oui (veuillez préciser ci-dessous)
○ Non (veuillez expliquer pourquoi ci-dessous)
○ Inconnu

E2.7. Si oui, veuillez préciser

____

E2.7. Si non, veuillez préciser

____

E3. Obstacles à des soins rénaux optimaux

E3.1. Existe-t-il des obstacles spécifiques aux soins rénaux optimaux dans votre pays ? Veuillez cocher toutes les réponses qui s'appliquent.

- ☐ Géographie (distance par rapport aux soins ou temps de trajet long)
- ☐ Médecin (disponibilité, accès, connaissances, attitude)
- ☐ Patient (connaissances, attitude)
- ☐ Néphrologue (disponibilité)
- ☐ Système de soins de santé (disponibilité, accès, capacité)
- ☐ Manque de volonté politique et de politiques favorables
- ☐ Facteurs économiques (financement limité, mécanismes de remboursement défaillants)
- ☐ Autre (veuillez préciser)

E3.1. Si autre, veuillez préciser

____

E4. Comment avez-vous réuni les informations nécessaires pour répondre à cette enquête ? Veuillez cocher toutes les réponses qui s'appliquent.

- ☐ Opinion/connaissances personnelles
- ☐ Collecte d'informations auprès d'autres sources (par ex. littérature publiée ou rapports)
- ☐ Consultation d'autres collègues
- ☐ Autre (veuillez préciser)

E4. Si autre, veuillez préciser

____

Encuesta de la Sociedad Internacional de Nefrología (ISN) para el Atlas Mundial de Salud Renal (ISN-GKHA)

Please complete the survey below.

Thank you!

Encuesta de la Sociedad Internacional de Nefrología (ISN) para el Atlas Mundial de Salud Renal (ISN-GKHA)

Encuesta temática:

Evaluación del estado global de la asistencia renal: capacidad, disponibilidad, accesibilidad, asequibilidad y resultados

Perfiles nacionales de la evaluación global de la a la enfermedad renal: capacidad, disponibilidad, accesibilidad, asequibilidad y resultados

La Sociedad Internacional de Nefrología (ISN) trabaja en colaboración con las organizaciones e iniciativas a nivel tanto internacional como nacional para fomentar la detección precoz y el tratamiento eficaz de las enfermedades renales a fin de mejorar la salud y la calidad de la vida de los pacientes. Al intentar comprender y dar forma a las políticas sanitarias, prácticas e infraestructuras pertinentes, la ISN pretende facilitar la aplicación de una atención ética y equitativa para los enfermos renales de todas las regiones y países del mundo.

El Atlas Global de Salud Renal de la ISN, 2017 (1a edición) y 2019 (2a edición), publica los resultados de investigaciones sobre el estado de la atención a pacientes renales en todos los países del mundo.

Con el GKHA de la ISN se constató la existencia de una importante variabilidad inter e intrarregional en la atención sanitaria nefrológica mundial, con carencias significativas en cuanto a personal sanitario especializado, prestación de servicios sanitarios, medicamentos y tecnologías esenciales, financiación, liderazgo y gobernanza, sistemas de información sanitaria, estrategias, políticas; y capacidad de investigación y desarrollo, sobre todo en los países con ingresos económicos medios y bajos. Al identificar las lagunas en los dominios de la asistencia sanitaria universal, estos resultados han proporcionado una plataforma para aumentar la sensibilización sobre la enfermedad renal crónica (ERC) y una base para una red global de vigilancia y evaluación comparativa de la ERC.

La tercera versión de la encuesta realizada por la ISN tiene por objetivo comprender, comparar y supervisar el modo en que diferentes países del mundo detectan, tratan, supervisan y defienden a las personas con enfermedad renal crónica, haciendo especial hincapié en la capacidad, la disponibilidad, la accesibilidad, la asequibilidad y los resultados.

Con ella se intentará determinar los recursos y la disposición de los países para lograr el acceso universal a una atención sanitaria integral y equitativa en nefrología (que incluyen las terapias de reemplazo renal [TRR] y el tratamiento conservador). Esta versión también incluye una encuesta a pacientes que ofrece una perspectiva de los pacientes sobre el acceso a la atención y la calidad de la misma.

La presente encuesta aborda los puntos principales que conforman los distintos aspectos de la cobertura universal específicos para una atención sanitaria integral de la enfermedad renal crónica: la financiación, el personal sanitario, el acceso a medicamentos y productos sanitarios esenciales, los sistemas de información sanitaria y estadísticas, las políticas, la prestación de servicios y la seguridad, así como la respuesta de la comunidad nefrológica y los recursos para investigación y desarrollo en el tratamiento de las enfermedades renales.

Tras consultar a las 10 Juntas Regionales del ISN, la encuesta original en inglés se traduce al francés y al español, siendo estas lenguas representativas de las habladas y entendidas por la mayoría de los encuestados.

Si tiene alguna pregunta sobre cómo cumplimentar la encuesta, póngase en contacto con: Sandrine Damster (correo electrónico: GlobalAtlas@theisn.org).

Le damos las gracias por su implicación y su disposición a participar.

Profesora Agnes Fogo

Presidenta de la Sociedad Internacional de Nefrología (ISN)

Lista de abreviaturas:

DP: Diálisis peritoneal
DPA: Diálisis peritoneal automatizada
ENT: Enfermedad no transmisible
ERC: Enfermedad renal crónica
ERC estadio 5: Enfermedad Renal Crónica estadio 5
Fístula AV: Fístula arteriovenosa
HD: Hemodiálisis
LRA: Lesión renal aguda
ONG: Organización no gubernamental
PA: Presión arterial
PTH: Hormona paratiroidea
TRC: Tratamiento renal conservador
TRR: Terapia de reemplazo renal (es decir, hemodiálisis, diálisis peritoneal, trasplante renal)

1. ID de la encuesta (incluida en el correo electrónico): ________________________________

2. Cargo actual:

____

3. Función: Marque todas las respuestas adecuadas

◯ Nefrólogo/a
◯ Nefrólogo/a pediátrico
◯ Médico no nefrólogo
◯ Profesional sanitario (no médico)
◯ Gerente, autoridad responsable o funcionario
◯ Otra (especifique)

3. Si ha seleccionado "otros" por favor especifique.

____

4. ¿Cuál es su país de residencia?

- ◯ Afghanistan
- ◯ Åland Islands
- ◯ Albania
- ◯ Algeria
- ◯ American Samoa
- ◯ Andorra
- ◯ Angola
- ◯ Anguilla
- ◯ Antarctica
- ◯ Antigua And Barbuda
- ◯ Argentina
- ◯ Armenia
- ◯ Aruba
- ◯ Australia
- ◯ Austria
- ◯ Azerbaijan
- ◯ Bahamas
- ◯ Bahrain
- ◯ Bangladesh
- ◯ Barbados
- ◯ Belarus
- ◯ Belgium
- ◯ Belize
- ◯ Benin
- ◯ Bermuda
- ◯ Bhutan
- ◯ Bolivia
- ◯ Bosnia And Herzegovina
- ◯ Botswana
- ◯ Bouvet Island
- ◯ Brazil
- ◯ British Indian Ocean Territory
- ◯ Brunei Darussalam
- ◯ Bulgaria
- ◯ Burkina Faso
- ◯ Burundi
- ◯ Cambodia
- ◯ Cameroon
- ◯ Canada
- ◯ Cape Verde
- ◯ Cayman Islands
- ◯ Central African Republic
- ◯ Chad
- ◯ Chile
- ◯ China
- ◯ Christmas Island
- ◯ Cocos (Keeling) Islands
- ◯ Colombia
- ◯ Comoros
- ◯ Congo
- ◯ Congo (The Democratic Republic Of The)
- ◯ Cook Islands
- ◯ Costa Rica
- ◯ Cote D'ivoire
- ◯ Croatia
- ◯ Cuba
- ◯ Cyprus
- ◯ Czechia
- ◯ Denmark
- ◯ Djibouti
- ◯ Dominica
- ◯ Dominican Republic
- ◯ Ecuador
- ◯ Egypt
- ◯ El Salvador
- ◯ Equatorial Guinea
- ◯ Eritrea
- ◯ Estonia
- ◯ Ethiopia

◯ Falkland Islands (Malvinas)
◯ Faroe Islands
◯ Fiji
◯ Finland
◯ France
◯ French Guiana
◯ French Polynesia
◯ French Southern Territories
◯ Gabon
◯ Gambia
◯ Georgia
◯ Germany
◯ Ghana
◯ Gibraltar
◯ Greece
◯ Greenland
◯ Grenada
◯ Guadeloupe
◯ Guam
◯ Guatemala
◯ Guernsey
◯ Guinea
◯ Guinea-bissau
◯ Guyana
◯ Haiti
◯ Heard Island And Mcdonald Islands
◯ Holy See (Vatican City State)
◯ Honduras
◯ Hong Kong
◯ Hungary
◯ Iceland
◯ India
◯ Indonesia
◯ Iran, Islamic Republic Of
◯ Iraq
◯ Ireland
◯ Isle Of Man
◯ Israel
◯ Italy
◯ Jamaica
◯ Japan
◯ Jersey
◯ Jordan
◯ Kazakhstan
◯ Kenya
◯ Kiribati
◯ Korea: Democratic People's Republic Of
◯ Korea: Republic Of
◯ Kosovo
◯ Kuwait
◯ Kyrgyzstan
◯ Lao People's Democratic Republic
◯ Latvia
◯ Lebanon
◯ Lesotho
◯ Liberia
◯ Libyan Arab Jamahiriya
◯ Liechtenstein
◯ Lithuania
◯ Luxembourg
◯ Macao
◯ Macedonia
◯ Madagascar
◯ Malawi
◯ Malaysia
◯ Maldives
◯ Mali
◯ Malta
◯ Marshall Islands
◯ Martinique
◯ Mauritania

◯ Mauritius
◯ Mayotte
◯ Mexico
◯ Micronesia
◯ Moldova, Republic Of
◯ Monaco
◯ Mongolia
◯ Montenegro
◯ Montserrat
◯ Morocco
◯ Mozambique
◯ Myanmar
◯ Namibia
◯ Nauru
◯ Nepal
◯ Netherlands
◯ Netherlands Antilles
◯ New Caledonia
◯ New Zealand
◯ Nicaragua
◯ Niger
◯ Nigeria
◯ Niue
◯ Norfolk Island
◯ Northern Mariana Islands
◯ Norway
◯ Oman
◯ Pakistan
◯ Palau
◯ Palestinian Territory, Occupied
◯ Panama
◯ Papua New Guinea
◯ Paraguay
◯ Peru
◯ Philippines
◯ Pitcairn
◯ Poland
◯ Portugal
◯ Puerto Rico
◯ Qatar
◯ Reunion
◯ Romania
◯ Russian Federation
◯ Rwanda
◯ Saint Helena
◯ Saint Kitts And Nevis
◯ Saint Lucia
◯ Saint Pierre And Miquelon
◯ Saint Vincent And The Grenadines
◯ Samoa
◯ San Marino
◯ Sao Tome And Principe
◯ Saudi Arabia
◯ Senegal
◯ Serbia
◯ Seychelles
◯ Sierra Leone
◯ Singapore
◯ Slovakia
◯ Slovenia
◯ Solomon Islands
◯ Somalia
◯ South Africa
◯ South Georgia And The South Sandwich Islands
◯ South Sudan
◯ Spain
◯ Sri Lanka
◯ Sudan
◯ Suriname
◯ Svalbard And Jan Mayen
◯ Swaziland

◯ Sweden
◯ Switzerland
◯ Syrian Arab Republic
◯ Taiwan
◯ Tajikistan
◯ Tanzania, United Republic Of
◯ Thailand
◯ Timor-leste
◯ Togo
◯ Tokelau
◯ Tonga
◯ Trinidad And Tobago
◯ Tunisia
◯ Turkey
◯ Turkmenistan
◯ Turks And Caicos Islands
◯ Tuvalu
◯ Uganda
◯ Ukraine
◯ United Arab Emirates
◯ United Kingdom
◯ United States
◯ United States Minor Outlying Islands
◯ Uruguay
◯ Uzbekistan
◯ Vanuatu
◯ Venezuela
◯ Viet Nam
◯ Virgin Islands, British
◯ Virgin Islands, U.S.
◯ Wallis And Futuna
◯ Western Sahara
◯ Yemen
◯ Zambia
◯ Zimbabwe

---

5. ¿Cuál es su localidad de residencia?

____

A. Si se trata de una mezcla entre financiación pública y privada, explique.

A1. Sistema sanitario y mecanismos de financiación

A1.1. ¿Cuál es la mejor descripción, en general, de la estructura de financiación para la ERC sin diálisis en su sistema sanitario? (Elija la respuesta más adecuada).

- ◯ Financiada por el estado y gratuita para el usuario
- ◯ Financiada por el estado, pero con copago para el usuario
- ◯ Un sistema combinado de financiación pública (sea o no gratuita para el usuario) y privada (explíquelo)
- ◯ Financiación exclusivamente privada y de pago directo
- ◯ Financiación exclusivamente privada a través de compañías de seguros
- ◯ Varios sistemas: programas estatales, de Organizaciones No Gubernamentales (ONGs) y de colectivos
- ◯ Otra (especifique)

A1.1. Si ha seleccionado "otros" por favor especifique.

____

A1.1. Si se trata de una mezcla entre financiación pública y privada, explique.

____

A1.2. ¿Cuál es la mejor descripción, en general, de la estructura de financiación de su sistema sanitario para el TRR (tratamiento de reemplazo renal)? (Elija la respuesta más adecuada).

Reduzca el tamaño de la letra si el texto de abajo se superpone.

	Financiados por el estado y gratuitos para el usuario	Financiados por el estado, pero con copago para el usuario	Una combinación de sistemas de financiación pública (sea o no gratuita para el usuario) y privada (especifique)	Financiación exclusivamente privada y de pago directo	Financiación exclusivamente privada a través de compañías de seguros	Varios sistemas: programas estatales, de ONGs y de colectivos	Otro (especifique)	N/A (en mi país no existe esta modalidad)
A1.2.1. Diálisis aguda para ILRA (hemodiálisis o diálisis peritoneal)	○	○	○	○	○	○	○	○

A1.2. ¿Cuál es la mejor descripción, en general, de la estructura de financiación de su sistema sanitario para el TRR (tratamiento de reemplazo renal)? (Elija la respuesta más adecuada).

Reduzca el tamaño de la letra si el texto de abajo se superpone.

A1.2.2. Hemodiálisis crónica	○	○	○	○	○	○	○	○

A1.2. ¿Cuál es la mejor descripción, en general, de la estructura de financiación de su sistema sanitario para el TRR (tratamiento de reemplazo renal)? (Elija la respuesta más adecuada).

Reduzca el tamaño de la letra si el texto de abajo se superpone.

A1.2.3. Diálisis peritoneal crónica	○	○	○	○	○	○	○	○

A1.2. ¿Cuál es la mejor descripción, en general, de la estructura de financiación de su sistema sanitario para el TRR (tratamiento de reemplazo renal)? (Elija la respuesta más adecuada).

Reduzca el tamaño de la letra si el texto de abajo se superpone.

A1.2.4. Medicamentos para el trasplante renal ○ ○ ○ ○ ○ ○ ○ ○

A1.2.1. Si ha seleccionado "otros" por favor especifique. ______________________

A1.2.1. Si se trata de una mezcla entre financiación pública y privada, explique. ______________________

A1.2.2. Si ha seleccionado "otros" por favor especifique. ______________________

A1.2.2. Si se trata de una mezcla entre financiación pública y privada, explique. ______________________

A1.2.3. Si ha seleccionado "otros" por favor especifique. ______________________

A1.2.3. Si se trata de una mezcla entre financiación pública y privada, explique. ______________________

A1.2.4. Si ha seleccionado "otros" por favor especifique. ______________________

A1.2.4. Si se trata de una mezcla entre financiación pública y privada, explique. ______________________

A1.3.1. Si el TRRes de financiación pública (en parte o en su totalidad), ¿la cobertura es universal? (Es decir, ¿tienen derecho a atención sanitaria todas las personas que residen en su país?)

- ○ Sí, todos los residentes (incluidos los niños) están incluidos en la cobertura
- ○ No, no todos los residentes están incluidos (indique los detalles)

A1.3.1. Si ha seleccionado "no", por favor especifique, ______________________

A1.3.2. Si el TRR es de financiación pública (en su totalidad o en parte), ¿son elegibles para participar las poblaciones vulnerables (refugiados, desplazados)?

- ○ Sí, todas las poblaciones vulnerables están incluidas en la cobertura
- ○ No, no todas las poblaciones vulnerables están incluidas (indique los detalles)

A1.3.2. Si ha seleccionado "no", por favor especifique. ______________________

A1.3.3. Si el TRR es de financiación pública (en parte o en su totalidad), ¿qué aspectos de la atención sanitaria no están incluidos en la cobertura? Marque todas las respuestas adecuadas.	☐ Diálisis<br>☐ Trasplante<br>☐ Tratamiento conservador integral (servicios de tratamiento paliativo de apoyo renal)<br>☐ Tratamiento de las complicaciones (anemia, osteodistrofia renal, malnutrición)<br>☐ Ninguno (se financian todos los aspectos)<br>☐ Otros (especifique)
A1.3.3. Si ha seleccionado "otros" por favor especifique.	__________________________________

A1.4. ¿Cuál es la mejor descripción de la cobertura de su sistema sanitario para los servicios quirúrgicos en el TRR? (elija la respuesta más adecuada para cada fila) (saltar esta sección si el TRR no está disponible en su país)

Reduzca el tamaño de la letra si el texto de abajo se superpone.

	Financiados por el estado y gratuitos para el usuario	Financiados por el estado, pero con copago para el usuario	Una combinación de sistemas de financiación pública (sea o no gratuita para el usuario) y privada (especifique)	Financiación exclusivamente privada y de pago directo	Financiación exclusivamente privada a través de compañías de seguros	Varios sistemas: programas estatales, de ONGs y de colectivos	Otro (especifique)
A1.4.1. Acceso vascular para la hemodiálisis (catéteres venosos centrales)	○	○	○	○	○	○	○

A1.4. ¿Cuál es la mejor descripción de la cobertura de su sistema sanitario para los servicios quirúrgicos en el TRR? (elija la respuesta más adecuada para cada fila) (saltar esta sección si el TRR no está disponible en su país)

Reduzca el tamaño de la letra si el texto de abajo se superpone.

A1.4.2. Acceso vascular para la hemodiálisis (fístula o prótesis)	○	○	○	○	○	○	○

A1.4. ¿Cuál es la mejor descripción de la cobertura de su sistema sanitario para los servicios quirúrgicos en el TRR? (elija la respuesta más adecuada para cada fila) (saltar esta sección si el TRR no está disponible en su país)

Reduzca el tamaño de la letra si el texto de abajo se superpone.

A1.4.3. Cirugía de acceso para la diálisis peritoneal (inserción de catéter de DP)	○	○	○	○	○	○	○

A1.4. ¿Cuál es la mejor descripción de la cobertura de su sistema sanitario para los servicios quirúrgicos en el TRR? (elija la respuesta más adecuada para cada fila) (saltar esta sección si el TRR no está disponible en su país)

Reduzca el tamaño de la letra si el texto de abajo se superpone.

A1.4.4. Cirugía para trasplante renal ○ ○ ○ ○ ○ ○ ○

A1.4.1. Si ha seleccionado "otro", por favor especifique. ____________________

A1.4.1. Si se trata de una mezcla entre financiación pública y privada, explique. ____________________

A1.4.2. Si ha seleccionado "otro", por favor especifique. ____________________

A1.4.2. Si se trata de una mezcla entre financiación pública y privada, explique. ____________________

A1.4.3. Si ha seleccionado "otro", por favor especifique. ____________________

A1.4.3. Si se trata de una mezcla entre financiación pública y privada, explique. ____________________

A1.4.4. Si ha seleccionado "otro", por favor especifique. ____________________

A1.4.4. Si se trata de una mezcla entre financiación pública y privada, explique. ____________________

A2. Variabilidad dentro de un mismo país

Nos interesa conocer la variación dentro del país en la prestación de asistencia en caso de Enfermedad Renal Crónica etapa 5 (Enfermedad Renal Crónica Terminal), así como la variación entre países.

A2.1.1. ¿Difiere la organización o la prestación de asistencia en caso de ERC estadio 5 a escala regional en su país?

- ◯ Sí (si es posible, indique brevemente los detalles)
- ◯ No
- ◯ No lo sé

A2.1.1. Si ha seleccionado "si" por favor especifique.

____

A2.1.2. ¿Difiere el coste de la asistencia en caso de ERC estadio 5 a escala regional en su país?

- ◯ Sí (si es posible, indique brevemente los detalles)
- ◯ No
- ◯ No lo sé

A2.1.2. Si ha seleccionado "si", por favor especifique.

____

A2.1.3. ¿En su país existen diferencias entre los niños y los adultos con respecto a la organización o la prestación de la atención sanitaria en ERC estadio 5?

- ◯ Sí (si es posible, indique brevemente los detalles)
- ◯ No
- ◯ No lo sé

A2.1.3. Si ha seleccionado "si", por favor especifique.

____

A2.1.4. ¿En su país existen diferencias entre los niños y los adultos en cuanto al acceso al TRR?

- ◯ Sí (si es posible, indique brevemente los detalles)
- ◯ No
- ◯ No lo sé

A2.1.4. Si ha seleccionado "si", por favor especifique.

____

A2.2.1 Si los servicios de TRR no son iguales en los niños y en los adultos, ¿cuál es la diferencia en cuanto al acceso a la hemodiálisis?

- ◯ Más fácil acceso a HD para los adultos que para los niños
- ◯ Más fácil acceso a HD para los niños que para los adultos
- ◯ Acceso a HD disponible para adultos, pero no disponible para niños
- ◯ Acceso a HD disponible para niños, pero no disponible para adultos

A2.2.2 Si los servicios de TRR no son iguales en los niños y en los adultos, ¿cuál es la diferencia en cuanto al acceso a la diálisis peritoneal?	◯ Más fácil acceso a DP para los adultos que para los niños<br>◯ Más fácil acceso a DP para los niños que para los adultos<br>◯ Acceso a DP disponible para adultos, pero no disponible para niños<br>◯ Acceso a DP disponible para niños, pero no disponible para adultos
A2.2.3 Si los servicios de TRR no son iguales en los niños y en los adultos, ¿cuál es la diferencia en cuanto al acceso al trasplante de riñón?	◯ Más fácil acceso a trasplante para los adultos que para los niños<br>◯ Más fácil acceso a trasplante para los niños que para los adultos<br>◯ Acceso a trasplante disponible para adultos, pero no disponible para niños<br>◯ Acceso a trasplante disponible para niños, pero no disponible para adultos

A3. Supervisión

A3.1. ¿Cuál es la mejor descripción de la gestión o supervisión de la atención sanitaria de la enfermedad renal en su país? Marque todas las respuestas adecuadas.	☐ Gestionada o supervisada por un organismo nacional<br>☐ Gestionada o supervisada únicamente por las autoridades provinciales, regionales o autonómicas<br>☐ Gestionada por centros hospitalarios, fundaciones u organizaciones individuales<br>☐ Gestionada por ONGs<br>☐ Otra (especifique)<br>☐ No existe ningún sistema organizado
A3.1. Si ha seleccionado "otra", por favor especifique.	__________________________________
A3.2. ¿Cómo calificaría la infraestructura sanitaria de su país en términos de adecuación para la atención de la ERC estadio 5?	◯ Muy deficiente<br>◯ Deficiente/inferior a la media<br>◯ Regular/en la media<br>◯ Buena/superior a la media<br>◯ Excelente

B. Personal sanitario para la atención nefrológica
B1. Responsabilidad clínica

B1.1. ¿Quién es el principal responsable clínico de la atención sanitaria de la ERC estadio 5 en su país?

- ◯ Nefrólogos
- ◯ Médicos de atención primaria
- ◯ Profesionales de enfermería
- ◯ Enfermeros especializados
- ◯ Equipos multidisciplinares
- ◯ Auxiliares médicos o agentes de divulgación sanitaria
- ◯ Otros especialistas (especifique)

B1.1. Si ha seleccionado "otros", por favor especifique.

____

B2.1.1. ¿Cuántos nefrólogos hay en su país aproximadamente? Déjelo en blanco si no lo sabe.
Nefrólogos {b2_1_1_neph_esp}
Nefrólogos adultos {b2_1_1_ad_neph_esp}
Nefrólogos pediátricos {b2_1_1_paed_neph_esp}

B2.1.2. ¿Cuál es el porcentaje de nefrólogos mujeres(de adultos y pediátricos combinados) en su país? Déjelo en blanco si no lo sabe.

____

B2.2.1. ¿Cuántos nefrólogos en formación hay aproximadamente en su país? Déjelo en blanco si no lo sabe.

____

B2.2.2. ¿Existe en su país un programa de formación para nefrólogos adultos?

- ◯ Si
- ◯ No
- ◯ No estoy seguro

B2.2.3. Si respondió afirmativamente a la pregunta B2.2.2 anterior, ¿cuál es la duración del programa de formación?

- ◯ < 1 año
- ◯ 1 - 2 años
- ◯ 2 - 4 años
- ◯ > 4 años

B2.2.4. ¿Existe en su país un programa de formación para nefrólogos pediatras?

- ◯ Si
- ◯ No
- ◯ No estoy seguro

B2.2.5. Si respondió afirmativamente a la pregunta B2.2.4, ¿cuál es la duración del programa de formación?

- ◯ < 1 año
- ◯ 1 - 2 años
- ◯ 2 - 4 años
- ◯ > 4 años

B2.2.6. ¿Está vinculado el programa de formación para nefrólogos (adultos o pediátrias) a un componente de investigación (p. ej., doctorado, máster en ciencias, máster en medicina, etc.)?

- ◯ Si
- ◯ No
- ◯ No estoy seguro

B2.3. En su opinión, ¿existe en su país escasez de alguno de los siguientes profesionales para la atención sanitaria de la enfermedad renal? Marque todas las respuestas adecuadas.

- ☐ Nefrólogos
- ☐ Nefrólogos pediátricos
- ☐ Cirujanos especialistas en trasplantes
- ☐ Cirujanos o radiólogos intervencionistas (capaces de colocar un acceso arteriovenoso para la hemodiálisis)
- ☐ Cirujanos o radiólogos intervencionistas (capaces de colocar un acceso para diálisis peritoneal)
- ☐ Dietistas-nutricionistas
- ☐ Técnicos de laboratorio
- ☐ Radiólogos que realicen e interpreten ecografías renales
- ☐ Coordinadores para accesos vasculares
- ☐ Orientadores o psicólogos
- ☐ Coordinadores de trasplantes
- ☐ Enfermeras de diálisis
- ☐ Enfermeros renales
- ☐ Técnicos de diálisis
- ☐ Trabajadores sociales
- ☐ Médicos de cuidados paliativos
- ☐ Profesionales de enfermería que prestan asistencia renal
- ☐ No existe escasez de ninguno de los profesionales mencionados

C. Acceso a medicamentos y productos sanitarios esenciales para la atención sanitaria de las enfermedades renales

C1. Recursos para la prestación de servicios de TRR

C1.1. ¿Existe en su país la hemodiálisis en el centro (pediátrica y para adultos)?	◯ Si<br>◯ No
C1.1.1. Si la respuesta es afirmativa, ¿en cuántos centros de su país se hace hemodiálisis crónica?	__________________________
C1.1.2 ¿Se dispone de hemodiálisis domiciliaria (adulta y pediátrica) en su país?	◯ Si<br>◯ No
C1.2. ¿Existe en su país la diálisis peritoneal (pediátrica y para adultos)?	◯ Si<br>◯ No
C1.2.1 Si la respuesta es afirmativa, ¿cuántos centros de su país hacen diálisis peritoneal crónica?	__________________________
C1.2.2. Si se dispone de diálisis peritoneal, ¿en qué circunstancias se puede acceder a diálisis peritoneal en su país? (Seleccione una opción)	◯ Sólo diálisis peritoneal aguda<br>◯ Diálisis peritoneal aguda y crónica<br>◯ Sólo diálisis peritoneal crónica
C1.2.3. ¿Existe en su país la diálisis peritoneal automatizada (DPA) (pediátrica y para adultos)?	◯ Si<br>◯ No
C1.3. ¿Se hacen en su país trasplantes de riñón en adultos?	◯ Si<br>◯ No
C1.4. ¿Se hacen en su país trasplantes de riñón pediátricos (edad < 18 años)?	◯ Si<br>◯ No
C1.5.1. Si la respuesta es afirmativa, ¿cuál es el origen de los riñones donados? (Elija la respuesta más adecuada).	◯ Donante cadáver únicamente<br>◯ Donante vivo únicamente<br>◯ Tanto donante vivo como donante cadáver
C1.5.2. Si los riñones para trasplante proceden tanto de donante vivo como de donante cadáver, ¿cuál es el porcentaje de donantes vivos?	__________________________
C1.5.3. Si en su país existe el trasplante de riñón, ¿cómo son las listas de espera para el trasplante?	◯ Hay un registro nacional<br>◯ Hay listas regionales<br>◯ No hay listas de espera
C1.5.4. Si en su país existe el trasplante de riñón, ¿en cuántos centros se hacen trasplantes de riñón?	__________________________

C2. Acceso a medicamentos y productos sanitarios esenciales

C2.1. Medicamentos y tecnologías esenciales para el TRR: acceso, asequibilidad y planes de reembolso y calidad
(elija la respuesta más adecuada para cada pregunta)

Reduzca el tamaño de la letra si el texto de abajo se superpone.

	Financiados por el estado y gratuitos para el usuario	Financiados por el estado, pero con copago para el usuario	Una combinación de sistemas de financiación pública (sea o no gratuita para el usuario) y privada (especifique)	Financiación exclusivamente privada y de pago directo	Financiación exclusivamente privada a través de compañías de seguros	Varios sistemas: programas estatales, de ONGs y de colectivos	Otro (especifique)
C2.1.1. Para todos los pacientes con ERC (no sometidos a diálisis): ¿Cómo se financian los medicamentos?	○	○	○	○	○	○	○

C2. Acceso a medicamentos y productos sanitarios esenciales

C2.1. Medicamentos y tecnologías esenciales para el TRR: acceso, asequibilidad y planes de reembolso y calidad
(elija la respuesta más adecuada para cada pregunta)

Reduzca el tamaño de la letra si el texto de abajo se superpone.

C2.1.2. Para todos los pacientes en diálisis: ¿Cómo se financian los medicamentos?	○	○	○	○	○	○	○

C2. Acceso a medicamentos y productos sanitarios esenciales

C2.1. Medicamentos y tecnologías esenciales para el TRR: acceso, asequibilidad y planes de reembolso y calidad
(elija la respuesta más adecuada para cada pregunta)

Reduzca el tamaño de la letra si el texto de abajo se superpone.

C2.1.3. Para todos los pacientes trasplantados: ¿Cómo se financian los medicamentos? ○ ○ ○ ○ ○ ○ ○

C2.1.1. Si ha seleccionado "otro", por favor especifique. ______________________

C2.1.1. Si se trata de una mezcla entre financiación pública y privada, explique. ______________________

C2.1.2. Si ha seleccionado "otro", por favor especifique. ______________________

C2.1.2. Si se trata de una mezcla entre financiación pública y privada, explique. ______________________

C2.1.3. Si ha seleccionado "otro", por favor especifique. ______________________

C2.1.3. Si se trata de una mezcla entre financiación pública y privada, explique. ______________________

C3. Preparación para TRR
Atención óptima de la ERC estadio 5: En el marco de la visión, misión y valores de la ISN, creemos que todos los pacientes que estén alcanzando la ERC estadio 5 deben ser preparados a tiempo para el TRR a fin de reducir las complicaciones y la progresión de la enfermedad y de optimizar su elección de opciones terapéuticas adecuadas a su situación clínica. Las respuestas a las preguntas que aparecen a continuación son importantes para mejorar nuestros conocimientos sobre la disposición actual de servicios.

C3.1. Indique la disponibilidad de los siguientes servicios (pruebas y tratamientos) para la atención sanitaria de la ERC estadio 5 en su país.

Tenga en cuenta que significa en el 50 % o más de los centros sanitarios (hospitales o consultorios) y significa en menos del 50 % de los centros (hospitales o consultorios).

C3.1.1 Control de la concentración de hemoglobina

	En general disponible	En general no disponible	Nunca	No lo sé
C3.1.1. Determinación de la hemoglobina sérica	○	○	○	○

C3. Preparación para TRR
Atención óptima de la ERC estadio 5: En el marco de la visión, misión y valores de la ISN, creemos que todos los pacientes que estén alcanzando la ERC estadio 5 deben ser preparados a tiempo para el TRR a fin de reducir las complicaciones y la progresión de la enfermedad y de optimizar su elección de opciones terapéuticas adecuadas a su situación clínica. Las respuestas a las preguntas que aparecen a continuación son importantes para mejorar nuestros conocimientos sobre la disposición actual de servicios.

C3.1. Indique la disponibilidad de los siguientes servicios (pruebas y tratamientos) para la atención sanitaria de la ERC estadio 5 en su país.

Tenga en cuenta que significa en el 50 % o más de los centros sanitarios (hospitales o consultorios) y significa en menos del 50 % de los centros (hospitales o consultorios).

C3.1.1 Control de la concentración de hemoglobina

C3.1.2. Determinación de los valores férricos (hierro, ferritina, saturación de la transferrina)	○	○	○	○

C3. Preparación para TRR
Atención óptima de la ERC estadio 5: En el marco de la visión, misión y valores de la ISN, creemos que todos los pacientes que estén alcanzando la ERC estadio 5 deben ser preparados a tiempo para el TRR a fin de reducir las complicaciones y la progresión de la enfermedad y de optimizar su elección de opciones terapéuticas adecuadas a su situación clínica. Las respuestas a las preguntas que aparecen a continuación son importantes para mejorar nuestros conocimientos sobre la disposición actual de servicios.

C3.1. Indique la disponibilidad de los siguientes servicios (pruebas y tratamientos) para la atención sanitaria de la ERC estadio 5 en su país.

Tenga en cuenta que significa en el 50 % o más de los centros sanitarios (hospitales o consultorios) y significa en menos del 50 % de los centros (hospitales o consultorios).

C3.1.1 Control de la concentración de hemoglobina

C3.1.3. Determinación de los marcadores inflamatorios (por ejemplo, la proteína C reactiva en suero)	○	○	○	○

C3. Preparación para TRR
Atención óptima de la ERC estadio 5: En el marco de la visión, misión y valores de la ISN, creemos que todos los pacientes que estén alcanzando la ERC estadio 5 deben ser preparados a tiempo para el TRR a fin de reducir las complicaciones y la progresión de la enfermedad y de optimizar su elección de opciones terapéuticas adecuadas a su situación clínica. Las respuestas a las preguntas que aparecen a continuación son importantes para mejorar nuestros conocimientos sobre la disposición actual de servicios.

C3.1. Indique la disponibilidad de los siguientes servicios (pruebas y tratamientos) para la atención sanitaria de la ERC estadio 5 en su país.

Tenga en cuenta que significa en el 50 % o más de los centros sanitarios (hospitales o consultorios) y significa en menos del 50 % de los centros (hospitales o consultorios).

C3.1.1 Control de la concentración de hemoglobina

C3.1.4. Hierro por vía oral	○	○	○	○

C3. Preparación para TRR
Atención óptima de la ERC estadio 5: En el marco de la visión, misión y valores de la ISN, creemos que todos los pacientes que estén alcanzando la ERC estadio 5 deben ser preparados a tiempo para el TRR a fin de reducir las complicaciones y la progresión de la enfermedad y de optimizar su elección de opciones terapéuticas adecuadas a su situación clínica. Las respuestas a las preguntas que aparecen a continuación son importantes para mejorar nuestros conocimientos sobre la disposición actual de servicios.

C3.1. Indique la disponibilidad de los siguientes servicios (pruebas y tratamientos) para la atención sanitaria de la ERC estadio 5 en su país.

Tenga en cuenta que significa en el 50 % o más de los centros sanitarios (hospitales o consultorios) y significa en menos del 50 % de los centros (hospitales o consultorios).

C3.1.1 Control de la concentración de hemoglobina

C3.1.5. Hierro por vía parenteral	○	○	○	○

C3. Preparación para TRR
Atención óptima de la ERC estadio 5: En el marco de la visión, misión y valores de la ISN, creemos que todos los pacientes que estén alcanzando la ERC estadio 5 deben ser preparados a tiempo para el TRR a fin de reducir las complicaciones y la progresión de la enfermedad y de optimizar su elección de opciones terapéuticas adecuadas a su situación clínica. Las respuestas a las preguntas que aparecen a continuación son importantes para mejorar nuestros conocimientos sobre la disposición actual de servicios.

C3.1. Indique la disponibilidad de los siguientes servicios (pruebas y tratamientos) para la atención sanitaria de la ERC estadio 5 en su país.

Tenga en cuenta que significa en el 50 % o más de los centros sanitarios (hospitales o consultorios) y significa en menos del 50 % de los centros (hospitales o consultorios).

C3.1.1 Control de la concentración de hemoglobina

C3.1.6. Agente estimulante de la eritropoyesis (p. ej., eritropoyetina)	○	○	○	○

C3.1.2. Tratamiento de la osteopatía mineral

Tenga en cuenta que significa en el 50 % o más de los centros sanitarios (hospitales o consultorios) y significa en menos del 50 % de los centros (hospitales o consultorios).

	En general disponible	En general no disponible	Nunca	No lo sé
C3.1.2.1. Determinación del calcio sérico	○	○	○	○

C3.1.2. Tratamiento de la osteopatía mineral

Tenga en cuenta que significa en el 50 % o más de los centros sanitarios (hospitales o consultorios) y significa en menos del 50 % de los centros (hospitales o consultorios).

C3.1.2.2. Determinación del fósforo sérico	○	○	○	○

C3.1.2. Tratamiento de la osteopatía mineral

Tenga en cuenta que significa en el 50 % o más de los centros sanitarios (hospitales o consultorios) y significa en menos del 50 % de los centros (hospitales o consultorios).

C3.1.2.3. Determinación de la hormona paratiroidea (PTH) sérica	○	○	○	○

C3.1.2. Tratamiento de la osteopatía mineral

Tenga en cuenta que significa en el 50 % o más de los centros sanitarios (hospitales o consultorios) y significa en menos del 50 % de los centros (hospitales o consultorios).

C3.1.2.4. Quelantes del fosfato a base de calcio	○	○	○	○

C3.1.2. Tratamiento de la osteopatía mineral

Tenga en cuenta que significa en el 50 % o más de los centros sanitarios (hospitales o consultorios) y significa en menos del 50 % de los centros (hospitales o consultorios).

C3.1.2.5. Quelantes del fosfato sin calcio (por ejemplo, el sevelámero)	◯	◯	◯	◯

C3.1.2. Tratamiento de la osteopatía mineral

Tenga en cuenta que significa en el 50 % o más de los centros sanitarios (hospitales o consultorios) y significa en menos del 50 % de los centros (hospitales o consultorios).

C3.1.2.6. Cinacalcet	◯	◯	◯	◯

C3.1.2. Tratamiento de la osteopatía mineral

Tenga en cuenta que significa en el 50 % o más de los centros sanitarios (hospitales o consultorios) y significa en menos del 50 % de los centros (hospitales o consultorios).

C3.1.2.7. Servicios quirúrgicos para la paratiroidectomía	○	○	○	○

C3.1.3. Control de los trastornos electrolíticos y la acidosis metabólica crónica

Tenga en cuenta que significa en el 50 % o más de los centros sanitarios (hospitales o consultorios) y significa en menos del 50 % de los centros (hospitales o consultorios).

	En general disponible	En general no disponible	Nunca	No lo sé
C3.1.3.1. Determinación de los electrólitos séricos (sodio, potasio, cloruros, etc.)Determinación de los electrólitos séricos (sodio, potasio, cloruros, etc.)	○	○	○	○

C3.1.3. Control de los trastornos electrolíticos y la acidosis metabólica crónica

Tenga en cuenta que significa en el 50 % o más de los centros sanitarios (hospitales o consultorios) y significa en menos del 50 % de los centros (hospitales o consultorios).

C3.1.3.2. Determinación del bicarbonato sérico	○	○	○	○

C3.1.3. Control de los trastornos electrolíticos y la acidosis metabólica crónica

Tenga en cuenta que significa en el 50 % o más de los centros sanitarios (hospitales o consultorios) y significa en menos del 50 % de los centros (hospitales o consultorios).

C3.1.3.3. Resinas de intercambio del potasio (por ejemplo, el sulfonato de poliestireno sódico, zirconio sódico patirómero)	○	○	○	○

C3.1.3. Control de los trastornos electrolíticos y la acidosis metabólica crónica

Tenga en cuenta que significa en el 50 % o más de los centros sanitarios (hospitales o consultorios) y significa en menos del 50 % de los centros (hospitales o consultorios).

C3.1.3.4. Bicarbonato sódico por vía oral	○	○	○	○

C3.1.4. Control de la tensión arterial

Tenga en cuenta que significa en el 50 % o más de los centros sanitarios (hospitales o consultorios) y significa en menos del 50 % de los centros (hospitales o consultorios).

	En general disponible	En general no disponible	Nunca	No lo sé
C3.1.4.1. Medición analógica de la TA	◯	◯	◯	◯

C3.1.4. Control de la tensión arterial

Tenga en cuenta que significa en el 50 % o más de los centros sanitarios (hospitales o consultorios) y significa en menos del 50 % de los centros (hospitales o consultorios).

C3.1.4.2. Medición automatizada de la TA (en el domicilio o en la consulta)	○	○	○	○

C3.1.4. Control de la tensión arterial

Tenga en cuenta que significa en el 50 % o más de los centros sanitarios (hospitales o consultorios) y significa en menos del 50 % de los centros (hospitales o consultorios).

C3.1.4.3. Medición ambulatoria de la TA (MATA)	○	○	○	○

C3.1.5. Tratamiento de los síntomas frecuentes asociados a la insuficiencia renal (ERT) (prurito urémico, piernas inquietas, dolor)

Tenga en cuenta que significa en el 50 % o más de los centros sanitarios (hospitales o consultorios) y significa en menos del 50 % de los centros (hospitales o consultorios).

	En general disponible	En general no disponible	Nunca	No lo sé
C3.1.5.1. Gabapentinoides (gabapentina o pregabalina)	◯	◯	◯	◯

C3.1.5. Tratamiento de los síntomas frecuentes asociados a la insuficiencia renal (ERT) (prurito urémico, piernas inquietas, dolor)

Tenga en cuenta que significa en el 50 % o más de los centros sanitarios (hospitales o consultorios) y significa en menos del 50 % de los centros (hospitales o consultorios).

C3.1.5.2. Opioides distintos de la morfina (p. ej., hidromorfona, oxicodona, metadona y/o fentanilo sublingual o transdérmico)	○	○	○	○

C4.1. Servicios relacionados con la nutrición

C4.1.1. Indique la disponibilidad de los siguientes servicios nutricionales para la asistencia de la enfermedad renal en su país.

Tenga en cuenta que significa en el 50 % o más de los centros sanitarios (hospitales o consultorios) y significa en menos del 50 % de los centros (hospitales o consultorios).

	En general disponible	En general no disponible	Nunca	No lo sé
C4.1.1.1. Consejo dietético por parte de una persona con formación en nutrición (por ejemplo, un dietista-nutricionista)	◯	◯	◯	◯

C4.1. Servicios relacionados con la nutrición

C4.1.1. Indique la disponibilidad de los siguientes servicios nutricionales para la asistencia de la enfermedad renal en su país.

Tenga en cuenta que significa en el 50 % o más de los centros sanitarios (hospitales o consultorios) y significa en menos del 50 % de los centros (hospitales o consultorios).

C4.1.1.2. Determinación de la albúmina sérica	○	○	○	○

C4.1. Servicios relacionados con la nutrición

C4.1.1. Indique la disponibilidad de los siguientes servicios nutricionales para la asistencia de la enfermedad renal en su país.

Tenga en cuenta que significa en el 50 % o más de los centros sanitarios (hospitales o consultorios) y significa en menos del 50 % de los centros (hospitales o consultorios).

C4.1.1.3. Suplementos nutricionales por vía oral (por ejemplo, vitaminas, suplementos alimenticios)	○	○	○	○

C5. Diálisis: calidad y opciones

C5.1. Indique la disponibilidad de los siguientes servicios para la asistencia de diálisis en su país.

Opciones de modalidad

Tenga en cuenta que significa en el 50 % o más de los centros sanitarios (hospitales o consultorios) y significa en menos del 50 % de los centros (hospitales o consultorios).

	En general disponible	En general no disponible	Nunca	No lo sé	ND (no se realizan diálisis)
C5.1.1. Hemodiálisis en el centro	○	○	○	○	○

C5. Diálisis: calidad y opciones

C5.1. Indique la disponibilidad de los siguientes servicios para la asistencia de diálisis en su país.

Opciones de modalidad

Tenga en cuenta que significa en el 50 % o más de los centros sanitarios (hospitales o consultorios) y significa en menos del 50 % de los centros (hospitales o consultorios).

C5.1.2. Hemodiálisis domiciliaria	○	○	○	○	○

C5. Diálisis: calidad y opciones

C5.1. Indique la disponibilidad de los siguientes servicios para la asistencia de diálisis en su país.

Opciones de modalidad

Tenga en cuenta que significa en el 50 % o más de los centros sanitarios (hospitales o consultorios) y significa en menos del 50 % de los centros (hospitales o consultorios).

C5.1.3. Diálisis peritoneal	○	○	○	○	○

Calidad

Tenga en cuenta que significa en el 50 % o más de los centros sanitarios (hospitales o consultorios) y significa en menos del 50 % de los centros (hospitales o consultorios).

	En general disponible	En general no disponible	Nunca	No lo sé	ND (no se realizan diálisis)
C5.1.4. Hemodiálisis en centros sanitarios con la frecuencia idónea (tres veces por semana durante tres o cuatro horas)	○	○	○	○	○

Calidad<br><br>Tenga en cuenta que significa en el 50 % o más de los centros sanitarios (hospitales o consultorios) y significa en menos del 50 % de los centros (hospitales o consultorios).					
C5.1.5. Hemodiálisis domiciliaria (tratamiento tres veces por semana durante tres o cuatro horas)	○	○	○	○	○

Calidad

Tenga en cuenta que significa en el 50 % o más de los centros sanitarios (hospitales o consultorios) y significa en menos del 50 % de los centros (hospitales o consultorios).

C5.1.6. Diálisis peritoneal con intercambios de la frecuencia idónea (3 o 4 al día o ciclos equivalentes en la DP automatizada)	○	○	○	○	○

Calidad

Tenga en cuenta que significa en el 50 % o más de los centros sanitarios (hospitales o consultorios) y significa en menos del 50 % de los centros (hospitales o consultorios).

C5.1.7. Determinación de la eficacia de la diálisis peritoneal (es decir, midiendo el coeficiente de reducción de la urea [URR] y/o el Kt/V)	○	○	○	○	○

Calidad

Tenga en cuenta que significa en el 50 % o más de los centros sanitarios (hospitales o consultorios) y significa en menos del 50 % de los centros (hospitales o consultorios).

C5.1.8. Servicios de transporte asequibles para los pacientes	○	○	○	○	○

C6. Trasplante: calidad y opciones

Opción de trasplante: En el marco de la visión, misión y valores de la ISN, creemos que todos los pacientes con trasplante de riñón deben recibir un servicio de calidad que los apoye en el control de su trasplante y les permita lograr la mejor calidad de vida posible. Las respuestas a las preguntas que aparecen a continuación son importantes para mejorar nuestros conocimientos sobre la disposición actual de servicios.

C6.1 Indique la disponibilidad de los siguientes servicios para servicios de trasplante en su país.

Tenga en cuenta que significa en el 50 % o más de los centros sanitarios (hospitales o consultorios) y significa en menos del 50 % de los centros (hospitales o consultorios).

Si el trasplante NO existe en su país, elija ND.

	En general disponible	En general no disponible	Nunca	No lo sé	ND (no se hacen trasplantes)
C6.1.1. Información temprana y culturalmente adecuada a los pacientes, sus familiares y sus cuidadores sobre los riesgos y las ventajas del trasplante con una explicación clara de las pruebas, los procedimientos y los resultados	○	○	○	○	○

C6. Trasplante: calidad y opciones

Opción de trasplante: En el marco de la visión, misión y valores de la ISN, creemos que todos los pacientes con trasplante de riñón deben recibir un servicio de calidad que los apoye en el control de su trasplante y les permita lograr la mejor calidad de vida posible. Las respuestas a las preguntas que aparecen a continuación son importantes para mejorar nuestros conocimientos sobre la disposición actual de servicios.

C6.1 Indique la disponibilidad de los siguientes servicios para servicios de trasplante en su país.

Tenga en cuenta que significa en el 50 % o más de los centros sanitarios (hospitales o consultorios) y significa en menos del 50 % de los centros (hospitales o consultorios).

Si el trasplante NO existe en su país, elija ND.

C6.1.2. Profilaxis eficaz para controlar las infecciones (por ejemplo, antivíricos, antifúngicos, etc.)	○	○	○	○	○

C6. Trasplante: calidad y opciones

Opción de trasplante: En el marco de la visión, misión y valores de la ISN, creemos que todos los pacientes con trasplante de riñón deben recibir un servicio de calidad que los apoye en el control de su trasplante y les permita lograr la mejor calidad de vida posible. Las respuestas a las preguntas que aparecen a continuación son importantes para mejorar nuestros conocimientos sobre la disposición actual de servicios.

C6.1 Indique la disponibilidad de los siguientes servicios para servicios de trasplante en su país.

Tenga en cuenta que significa en el 50 % o más de los centros sanitarios (hospitales o consultorios) y significa en menos del 50 % de los centros (hospitales o consultorios).

Si el trasplante NO existe en su país, elija ND.

C6.1.3. Acceso a tiempo a un quirófano para el trasplante renal	○	○	○	○	○

C6. Trasplante: calidad y opciones

Opción de trasplante: En el marco de la visión, misión y valores de la ISN, creemos que todos los pacientes con trasplante de riñón deben recibir un servicio de calidad que los apoye en el control de su trasplante y les permita lograr la mejor calidad de vida posible. Las respuestas a las preguntas que aparecen a continuación son importantes para mejorar nuestros conocimientos sobre la disposición actual de servicios.

C6.1 Indique la disponibilidad de los siguientes servicios para servicios de trasplante en su país.

Tenga en cuenta que significa en el 50 % o más de los centros sanitarios (hospitales o consultorios) y significa en menos del 50 % de los centros (hospitales o consultorios).

Si el trasplante NO existe en su país, elija ND.

C6.1.4. Inmunosupresión adecuada y tratamiento antirrechazo	○	○	○	○	○

C6. Trasplante: calidad y opciones

Opción de trasplante: En el marco de la visión, misión y valores de la ISN, creemos que todos los pacientes con trasplante de riñón deben recibir un servicio de calidad que los apoye en el control de su trasplante y les permita lograr la mejor calidad de vida posible. Las respuestas a las preguntas que aparecen a continuación son importantes para mejorar nuestros conocimientos sobre la disposición actual de servicios.

C6.1 Indique la disponibilidad de los siguientes servicios para servicios de trasplante en su país.

Tenga en cuenta que significa en el 50 % o más de los centros sanitarios (hospitales o consultorios) y significa en menos del 50 % de los centros (hospitales o consultorios).

Si el trasplante NO existe en su país, elija ND.

C6.1.5. Instalaciones adecuadas para controlar la administración de inmunosupresores	○	○	○	○	○

C6. Trasplante: calidad y opciones

Opción de trasplante: En el marco de la visión, misión y valores de la ISN, creemos que todos los pacientes con trasplante de riñón deben recibir un servicio de calidad que los apoye en el control de su trasplante y les permita lograr la mejor calidad de vida posible. Las respuestas a las preguntas que aparecen a continuación son importantes para mejorar nuestros conocimientos sobre la disposición actual de servicios.

C6.1 Indique la disponibilidad de los siguientes servicios para servicios de trasplante en su país.

Tenga en cuenta que significa en el 50 % o más de los centros sanitarios (hospitales o consultorios) y significa en menos del 50 % de los centros (hospitales o consultorios).

Si el trasplante NO existe en su país, elija ND.

C6.1.6. Equipo multidisciplinario de apoyo a los pacientes con un trasplante de riñón	○	○	○	○	○

C6. Trasplante: calidad y opciones

Opción de trasplante: En el marco de la visión, misión y valores de la ISN, creemos que todos los pacientes con trasplante de riñón deben recibir un servicio de calidad que los apoye en el control de su trasplante y les permita lograr la mejor calidad de vida posible. Las respuestas a las preguntas que aparecen a continuación son importantes para mejorar nuestros conocimientos sobre la disposición actual de servicios.

C6.1 Indique la disponibilidad de los siguientes servicios para servicios de trasplante en su país.

Tenga en cuenta que significa en el 50 % o más de los centros sanitarios (hospitales o consultorios) y significa en menos del 50 % de los centros (hospitales o consultorios).

Si el trasplante NO existe en su país, elija ND.

C6.1.7. Marco contextual normalizado para la obtención de órganos (por ejemplo, legislación con respecto a la muerte cerebral)	○	○	○	○	○

C7. Tratamiento renal conservador (TRC)
Tratamiento renal conservador: El tratamiento renal conservador se define como la elección del paciente para una asistencia integral centrada en el paciente sin el uso de Terapia de Reemplazo Renal (TRR) en los pacientes con Enfermedad Renal Crónica (ERC) estadio 5. Los objetivos del tratamiento renal conservador son apoyar a los pacientes con ERC estadio 5 que no reciben TRR, optimizando la calidad de vida, controlando los síntomas, tratando la angustia psicosocial, facilitando la planificación anticipada de la atención y, en su caso, preservando la función renal residual. Esta asistencia incluye el apoyo a los familiares y cuidadores del paciente y continúa durante toda la trayectoria de la enfermedad. Esto es adecuado en pacientes que es improbable que se beneficien de las TRR o que opten por no iniciar TRR. Reconocemos que los pacientes pueden recibir una asistencia conservadora similar cuando las limitaciones de recursos (sistema sanitario o paciente) impiden o limitan el acceso a TRR. Denominamos a esto atención conservadora con restricción de elección.

Nos gustaría saber más sobre la capacidad de ofrecer un tratamiento renal conservador o una asistencia conservadora con restricción elección en su país (es decir, los recursos para el tratamiento paliativo o de control en los pacientes que no recibirán TRR a pesar de tener ERC estadio 5).

C7.1. Teniendo en cuenta la definición mencionada, ¿existe en su país el tratamiento conservador?

	En general disponible	En general no disponible	ND (TRC no disponible)	No lo sé
C7.1.1. Tratamiento renal conservador establecido que se elige mediante una toma de decisiones compartida (cuando el TRR está fácilmente disponible)	○	○	○	○

C7. Tratamiento renal conservador (TRC)
Tratamiento renal conservador: El tratamiento renal conservador se define como la elección del paciente para una asistencia integral centrada en el paciente sin el uso de Terapia de Reemplazo Renal (TRR) en los pacientes con Enfermedad Renal Crónica (ERC) estadio 5. Los objetivos del tratamiento renal conservador son apoyar a los pacientes con ERC estadio 5 que no reciben TRR, optimizando la calidad de vida, controlando los síntomas, tratando la angustia psicosocial, facilitando la planificación anticipada de la atención y, en su caso, preservando la función renal residual. Esta asistencia incluye el apoyo a los familiares y cuidadores del paciente y continúa durante toda la trayectoria de la enfermedad. Esto es adecuado en pacientes que es improbable que se beneficien de las TRR o que opten por no iniciar TRR. Reconocemos que los pacientes pueden recibir una asistencia conservadora similar cuando las limitaciones de recursos (sistema sanitario o paciente) impiden o limitan el acceso a TRR. Denominamos a esto atención conservadora con restricción de elección.

Nos gustaría saber más sobre la capacidad de ofrecer un tratamiento renal conservador o una asistencia conservadora con restricción elección en su país (es decir, los recursos para el tratamiento paliativo o de control en los pacientes que no recibirán TRR a pesar de tener ERC estadio 5).

C7.1. Teniendo en cuenta la definición mencionada, ¿existe en su país el tratamiento conservador?

C7.1.2. Atención conservadora establecida con restricción de elección (cuando existan limitaciones de recursos para impedir o limitar el acceso a TRR)	○	○	○	○

C7. Tratamiento renal conservador (TRC)
Tratamiento renal conservador: El tratamiento renal conservador se define como la elección del paciente para una asistencia integral centrada en el paciente sin el uso de Terapia de Reemplazo Renal (TRR) en los pacientes con Enfermedad Renal Crónica (ERC) estadio 5. Los objetivos del tratamiento renal conservador son apoyar a los pacientes con ERC estadio 5 que no reciben TRR, optimizando la calidad de vida, controlando los síntomas, tratando la angustia psicosocial, facilitando la planificación anticipada de la atención y, en su caso, preservando la función renal residual. Esta asistencia incluye el apoyo a los familiares y cuidadores del paciente y continúa durante toda la trayectoria de la enfermedad. Esto es adecuado en pacientes que es improbable que se beneficien de las TRR o que opten por no iniciar TRR. Reconocemos que los pacientes pueden recibir una asistencia conservadora similar cuando las limitaciones de recursos (sistema sanitario o paciente) impiden o limitan el acceso a TRR. Denominamos a esto atención conservadora con restricción de elección.

Nos gustaría saber más sobre la capacidad de ofrecer un tratamiento renal conservador o una asistencia conservadora con restricción elección en su país (es decir, los recursos para el tratamiento paliativo o de control en los pacientes que no recibirán TRR a pesar de tener ERC estadio 5).

C7.1. Teniendo en cuenta la definición mencionada, ¿existe en su país el tratamiento conservador?

C7.1.3. Atención conservadora establecida con restricción de elección (cuando no existan limitaciones de recursos para impedir o limitar el acceso a TRR)	○	○	○	○

C7.2. Indique la probabilidad media de que un nefrólogo de su país ofrezca un tratamiento renal conservador como opción de tratamiento a pacientes con ERC estadio 5.

- ○ Siempre
- ○ A menudo
- ○ A veces
- ○ Rara vez
- ○ Nunca
- ○ No lo sé

C7.3. Cuando se restringe el acceso a la diálisis, cuál es la razón principal

- ○ FEconómica - sistema sanitario
- ○ Económica - paciente
- ○ Geográfica

C7.4. Indique la disponibilidad de la estructura y el proceso para la aportación de tratamiento renal conservador (es decir, atención conservadora que se elige o se aconseja médicamente cuando el TRR está fácilmente disponible) para pacientes con ERC estadio 5:

Tenga en cuenta que significa en el 50 % o más de los centros sanitarios (hospitales o consultorios) y significa en menos del 50 % de los centros (hospitales o consultorios).

	En general disponible	En general no disponible	No disponible	No lo sé
C7.4.1. Infraestructura establecida de apoyo a los pacientes en una vía conservadora de tratamiento renal	◯	◯	◯	◯

C7.4. Indique la disponibilidad de la estructura y el proceso para la aportación de tratamiento renal conservador (es decir, atención conservadora que se elige o se aconseja médicamente cuando el TRR está fácilmente disponible) para pacientes con ERC estadio 5:

Tenga en cuenta que significa en el 50 % o más de los centros sanitarios (hospitales o consultorios) y significa en menos del 50 % de los centros (hospitales o consultorios).

C7.4.2. Herramientas de toma de decisiones compartidas para pacientes y proveedores que ayudan a tomar la decisión de un tratamiento renal conservador	◯	◯	◯	◯

C7.4. Indique la disponibilidad de la estructura y el proceso para la aportación de tratamiento renal conservador (es decir, atención conservadora que se elige o se aconseja médicamente cuando el TRR está fácilmente disponible) para pacientes con ERC estadio 5:

Tenga en cuenta que significa en el 50 % o más de los centros sanitarios (hospitales o consultorios) y significa en menos del 50 % de los centros (hospitales o consultorios).

C7.4.3. Servicios establecidos en los que los pacientes que reciben un tratamiento renal conservador pueden ser atendidos en el hogar/centro de atención/institución de cuidados si no pueden asistir a un hospital o centro médico	◯	◯	◯	◯

C7.4. Indique la disponibilidad de la estructura y el proceso para la aportación de tratamiento renal conservador (es decir, atención conservadora que se elige o se aconseja médicamente cuando el TRR está fácilmente disponible) para pacientes con ERC estadio 5:

Tenga en cuenta que significa en el 50 % o más de los centros sanitarios (hospitales o consultorios) y significa en menos del 50 % de los centros (hospitales o consultorios).

C7.4.4. Una vía, plan o guía escritos para el tratamiento renal conservador que incluya la conservación de la función renal residual, el control de los síntomas, la planificación anticipada de la atención y los cuidados terminales	○	○	○	○

C7.4. Indique la disponibilidad de la estructura y el proceso para la aportación de tratamiento renal conservador (es decir, atención conservadora que se elige o se aconseja médicamente cuando el TRR está fácilmente disponible) para pacientes con ERC estadio 5:

Tenga en cuenta que significa en el 50 % o más de los centros sanitarios (hospitales o consultorios) y significa en menos del 50 % de los centros (hospitales o consultorios).

C7.4.5. Un equipo multidisciplinar	○	○	○	○

C7.4. Indique la disponibilidad de la estructura y el proceso para la aportación de tratamiento renal conservador (es decir, atención conservadora que se elige o se aconseja médicamente cuando el TRR está fácilmente disponible) para pacientes con ERC estadio 5:

Tenga en cuenta que significa en el 50 % o más de los centros sanitarios (hospitales o consultorios) y significa en menos del 50 % de los centros (hospitales o consultorios).

C7.4.6. El equipo multidisciplinar incluye vínculos formales con nefrólogos capacitados en cuidados conservadores	◯	◯	◯	◯

C7.4. Indique la disponibilidad de la estructura y el proceso para la aportación de tratamiento renal conservador (es decir, atención conservadora que se elige o se aconseja médicamente cuando el TRR está fácilmente disponible) para pacientes con ERC estadio 5:

Tenga en cuenta que significa en el 50 % o más de los centros sanitarios (hospitales o consultorios) y significa en menos del 50 % de los centros (hospitales o consultorios).

C7.4.7. El equipo multidisciplinar incluye vínculos formales con los cuidados paliativos	○	○	○	○

C7.4. Indique la disponibilidad de la estructura y el proceso para la aportación de tratamiento renal conservador (es decir, atención conservadora que se elige o se aconseja médicamente cuando el TRR está fácilmente disponible) para pacientes con ERC estadio 5:

Tenga en cuenta que significa en el 50 % o más de los centros sanitarios (hospitales o consultorios) y significa en menos del 50 % de los centros (hospitales o consultorios).

C7.4.8. Uso habitual de herramientas de detección validadas, documentación y manejo de síntomas	○	○	○	○

C7.4. Indique la disponibilidad de la estructura y el proceso para la aportación de tratamiento renal conservador (es decir, atención conservadora que se elige o se aconseja médicamente cuando el TRR está fácilmente disponible) para pacientes con ERC estadio 5:

Tenga en cuenta que significa en el 50 % o más de los centros sanitarios (hospitales o consultorios) y significa en menos del 50 % de los centros (hospitales o consultorios).

C7.4.9. Disponibilidad de medicamentos esenciales para el dolor y los cuidados paliativos en todos los niveles de atención (primaria y especializada)	○	○	○	○

C7.4. Indique la disponibilidad de la estructura y el proceso para la aportación de tratamiento renal conservador (es decir, atención conservadora que se elige o se aconseja médicamente cuando el TRR está fácilmente disponible) para pacientes con ERC estadio 5:

Tenga en cuenta que significa en el 50 % o más de los centros sanitarios (hospitales o consultorios) y significa en menos del 50 % de los centros (hospitales o consultorios).

C7.4.10. Infraestructura para documentar y compartir conversaciones de planificación anticipada de la atención, incluidas las decisiones sobre el lugar preferido de atención y la muerte y reanimación.	○	○	○	○

C7.4. Indique la disponibilidad de la estructura y el proceso para la aportación de tratamiento renal conservador (es decir, atención conservadora que se elige o se aconseja médicamente cuando el TRR está fácilmente disponible) para pacientes con ERC estadio 5:

Tenga en cuenta que significa en el 50 % o más de los centros sanitarios (hospitales o consultorios) y significa en menos del 50 % de los centros (hospitales o consultorios).

C7.4.11. Prestación de apoyo psicológico, social y espiritual	○	○	○	○

C7.4. Indique la disponibilidad de la estructura y el proceso para la aportación de tratamiento renal conservador (es decir, atención conservadora que se elige o se aconseja médicamente cuando el TRR está fácilmente disponible) para pacientes con ERC estadio 5:

Tenga en cuenta que significa en el 50 % o más de los centros sanitarios (hospitales o consultorios) y significa en menos del 50 % de los centros (hospitales o consultorios).

C7.4.12. Formación de los profesionales sanitarios en el tratamiento de los síntomas	○	○	○	○

C7.4. Indique la disponibilidad de la estructura y el proceso para la aportación de tratamiento renal conservador (es decir, atención conservadora que se elige o se aconseja médicamente cuando el TRR está fácilmente disponible) para pacientes con ERC estadio 5:

Tenga en cuenta que significa en el 50 % o más de los centros sanitarios (hospitales o consultorios) y significa en menos del 50 % de los centros (hospitales o consultorios).

C7.4.13. Capacitación de los proveedores de atención en la planificación anticipada de la atención	◯	◯	◯	◯

C7.4. Indique la disponibilidad de la estructura y el proceso para la aportación de tratamiento renal conservador (es decir, atención conservadora que se elige o se aconseja médicamente cuando el TRR está fácilmente disponible) para pacientes con ERC estadio 5:

Tenga en cuenta que significa en el 50 % o más de los centros sanitarios (hospitales o consultorios) y significa en menos del 50 % de los centros (hospitales o consultorios).

C7.4.14. Recogida sistemática de datos sobre el número de pacientes que reciben un tratamiento renal conservador y sus resultados	○	○	○	○

C7.5. Indique la disponibilidad de la estructura y el proceso para la prestación de asistencia conservadora con restricción de elección (es decir, asistencia conservadora para pacientes en los que las limitaciones de recursos impiden o limitan el acceso a TRR) en pacientes con ERC estadio 5):

Tenga en cuenta que significa en el 50 % o más de los centros sanitarios (hospitales o consultorios) y significa en menos del 50 % de los centros (hospitales o consultorios).

	En general disponible	En general no disponible	No disponible	No lo sé
C7.5.1. Infraestructura establecida para apoyar a los pacientes que reciben atención conservadora con restricción de la elección	○	○	○	○

C7.5. Indique la disponibilidad de la estructura y el proceso para la prestación de asistencia conservadora con restricción de elección (es decir, asistencia conservadora para pacientes en los que las limitaciones de recursos impiden o limitan el acceso a TRR) en pacientes con ERC estadio 5):

Tenga en cuenta que significa en el 50 % o más de los centros sanitarios (hospitales o consultorios) y significa en menos del 50 % de los centros (hospitales o consultorios).

C7.5.2. Servicios establecidos en los que los pacientes que reciben atención conservadora con restricción de elección pueden ser atendidos en el hogar/residencia/institución de cuidados mínimos si no pueden asistir a un hospital o centro médico	○	○	○	○

C7.5. Indique la disponibilidad de la estructura y el proceso para la prestación de asistencia conservadora con restricción de elección (es decir, asistencia conservadora para pacientes en los que las limitaciones de recursos impiden o limitan el acceso a TRR) en pacientes con ERC estadio 5):

Tenga en cuenta que significa en el 50 % o más de los centros sanitarios (hospitales o consultorios) y significa en menos del 50 % de los centros (hospitales o consultorios).

C7.5.3. Una vía, plan o guía escritos para el tratamiento conservador que incluya la conservación de la función renal residual, el control de los síntomas, la planificación anticipada de la atención y los cuidados terminales	○	○	○	○

C7.5. Indique la disponibilidad de la estructura y el proceso para la prestación de asistencia conservadora con restricción de elección (es decir, asistencia conservadora para pacientes en los que las limitaciones de recursos impiden o limitan el acceso a TRR) en pacientes con ERC estadio 5):

Tenga en cuenta que significa en el 50 % o más de los centros sanitarios (hospitales o consultorios) y significa en menos del 50 % de los centros (hospitales o consultorios).

C7.5.4. Un equipo multidisciplinar	○	○	○	○

C7.5. Indique la disponibilidad de la estructura y el proceso para la prestación de asistencia conservadora con restricción de elección (es decir, asistencia conservadora para pacientes en los que las limitaciones de recursos impiden o limitan el acceso a TRR) en pacientes con ERC estadio 5):

Tenga en cuenta que significa en el 50 % o más de los centros sanitarios (hospitales o consultorios) y significa en menos del 50 % de los centros (hospitales o consultorios).

C7.5.5. El equipo multidisciplinar incluye vínculos formales con nefrólogos capacitados en cuidados conservadores	○	○	○	○

C7.5. Indique la disponibilidad de la estructura y el proceso para la prestación de asistencia conservadora con restricción de elección (es decir, asistencia conservadora para pacientes en los que las limitaciones de recursos impiden o limitan el acceso a TRR) en pacientes con ERC estadio 5):

Tenga en cuenta que significa en el 50 % o más de los centros sanitarios (hospitales o consultorios) y significa en menos del 50 % de los centros (hospitales o consultorios).

C7.5.6. El equipo multidisciplinar incluye vínculos formales con los cuidados paliativos	◯	◯	◯	◯

C7.5. Indique la disponibilidad de la estructura y el proceso para la prestación de asistencia conservadora con restricción de elección (es decir, asistencia conservadora para pacientes en los que las limitaciones de recursos impiden o limitan el acceso a TRR) en pacientes con ERC estadio 5):

Tenga en cuenta que significa en el 50 % o más de los centros sanitarios (hospitales o consultorios) y significa en menos del 50 % de los centros (hospitales o consultorios).

C7.5.7. Uso habitual de herramientas de detección validadas, documentación y manejo de síntomas	○	○	○	○

C7.5. Indique la disponibilidad de la estructura y el proceso para la prestación de asistencia conservadora con restricción de elección (es decir, asistencia conservadora para pacientes en los que las limitaciones de recursos impiden o limitan el acceso a TRR) en pacientes con ERC estadio 5):

Tenga en cuenta que significa en el 50 % o más de los centros sanitarios (hospitales o consultorios) y significa en menos del 50 % de los centros (hospitales o consultorios).

C7.5.8. Disponibilidad de medicamentos esenciales para el dolor y los cuidados paliativos en todos los niveles de atención (primaria y especializada)	○	○	○	○

C7.5. Indique la disponibilidad de la estructura y el proceso para la prestación de asistencia conservadora con restricción de elección (es decir, asistencia conservadora para pacientes en los que las limitaciones de recursos impiden o limitan el acceso a TRR) en pacientes con ERC estadio 5):

Tenga en cuenta que significa en el 50 % o más de los centros sanitarios (hospitales o consultorios) y significa en menos del 50 % de los centros (hospitales o consultorios).

C7.5.9. Infraestructura para documentar y compartir conversaciones de planificación anticipada de la atención, incluidas las decisiones sobre el lugar preferido de atención y la muerte y reanimación.	○	○	○	○

C7.5. Indique la disponibilidad de la estructura y el proceso para la prestación de asistencia conservadora con restricción de elección (es decir, asistencia conservadora para pacientes en los que las limitaciones de recursos impiden o limitan el acceso a TRR) en pacientes con ERC estadio 5):

Tenga en cuenta que significa en el 50 % o más de los centros sanitarios (hospitales o consultorios) y significa en menos del 50 % de los centros (hospitales o consultorios).

C7.5.10. Prestación de apoyo psicológico, social y espiritual	○	○	○	○

C7.5. Indique la disponibilidad de la estructura y el proceso para la prestación de asistencia conservadora con restricción de elección (es decir, asistencia conservadora para pacientes en los que las limitaciones de recursos impiden o limitan el acceso a TRR) en pacientes con ERC estadio 5):

Tenga en cuenta que significa en el 50 % o más de los centros sanitarios (hospitales o consultorios) y significa en menos del 50 % de los centros (hospitales o consultorios).

C7.5.11. Formación de los profesionales sanitarios en el tratamiento de los síntomas	○	○	○	○

C7.5. Indique la disponibilidad de la estructura y el proceso para la prestación de asistencia conservadora con restricción de elección (es decir, asistencia conservadora para pacientes en los que las limitaciones de recursos impiden o limitan el acceso a TRR) en pacientes con ERC estadio 5):

Tenga en cuenta que significa en el 50 % o más de los centros sanitarios (hospitales o consultorios) y significa en menos del 50 % de los centros (hospitales o consultorios).

C7.5.12. Capacitación de los proveedores de atención en la planificación anticipada de la atención	○	○	○	○

C7.5. Indique la disponibilidad de la estructura y el proceso para la prestación de asistencia conservadora con restricción de elección (es decir, asistencia conservadora para pacientes en los que las limitaciones de recursos impiden o limitan el acceso a TRR) en pacientes con ERC estadio 5):

Tenga en cuenta que significa en el 50 % o más de los centros sanitarios (hospitales o consultorios) y significa en menos del 50 % de los centros (hospitales o consultorios).

C7.5.13. Recogida sistemática de datos sobre el número de pacientes que reciben tratamiento conservador con restricción de la elección y sus resultados	◯	◯	◯	◯

C8. Asequibilidad	
C8.1. ¿Cuál es el promedio nacional de copago en su país (incluidos los medicamentos, pero no otros complementos) para los pacientes en hemodiálisis, es decir, qué porcentaje del precio del tratamiento paga directamente (de su bolsillo) el paciente?	◯ N/A (no existe en mi país)<br>◯ 0 %<br>◯ 1-25 %<br>◯ 26-50 %<br>◯ 51-75 %<br>◯ >75 %<br>◯ 100 %
C8.1.1. ¿Varía este porcentaje en diferentes partes del país?	◯ Sí (explíquelo a continuación)<br>◯ No<br>◯ Otros (explíquelo a continuación)
C8.1.1. Si ha seleccionado "si" por favor especifique.	__________________________________
C8.1.1. Si ha seleccionado "otros" por favor especifique.	__________________________________
C8.1.2. ¿Varía este porcentaje en función de las características de los pacientes (por ejemplo, la edad, el género o la situación laboral)?	◯ Sí (explíquelo a continuación)<br>◯ No<br>◯ Otros (explíquelo a continuación)
C8.1.2. Si ha seleccionado "si" por favor especifique.	__________________________________
C8.1.2. Si ha seleccionado "otros" por favor especifique.	__________________________________
C8.1.3. ¿Qué proporción (media nacional) de pacientes con ERC estadio 5 en hemodiálisis abandonan la diálisis en el plazo de un año por razones económicas en su país?	◯ N/A (no existe en mi país)<br>◯ 0 %<br>◯ 1-10 %<br>◯ 11-25 %<br>◯ 26-50 %<br>◯ >50 %
C8.2. ¿Cuál es el promedio nacional de copago en su país (incluidos los medicamentos, pero no otros complementos) para los pacientes en diálisis peritoneal, es decir, qué porcentaje del precio del tratamiento paga directamente (de su bolsillo) el paciente?	◯ N/A (no existe en mi país)<br>◯ 0 %<br>◯ 1-25 %<br>◯ 26-50 %<br>◯ 51-75 %<br>◯ >75 %<br>◯ 100 %
C8.2.1. ¿Varía este porcentaje en diferentes partes del país?	◯ Sí (explíquelo a continuación)<br>◯ No<br>◯ Otros (explíquelo a continuación)
C8.2.1. Si ha seleccionado "si" por favor especifique.	__________________________________

C8.2.1. Si ha seleccionado "otros" por favor especifique.	__________________
C8.2.2. ¿Varía este porcentaje en función de las características de los pacientes (por ejemplo, la edad, el género o la situación laboral)?	◯ Sí (explíquelo a continuación)<br>◯ No<br>◯ Otros (explíquelo a continuación)
C8.2.2. Si ha seleccionado "si" por favor especifique.	__________________
C8.2.2. Si ha seleccionado "otros" por favor especifique.	__________________
C8.3. ¿Cuál es el promedio nacional de copago en su país (incluidos los medicamentos, pero no otros complementos) para los pacientes sometidos a un trasplante de riñón, es decir, qué porcentaje del precio del tratamiento paga directamente (de su bolsillo) el paciente?	◯ N/A (no existe en mi país)<br>◯ 0 %<br>◯ 1-25 %<br>◯ 26-50 %<br>◯ 51-75 %<br>◯ >75 %<br>◯ 100 %
C8.3.1. ¿Varía este porcentaje en diferentes partes del país?	◯ Sí (explíquelo a continuación)<br>◯ No<br>◯ Otros (explíquelo a continuación)
C8.3.1. Si ha seleccionado "si" por favor especifique.	__________________
C8.3.1. Si ha seleccionado "otros" por favor especifique.	__________________
C8.3.2. ¿Varía este porcentaje en función de las características de los pacientes (por ejemplo, la edad, el género o la situación laboral)?	◯ Sí (explíquelo a continuación)<br>◯ No<br>◯ Otros (explíquelo a continuación)
C8.3.2. Si ha seleccionado "si" por favor especifique.	__________________
C8.3.2. Si ha seleccionado "otros" por favor especifique.	__________________
C8.4. ¿Qué porcentaje (promedio nacional) de los pacientes con ERC estadio 5 pueden acceder a la diálisis en su país?	◯ N/A (no existe en mi país)<br>◯ 1-10 %<br>◯ 11-25 %<br>◯ 26-50 %<br>◯ >50 %
C8.4.1. ¿Varía este porcentaje en diferentes partes del país?	◯ Sí (explíquelo a continuación)<br>◯ No<br>◯ Otros (explíquelo a continuación)

C8.4.1. Si ha seleccionado "si" por favor especifique.

____

C8.4.1. Si ha seleccionado "otros" por favor especifique.

____

C8.4.2. ¿Varía este porcentaje en función de las características de los pacientes (por ejemplo, la edad, el género o la situación laboral)?

- ◯ Sí (explíquelo a continuación)
- ◯ No
- ◯ Otros (explíquelo a continuación)

C8.4.2. Si ha seleccionado "si" por favor especifique.

____

C8.4.2. Si ha seleccionado "otros" por favor especifique.

____

C8.5. De los pacientes de su país que padecen ERC estadio 5 y pueden acceder a la diálisis, ¿qué porcentaje suelen empezar con la diálisis peritoneal?

- ◯ N/A; en mi país no existe la diálisis (de ningún tipo)
- ◯ 0 % (es decir, hay pacientes que pueden acceder a algún tipo de diálisis, pero ninguno empieza con la DP)
- ◯ 1-10 %
- ◯ 11-25 %
- ◯ 26-50 %
- ◯ >50 %

C8.5.1. ¿Varía este porcentaje en diferentes partes del país?

- ◯ Sí (explíquelo a continuación)
- ◯ No
- ◯ Otros (explíquelo a continuación)

C8.5.1. Si ha seleccionado "si" por favor especifique.

____

C8.5.1. Si ha seleccionado "otros" por favor especifique.

____

C8.5.2. ¿Varía este porcentaje en función de las características de los pacientes (por ejemplo, la edad, el género o la situación laboral)?

- ◯ Sí (explíquelo a continuación)
- ◯ No
- ◯ Otros (explíquelo a continuación)

C8.5.2. Si ha seleccionado "si" por favor especifique.

____

C8.5.2. Si ha seleccionado "otros" por favor especifique.

____

C8.6. De los pacientes de su país que padecen ERC estadio 5 y son aptos para el trasplante, ¿qué porcentaje pueden acceder a un trasplante de riñón?	◯ 0% (no existe en mi país)<br>◯ 1-10%<br>◯ 11-25%<br>◯ 26-50%<br>◯ >50%
C8.6.1. ¿Varía este porcentaje en diferentes partes del país?	◯ Sí (explíquelo a continuación)<br>◯ No<br>◯ Otros (explíquelo a continuación)
C8.6.1. Si ha seleccionado "si" por favor especifique.	__________________________________
C8.6.1. Si ha seleccionado "otros" por favor especifique.	__________________________________
C8.6.2. ¿Varía este porcentaje en función de las características de los pacientes (por ejemplo, la edad, el género o la situación laboral)?	◯ Sí (explíquelo a continuación)<br>◯ No<br>◯ Otros (explíquelo a continuación)
C8.6.2. Si ha seleccionado "si" por favor especifique.	__________________________________
C8.6.2. Si ha seleccionado "otros" por favor especifique.	__________________________________

C9.

Calidad de la diálisis peritoneal
Si en su país existe la diálisis peritoneal, ¿qué porcentaje de los centros determinan y notifican de forma sistemática los parámetros que se indican para evaluar la calidad de la diálisis realizada?

C9.1. ¿Qué proporción de pacientes en DP comienza con una DP inferior a la dosis completa (es decir, incremental)? (menos de la dosis completa se considera < 8 l/día)	◯ 0%<br>◯ 1-10%<br>◯ 11-25%<br>◯ 26-50%<br>◯ >50%
C9.2. Parámetros de valoración notificados por los pacientes (por ejemplo, cansancio, calidad de vida, satisfacción, dolor):	◯ 0% (ninguno)<br>◯ 1-10% (pocos)<br>◯ 11-50% (algunos)<br>◯ 51-75% (la mayoría)<br>◯ >75% (casi todos)
C9.3. Presión arterial:	◯ 0% (ninguno)<br>◯ 1-10% (pocos)<br>◯ 11-50% (algunos)<br>◯ 51-75% (la mayoría)<br>◯ >75% (casi todos)
C9.4. Aclaramiento de solutos de bajo peso molecular (por ejemplo, Kt/V o aclaramiento de creatinina)	◯ 0% (ninguno)<br>◯ 1-10% (pocos)<br>◯ 11-50% (algunos)<br>◯ 51-75% (la mayoría)<br>◯ >75% (casi todos)
C9.5. Hemoglobina o hematocrito:	◯ 0% (ninguno)<br>◯ 1-10% (pocos)<br>◯ 11-50% (algunos)<br>◯ 51-75% (la mayoría)<br>◯ >75% (casi todos)
C9.6. Marcadores de metabolismo mineral y óseo (calcio, fosfato, hormona paratiroidea [PTH]):	◯ 0% (ninguno)<br>◯ 1-10% (pocos)<br>◯ 11-50% (algunos)<br>◯ 51-75% (la mayoría)<br>◯ >75% (casi todos)
C9.7. Supervivencia de la técnica (transferencia a HD):	◯ 0% (ninguno)<br>◯ 1-10% (pocos)<br>◯ 11-50% (algunos)<br>◯ 51-75% (la mayoría)<br>◯ >75% (casi todos)
C9.8. Supervivencia del paciente:	◯ 0% (ninguno)<br>◯ 1-10% (pocos)<br>◯ 11-50% (algunos)<br>◯ 51-75% (la mayoría)<br>◯ >75% (casi todos)

C9.9. ¿Cuál es la relación personal de enfermería/paciente por turno en ≥ 50 % de los centros de diálisis peritoneal de su país?

- ◯ de 1:1 a 1:3
- ◯ de 1:4 a 1:6
- ◯ de 1:7 a 1:9
- ◯ de 1:10 a 1:13
- ◯ ≥1:14
- ◯ No lo sé

C10. Calidad de la hemodiálisis
Si en su país existe la hemodiálisis, ¿qué porcentaje de los centros determinan y notifican de forma sistemática los parámetros que se indican para evaluar la calidad de la diálisis realizada?

C10.1. ¿Qué porcentaje de los pacientes con HD comienza con menos de 3 veces/semana de HD (es decir, HD incremental)?	◯ 0%<br>◯ 1-10%<br>◯ 11-25%<br>◯ 26-50%<br>◯ >50%
C10.2. Parámetros de valoración notificados por los pacientes (por ejemplo, cansancio, calidad de vida, satisfacción, dolor, etc.):	◯ 0% (ninguno)<br>◯ 1-10% (pocos)<br>◯ 11-50% (algunos)<br>◯ 51-75% (la mayoría)<br>◯ >75% (casi todos)
C10.3. Presión arterial:	◯ 0% (ninguno)<br>◯ 1-10% (pocos)<br>◯ 11-50% (algunos)<br>◯ 51-75% (la mayoría)<br>◯ >75% (casi todos)
C10.4. Aclaramiento de solutos de bajo peso molecular (por ejemplo, Kt/V o aclaramiento de creatinina):	◯ 0% (ninguno)<br>◯ 1-10% (pocos)<br>◯ 11-50% (algunos)<br>◯ 51-75% (la mayoría)<br>◯ >75% (casi todos)
C10.5. Hemoglobina o hematocrito:	◯ 0% (ninguno)<br>◯ 1-10% (pocos)<br>◯ 11-50% (algunos)<br>◯ 51-75% (la mayoría)<br>◯ >75% (casi todos)
C10.6. Marcadores de minerales óseos (calcio, fosfato, PTH):	◯ 0% (ninguno)<br>◯ 1-10% (pocos)<br>◯ 11-50% (algunos)<br>◯ 51-75% (la mayoría)<br>◯ >75% (casi todos)
C10.7. Supervivencia de la técnica:	◯ 0% (ninguno)<br>◯ 1-10% (pocos)<br>◯ 11-50% (algunos)<br>◯ 51-75% (la mayoría)<br>◯ >75% (casi todos)
C10.8. Supervivencia del paciente:	◯ 0% (ninguno)<br>◯ 1-10% (pocos)<br>◯ 11-50% (algunos)<br>◯ 51-75% (la mayoría)<br>◯ >75% (casi todos)
C10.9. Control de la hepatitis B y C y virología del VIH al menos dos veces al año:	◯ 0% (ninguno)<br>◯ 1-10% (pocos)<br>◯ 11-50% (algunos)<br>◯ 51-75% (la mayoría)<br>◯ >75% (casi todos)

C10.10. Control periódico de la calidad del agua de diálisis en cuanto a bacterias y componentes químicos según la AAMI o la recomendación del organismo regulador nacional equivalente:	◯ 0% (ninguno)<br>◯ 1-10% (pocos)<br>◯ 11-50% (algunos)<br>◯ 51-75% (la mayoría)<br>◯ >75% (casi todos)
C10.11. Revisión periódica de pacientes en HD por el nefrólogo al menos una vez cada 3 meses:	◯ 0% (ninguno)<br>◯ 1-10% (pocos)<br>◯ 11-50% (algunos)<br>◯ 51-75% (la mayoría)<br>◯ >75% (casi todos)
C10.12. ¿Cuál es la relación personal de enfermería/paciente por turno en ≥ 50 % de los centros de hemodiálisis de su país?	◯ de 1:1 a 1:3<br>◯ de 1:4 a 1:6<br>◯ de 1:7 a 1:9<br>◯ de 1:10 a 1:13<br>◯ ≥1:14<br>◯ No lo sé

C11. Calidad del trasplante de riñón

Si en su país existe el trasplante de riñón, ¿qué porcentaje de los centros determinan y notifican de forma sistemática los parámetros que se indican para evaluar la calidad del trasplante realizado?

C11.1. Parámetros de valoración notificados por los pacientes (por ejemplo, cansancio, calidad de vida, satisfacción, dolor, etc.):

◯ 0% (ninguno)
◯ 1-10% (pocos)
◯ 11-50% (algunos)
◯ 51-75% (la mayoría)
◯ >75% (casi todos)
◯ No lo sé

C11.2. Función retrasada del injerto:

◯ 0% (ninguno)
◯ 1-10% (pocos)
◯ 11-50% (algunos)
◯ 51-75% (la mayoría)
◯ >75% (casi todos)
◯ No lo sé

C11.3. Porcentaje o incidencia de rechazo:

◯ 0% (ninguno)
◯ 1-10% (pocos)
◯ 11-50% (algunos)
◯ 51-75% (la mayoría)
◯ >75% (casi todos)
◯ No lo sé

C11.4. Función del aloinjerto renal:

◯ 0% (ninguno)
◯ 1-10% (pocos)
◯ 11-50% (algunos)
◯ 51-75% (la mayoría)
◯ >75% (casi todos)
◯ No lo sé

C11.5. Supervivencia del injerto:

◯ 0% (ninguno)
◯ 1-10% (pocos)
◯ 11-50% (algunos)
◯ 51-75% (la mayoría)
◯ >75% (casi todos)
◯ No lo sé

C11.6. Supervivencia del paciente:

◯ 0% (ninguno)
◯ 1-10% (pocos)
◯ 11-50% (algunos)
◯ 51-75% (la mayoría)
◯ >75% (casi todos)
◯ No lo sé

C12. Acceso Vascular

C12.1. Porcentaje de los pacientes que empiezan de forma sistemática la hemodiálisis con un acceso vascular permanente funcionante (fístula AV o prótesis):

- ◯ 0% (ninguno)
- ◯ 1-10% (pocos)
- ◯ 11-50% (algunos)
- ◯ 51-75% (la mayoría)
- ◯ >75% (casi todos)
- ◯ No lo sé

C12.2. Porcentaje de los pacientes que empiezan de forma sistemática la hemodiálisis con un catéter de diálisis tunelizado:

- ◯ 0% (ninguno)
- ◯ 1-10% (pocos)
- ◯ 11-50% (algunos)
- ◯ 51-75% (la mayoría)
- ◯ >75% (casi todos)
- ◯ No lo sé

C12.3. Porcentaje de los pacientes que suelen empezar la hemodiálisis con un catéter de diálisis no tunelizado (temporal):

- ◯ 0% (ninguno)
- ◯ 1-10% (pocos)
- ◯ 11-50% (algunos)
- ◯ 51-75% (la mayoría)
- ◯ >75% (casi todos)
- ◯ No lo sé

C12.4. En hemodiálisis, ¿qué proporción de pacientes prevalentes se dializa con un acceso vascular funcional (fístula AV o injerto)?

- ◯ 0% (ninguno)
- ◯ 1-10% (pocos)
- ◯ 11-50% (algunos)
- ◯ 51-75% (la mayoría)
- ◯ >75% (casi todos)
- ◯ No lo sé

Acceso para todos los tipos de diálisis; responda solo si en su país existe la hemodiálisis o la diálisis peritoneal

C12.5. Porcentaje de los pacientes en hemodiálisis o en diálisis peritoneal que reciben de forma sistemática formación sobre las mejores formas de acceso y la cirugía a tiempo (por ejemplo, seis meses antes del inicio de la hemodiálisis, un mes antes del inicio de la diálisis peritoneal):

- ◯ 0% (ninguno)
- ◯ 1-10% (pocos)
- ◯ 11-50% (algunos)
- ◯ 51-75% (la mayoría)
- ◯ >75% (casi todos)
- ◯ No lo sé

C13. Resultados (hemodiálisis)

C13.1. ¿Qué proporción (media nacional) de pacientes con ERC estadio 5 en hemodiálisis murieron en el primer año de diálisis (mortalidad en el primer año) en su país?

- ◯ 0% (ninguno)
- ◯ 1-10% (pocos)
- ◯ 11-50% (algunos)
- ◯ 51-75% (la mayoría)
- ◯ >75% (casi todos)
- ◯ No lo sé

C13.2. ¿Cuál es la causa más común de muerte entre los pacientes de hemodiálisis en su país?

- ◯ Enfermedad cardiovascular (cardiopatía isquémica, arritmias, enfermedad cerebrovascular)
- ◯ Infección (infección relacionada con el acceso, FAV/IAV infectados, bacteriemia relacionada con el catéter)
- ◯ Infección (otras fuentes, neumonía, gangrena de extremidades, etc.)
- ◯ Neoplasias malignas
- ◯ Retirar la diálisis (por motivos sociales)
- ◯ Retirar la diálisis (debido al coste de la atención)
- ◯ Otras (especifique)
- ◯ No lo sé

C13.2. Si ha seleccionado "otros" por favor especifique.

____

C13.3. ¿Qué proporción (media nacional) de pacientes con ERC estadio 5 en hemodiálisis requiere al menos una hospitalización en el primer año de diálisis (hospitalización del primer año) en su país?

- ◯ 1-10%
- ◯ 11-20%
- ◯ 21-30%
- ◯ 31-50%
- ◯ >50%
- ◯ No lo sé

C13.4. ¿Cuál es la causa más frecuente de hospitalización entre los pacientes en hemodiálisis de su país?

- ◯ Enfermedad cardiovascular (cardiopatía isquémica, arritmias, enfermedad cerebrovascular)
- ◯ Fallo de acceso (FAV/IAV defectuoso o catéter venoso central bloqueado)
- ◯ Infección relacionada con el acceso (infección de FAV/IAV, bacteriemia relacionada con el catéter CVC)
- ◯ Infección (otros focos, neumonía, gangrena de extremidades, etc.)
- ◯ Otras (especifique)
- ◯ No lo sé

C13.4. Si ha seleccionado "otros" por favor especifique.

____

C14. Resultados (diálisis peritoneal)

C14.1. ¿Qué proporción (media nacional) de pacientes con ERC estadio 5 en diálisis peritoneal murieron en el primer año de diálisis (mortalidad en el primer año) en su país?

- ◯ 1-10%
- ◯ 11-20%
- ◯ 21-30%
- ◯ 31-50%
- ◯ >50%
- ◯ No lo sé

C14.2.¿Cuál es la causa más común de muerte entre los pacientes de diálisis peritoneal en su país?

- ◯ Enfermedad cardiovascular (cardiopatía isquémica, arritmias, enfermedad cerebrovascular)
- ◯ Infección relacionada con la diálisis peritoneal (peritonitis relacionada con diálisis peritoneal, infección del sitio de salida o del túnel)
- ◯ Infección (otros focos, neumonía, gangrena de extremidades, etc.)
- ◯ Neoplasias malignas
- ◯ Retiro del programa de diálisis (por razones sociales)
- ◯ Retiro del programa de diálisis (por el coste del servicio médico)
- ◯ Otras (especifique)
- ◯ No lo sé

C14.2. Si ha seleccionado "otros" por favor especifique.

____

C14.3 ¿Qué proporción (media nacional) de pacientes con ERC estadio 5 en diálisis peritoneal requieren al menos una hospitalización en el primer año de diálisis (hospitalización del primer año) en su país?

- ◯ 1-10%
- ◯ 11-20%
- ◯ 21-30%
- ◯ 31-50%
- ◯ >50%
- ◯ No lo sé

C14.4. ¿Cuál es la causa más frecuente de hospitalización entre los pacientes sometidos a diálisis peritoneal en su país?

- ◯ Enfermedad cardiovascular (cardiopatía isquémica, arritmias, enfermedad cerebrovascular)
- ◯ Fallo del acceso (bloqueo del catéter de DP, migración de la punta del catéter)
- ◯ Infección relacionada con DP (peritonitis, infección en el sitio de salida o en el túnel)
- ◯ Infección (otros focos, neumonía, gangrena de extremidades, etc.)
- ◯ Otras (especifique)
- ◯ No lo sé

C14.4. Si ha seleccionado "otros" por favor especifique.

____

C15. Datos demográficos

C15.1. ¿Qué proporción (media nacional) de pacientes con ERC estadio 5 tienen más de 65 años en su país?

- ◯ 0%
- ◯ 1-10%
- ◯ 11-25%
- ◯ 26-50%
- ◯ >50%
- ◯ No lo sé

C15.2. ¿Qué proporción (media nacional) de pacientes con ERC estadio 5 son mujeres en su país?

- ◯ 0%
- ◯ 1-10%
- ◯ 11-25%
- ◯ 26-50%
- ◯ >50%
- ◯ No lo sé

C16. Etiología de la insuficiencia renal (ERT)

C16.1. ¿Cuál es la causa más frecuente de ERC estadio 5 en su país?

- ◯ Nefropatia diabetica
- ◯ Enfermedad renal poliquística
- ◯ Hipertensión arterial
- ◯ Glomerulonefritis
- ◯ Otras (especifique)
- ◯ No lo sé

C16.1. Si ha seleccionado "otros" por favor especifique.

____

C16.2. ¿Qué proporción (media nacional) de pacientes con ERC estadio 5 se debe a la enfermedad renal diabética en su país?

- ◯ 0%
- ◯ 1-25%
- ◯ 26-50%
- ◯ 51-75%
- ◯ >75%
- ◯ No lo sé

C16.3. ¿Qué proporción (media nacional) de pacientes con ERC estadio 5 se debe a glomerulonefritis en su país?

- ◯ 0%
- ◯ 1-25%
- ◯ 26-50%
- ◯ 51-75%
- ◯ >75%
- ◯ No lo sé

C16.4. ¿Qué proporción (media nacional) de pacientes con ERC estadio 5 se debe a enfermedad renal poliquística en su país?

- ◯ 0%
- ◯ 1-25%
- ◯ 26-50%
- ◯ 51-75%
- ◯ >75%
- ◯ No lo sé

C16.5 ¿Qué proporción (media nacional) de pacientes con ERC estadio 5 se debe a enfermedad renal hipertensiva en su país?

- ◯ 0%
- ◯ 1-25%
- ◯ 26-50%
- ◯ 51-75%
- ◯ >75%
- ◯ No lo sé

C17 Uso de tecnología en la atención a las enfermedades renales

C17.1. ¿Hay capacidad para las revisiones de telemedicina/telenefrología para el tratamiento de la ERC y la ERC estadio 5 en su país?	◯ Sí (si es posible, indique brevemente los detalles)<br>◯ No<br>◯ No lo sé
C17.1. Si ha seleccionado "si" por favor especifique.	__________________________________
C17.2. ¿Tienen los pacientes la opción de recibir comunicaciones (es decir, resultados de pruebas, recordatorios de citas) de centros médicos/hospitales por mensaje de texto o correo electrónico?	◯ Sí (si es posible, indique brevemente los detalles)<br>◯ No<br>◯ No lo sé
C17.2. Si ha seleccionado "si" por favor especifique.	__________________________________
C17.3. ¿Hay financiación/reembolso para los proveedores que proporcionan consultas de telemedicina/telenefrología por ERC y ERC estadio 5?	◯ Financiada por el estado y gratuita para el usuario<br>◯ Financiada por el estado, pero con copago para el usuario<br>◯ Un sistema combinado de financiación pública (sea o no gratuita para el usuario) y privada (explíquelo)<br>◯ Financiación exclusivamente privada y de pago directo<br>◯ Financiación exclusivamente privada a través de compañías de seguros<br>◯ Varios sistemas: programas estatales, de ONGs y de colectivos<br>◯ N/A (no aplica). En mi país no existe la telemedicina/telenefrología<br>◯ Otra (especifique)
C17.3. Si se trata de una mezcla entre financiación pública y privada, explique.	__________________________________
C17.3. Si ha seleccionado "otra" por favor especifique	__________________________________

C18 Gestión de desastres/Poblaciones vulnerables

C18.1. ¿Existen directrices sobre las medidas que deben adoptarse para la preparación ante catástrofes (es decir, en los centros de diálisis) en caso de terremoto, inundación o sequía en su país?	◯ Sí (si es posible, indique brevemente los detalles)<br>◯ No<br>◯ No lo sé
C18.1. Si ha seleccionado "si" por favor especifique.	__________________________________
C18.2. ¿Tiene su país un representante en el Grupo de Trabajo de Alivio de Desastres Renales?	◯ Sí (si es posible, indique brevemente los detalles)<br>◯ No<br>◯ No lo sé
C18.2. Si ha seleccionado "si" por favor especifique.	__________________________________
C18.3. ¿Existen medios para identificar a las poblaciones vulnerables (es decir, personas con inseguridad habitacional, minorías raciales/étnicas, personas que viven en la pobreza, personas con inseguridad alimentaria) en su país?	◯ Sí (si es posible, indique brevemente los detalles)<br>◯ No<br>◯ No lo sé
C18.3. Si ha seleccionado "si" por favor especifique.	__________________________________

C18.4. ¿Qué describe mejor su estructura de financiación del sistema sanitario para el tratamiento de la ERC y la ERC estadio 5 en poblaciones de refugiados?

Reduzca el tamaño de la letra si el texto de abajo se superpone

	Financiados por el estado y gratuitos para el usuario	Financiados por el estado, pero con copago para el usuario	Una combinación de sistemas de financiación pública (sea o no gratuita para el usuario) y privada (especifique)	Financiación exclusivamente privada y de pago directo	Financiación exclusivamente privada a través de compañías de seguros	Varios sistemas: programas estatales, de ONGs y de colectivos	Otro (especifique)	N/A (los refugiados no suelen tener acceso al tratamiento de la enfermedad renal)
C18.4.1. Hemodiálisis (algunos o todos los aspectos de ella)	○	○	○	○	○	○	○	○

C18.4. ¿Qué describe mejor su estructura de financiación del sistema sanitario para el tratamiento de la ERC y la ERC estadio 5 en poblaciones de refugiados?

Reduzca el tamaño de la letra si el texto de abajo se superpone

C18.4.2. Diálisis peritoneal (algunos o todos los aspectos de ella)	○	○	○	○	○	○	○	○

C18.4. ¿Qué describe mejor su estructura de financiación del sistema sanitario para el tratamiento de la ERC y la ERC estadio 5 en poblaciones de refugiados?

Reduzca el tamaño de la letra si el texto de abajo se superpone

C18.4.3. Trasplante renal (algunos o todos los aspectos de él)	◯	◯	◯	◯	◯	◯	◯	◯

C18.4. ¿Qué describe mejor su estructura de financiación del sistema sanitario para el tratamiento de la ERC y la ERC estadio 5 en poblaciones de refugiados?

Reduzca el tamaño de la letra si el texto de abajo se superpone

C18.4.4. Cuidados conservadores (algunos o todos los aspectos de él) ○ ○ ○ ○ ○ ○ ○ ○

C18.4.1. Si ha seleccionado "otros" por favor especifique. __________________________________

C18.4.1. Si se trata de una mezcla entre financiación pública y privada, explique. __________________________________

C18.4.2. Si ha seleccionado "otros" por favor especifique. __________________________________

C18.4.2. Si se trata de una mezcla entre financiación pública y privada, explique. __________________________________

C18.4.3. Si ha seleccionado "otros" por favor especifique. __________________________________

C18.4.3. Si se trata de una mezcla entre financiación pública y privada, explique. __________________________________

C18.4.4. Si ha seleccionado "otros" por favor especifique. __________________________________

C18.4.4. Si se trata de una mezcla entre financiación pública y privada, explique. __________________________________

D. Sistemas de información sanitaria y estadísticas
D1. Registros

Definiciones y abreviaturas

Registro: Recopilación sistemática de datos para evaluar resultados específicos en una población definida con el fin de servir a uno o más propósitos científicos, clínicos o políticos predeterminados.

LRA: Lesión renal aguda

D1.1. ¿Para qué enfermedades o tratamientos existe un registro en su país?

	Si	No	No lo sé
D1.1.1. ERC (sin TRR)	○	○	○

D. Sistemas de información sanitaria y estadísticas
D1. Registros

Definiciones y abreviaturas

Registro: Recopilación sistemática de datos para evaluar resultados específicos en una población definida con el fin de servir a uno o más propósitos científicos, clínicos o políticos predeterminados.

LRA: Lesión renal aguda

D1.1. ¿Para qué enfermedades o tratamientos existe un registro en su país?

D1.1.2. Diálisis ○ ○ ○

D. Sistemas de información sanitaria y estadísticas
D1. Registros

Definiciones y abreviaturas

Registro: Recopilación sistemática de datos para evaluar resultados específicos en una población definida con el fin de servir a uno o más propósitos científicos, clínicos o políticos predeterminados.

LRA: Lesión renal aguda

D1.1. ¿Para qué enfermedades o tratamientos existe un registro en su país?

D1.1.3. Trasplante	○	○	○

D. Sistemas de información sanitaria y estadísticas
D1. Registros

Definiciones y abreviaturas

Registro: Recopilación sistemática de datos para evaluar resultados específicos en una población definida con el fin de servir a uno o más propósitos científicos, clínicos o políticos predeterminados.

LRA: Lesión renal aguda

D1.1. ¿Para qué enfermedades o tratamientos existe un registro en su país?

D1.1.4. LRA	◯	◯	◯

D. Sistemas de información sanitaria y estadísticas
D1. Registros

Definiciones y abreviaturas

Registro: Recopilación sistemática de datos para evaluar resultados específicos en una población definida con el fin de servir a uno o más propósitos científicos, clínicos o políticos predeterminados.

LRA: Lesión renal aguda

D1.1. ¿Para qué enfermedades o tratamientos existe un registro en su país?

D1.1.5. Cuidado conservador ○ ○ ○

D1.2. Si existe un registro de ERC para los pacientes que no necesitan un TRR, ¿en qué se basa la inclusión en el registro de ERC?

- ○ Es voluntaria
- ○ Es obligatoria
- ○ No lo sé

D1.3. Si existe un registro de ERC para los pacientes que no necesitan una TRR, ¿cuál es la cobertura geográfica del registro de ERC? (Marque todas las respuestas adecuadas).

- ☐ Hay un registro nacional
- ☐ Registros regionales/provinciales
- ☐ Registros locales, hospitalarios o de la comunidad

D1.4. Si existe un registro de ERC para los pacientes que no necesitan una TRR, ¿qué abarca? (Marque todas las respuestas adecuadas).

- ○ Todos los grados de la ERC (estadios 1-5)
- ○ Únicamente la ERC avanzada (estadios 4 y 5)

D1.5. Si existe un registro de diálisis, ¿en qué se basa la inclusión en el registro de diálisis?

- ○ Es voluntaria
- ○ Es obligatoria
- ○ No lo sé

D1.6. Si existe un registro de diálisis, ¿cuál es la cobertura geográfica del registro de diálisis? (Marque todas las respuestas adecuadas).

- ☐ Hay un registro nacional
- ☐ Registros regionales/provinciales
- ☐ Registros locales, hospitalarios o de la comunidad

D1.7. Si existe un registro de diálisis, ¿qué información se recopila en el registro de diálisis? (Marque todas las respuestas adecuadas).

- ☐ Etiología de la ERC estadio 5
- ☐ Modalidad de diálisis
- ☐ Prescripción de diálisis
- ☐ Acceso a diálisis (p. ej., acceso vascular para HD, catéter de DP)
- ☐ Mediciones basadas en procesos (p. ej., anemia, enfermedad ósea, marcadores de control de la TA)
- ☐ Medidas de resultados de los pacientes (p. ej., hospitalizaciones)
- ☐ Medidas de resultados de los pacientes (p. ej., satisfacción, calidad de vida)
- ☐ Medidas de resultados de los pacientes (p. ej., mortalidad)

D1.8. Si existe un registro de trasplantes, ¿en qué se basa la inclusión en el registro de trasplantes?	◯ Es voluntaria<br>◯ Es obligatoria<br>◯ No lo sé
D1.9. Si existe un registro de trasplantes, ¿cuál es la cobertura geográfica del registro de trasplantes? (Marque todas las respuestas adecuadas).	☐ Hay un registro nacional<br>☐ Registros regionales/provinciales<br>☐ Registros locales, hospitalarios o de la comunidad
D1.10. Si existe un registro de trasplantes, ¿qué información se recopila en el registro de trasplantes? (Marque todas las respuestas adecuadas).	☐ Etiología de la ERC estadio 5<br>☐ Origen del órgano (donante vivo o cadáver)<br>☐ Tipo de inmunosupresión<br>☐ Episodios de rechazo<br>☐ Tipos y episodios de infección<br>☐ Medidas de resultados de los pacientes (p. ej., hospitalizaciones)<br>☐ Medidas de resultados de los pacientes (p. ej., satisfacción, calidad de vida)<br>☐ Medidas de resultados de los pacientes (p. ej., mortalidad)
D1.11. Si existe un registro de LRA, ¿en qué se basa la inclusión en el registro de LRA?	◯ Es voluntaria<br>◯ Es obligatoria<br>◯ No lo sé
D1.12. Si existe un registro de LRA, ¿cuál es la cobertura geográfica del registro de LRA? (Marque todas las respuestas adecuadas).	☐ Hay un registro nacional<br>☐ Registros regionales/provinciales<br>☐ Registros locales, hospitalarios o de la comunidad
D1.13. Si existe un registro de LRA, ¿qué cubre? (Marque todas las respuestas adecuadas).	◯ Todos los grados de la LRA (estadios 1-3)<br>◯ LRA que requiere terapia de reemplazo renal
D1.14. Si existe un registro de LRA, ¿qué información se recopila en el registro de LRA? (Marque todas las respuestas adecuadas).	☐ Factores de riesgo para la LRA<br>☐ Etiología de la LRA<br>☐ Incidencia de LRA<br>☐ Medidas de resultados de los pacientes (hospitalizaciones)<br>☐ Medidas de resultados de los pacientes (necesidad de TRR, por ejemplo, diálisis o terapias de diálisis lenta como TRRC)<br>☐ Medidas de resultados de los pacientes (mortalidad)

D2. Identificación de la enfermedad (LRA y ERC)
Definiciones:

Directrices: modelos de actuación basados en datos probatorios para la prevención o el tratamiento de la enfermedad.
Identificación: medidas adoptadas en poblaciones en riesgo a fin de diagnosticar a las personas que presentan factores de riesgo o están en estadios iniciales de la enfermedad, pero aún no tienen síntomas.
Política: decisión o conjunto de decisiones oficiales específicas concebidas para llevar a cabo un modelo de actuación avalado por un organismo estatal; incluye un conjunto de objetivos, prioridades e indicaciones generales para alcanzar dichos objetivos. El documento en el que se inscribe la política puede incluir una estrategia para ponerla en práctica.
Programa: conjunto de actividades o procedimientos planificados con un propósito específico.

D2.1. ¿En cuáles de los siguientes grupos de alto riesgo realizan los médicos de su país pruebas sistemáticas para el diagnóstico de la ERC? (Marque todas las respuestas adecuadas).

- ☐ Personas con hipertensión arterial
- ☐ Personas con diabetes
- ☐ Personas con enfermedades cardiovasculares (cardiopatía isquémica, ACV, vasculopatía periférica, insuficiencia cardíaca)
- ☐ Personas con enfermedades autoinmunitarias o multisistémicas (lupus eritematoso sistémico, artritis reumatoide)
- ☐ Ancianos
- ☐ Personas con trastornos urológicos (estructurales, litiasis)
- ☐ Usuarios de medicamentos nefrotóxicos a largo plazo
- ☐ Miembros de grupos étnicos de alto riesgo (aborígenes, africanos, indoasiáticos)
- ☐ Personas con antecedentes familiares de ERC
- ☐ N/A (no aplica); no se hacen pruebas sistemáticas para la ERC

D2.2. ¿Hay en su país grupos étnicos en los que se considere que existe un mayor riesgo de ERC?

- ○ Sí (especifique a continuación)
- ○ No
- ○ No lo sé

D2.2. Si ha seleccionado "si" por favor especifique.

____

D2.3. ¿Existe en su país algún programa de detección de la ERC vigente que se base en políticas o guías nacionales?

- ○ Si
- ○ No
- ○ No lo sé

D2.3.1. Si existe un programa, indique cómo se pone en práctica (marque todas las respuestas adecuadas):

- ○ Enfoque reactivo: se tratan los casos cuando se identifican en la práctica clínica
- ○ Tamizaje activo de la población en riesgo mediante chequeos sistemáticos
- ○ Tamizaje activo de la población en riesgo mediante procesos específicos de detección
- ○ Otros (especifique)

D2.3.1. Si ha seleccionado "otros" por favor especifique.

____

D2.4. ¿Hay en su país grupos específicos en los que se considere que existe un mayor riesgo de LRA?	◯ Sí (especifique a continuación)<br>◯ No<br>◯ No lo sé
D2.4. Si ha seleccionado "si" por favor especifique.	__________________________________
D2.5. ¿Existe en su país algún programa de detección de la LRA vigente que se base en políticas y/o guías nacionales?	◯ Si<br>◯ No<br>◯ No lo sé
D2.5.1. Si existe un programa de detección de LRA, ¿cómo se aplica? (marque todas las respuestas adecuadas):	◯ Enfoque reactivo: se tratan los casos cuando se identifican en la práctica clínica<br>◯ Tamizaje activo de la población en riesgo mediante chequeos sistemáticos<br>◯ Tamizaje activo de la población en riesgo mediante procesos específicos de detección<br>◯ Alertas electrónicas<br>◯ Otros (especifique)
D2.5.1. Si ha seleccionado "otros" por favor especifique.	__________________________________
D3. ¿Disponen de mecanismos para garantizar la validez y calidad de los datos contenidos en los sistemas de información sanitaria?	◯ Si<br>◯ No<br>◯ No lo sé

D4. Capacidad de identificación y tratamiento de la ERC

D4.1. Indique la disponibilidad de los siguientes servicios de control y tratamiento de la ERC en el nivel de atención PRIMARIA en su país

	Disponible	No disponible
D4.1.1. Medición de la tensión arterial	○	○

D4. Capacidad de identificación y tratamiento de la ERC

D4.1. Indique la disponibilidad de los siguientes servicios de control y tratamiento de la ERC en el nivel de atención PRIMARIA en su país

D4.1.2. Medidas de estatura y peso para calcular el índice de masa corporal	○	○

D4. Capacidad de identificación y tratamiento de la ERC

D4.1. Indique la disponibilidad de los siguientes servicios de control y tratamiento de la ERC en el nivel de atención PRIMARIA en su país

D4.1.3. Determinación de la glucosa en suero	○	○

D4. Capacidad de identificación y tratamiento de la ERC

D4.1. Indique la disponibilidad de los siguientes servicios de control y tratamiento de la ERC en el nivel de atención PRIMARIA en su país

D4.1.4. Prueba de HbA1C ○ ○

D4. Capacidad de identificación y tratamiento de la ERC

D4.1. Indique la disponibilidad de los siguientes servicios de control y tratamiento de la ERC en el nivel de atención PRIMARIA en su país

D4.1.5. Determinación del colesterol sérico	○	○

D4. Capacidad de identificación y tratamiento de la ERC

D4.1. Indique la disponibilidad de los siguientes servicios de control y tratamiento de la ERC en el nivel de atención PRIMARIA en su país

D4.1.6. Determinación de la creatinina sérica sin notificación automática de la FGe	○	○

D4. Capacidad de identificación y tratamiento de la ERC

D4.1. Indique la disponibilidad de los siguientes servicios de control y tratamiento de la ERC en el nivel de atención PRIMARIA en su país

D4.1.7. Determinación de la creatinina sérica con notificación automática de la FGe	○	○

D4. Capacidad de identificación y tratamiento de la ERC

D4.1. Indique la disponibilidad de los siguientes servicios de control y tratamiento de la ERC en el nivel de atención PRIMARIA en su país

D4.1.8. Análisis de orina con tiras reactivas para albúmina/proteínas (análisis cualitativos)	○	○

D4. Capacidad de identificación y tratamiento de la ERC

D4.1. Indique la disponibilidad de los siguientes servicios de control y tratamiento de la ERC en el nivel de atención PRIMARIA en su país

D4.1.9. Análisis de orina con tiras reactivas para albúmina/proteínas (análisis cuantitativos)	○	○

D4. Capacidad de identificación y tratamiento de la ERC

D4.1. Indique la disponibilidad de los siguientes servicios de control y tratamiento de la ERC en el nivel de atención PRIMARIA en su país

D4.1.10. Determinación del cociente albúmina:creatinina (CACO) o el cociente proteínas:creatinina en orina (CPCO)	○	○

D4.2. Indique la disponibilidad de los siguientes servicios de control y tratamiento de la ERC en el nivel de asistencia SECUNDARIA O TERCIARIA en su país.

	Disponible	No disponible
D4.2.1. Medición de la tensión arterial	○	○

D4.2. Indique la disponibilidad de los siguientes servicios de control y tratamiento de la ERC en el nivel de asistencia SECUNDARIA O TERCIARIA en su país.

D4.2.2. Medidas de estatura y peso para calcular el índice de masa corporal	○	○

D4.2. Indique la disponibilidad de los siguientes servicios de control y tratamiento de la ERC en el nivel de asistencia SECUNDARIA O TERCIARIA en su país.

D4.2.3. Determinación de la glucosa en suero	○	○

D4.2. Indique la disponibilidad de los siguientes servicios de control y tratamiento de la ERC en el nivel de asistencia SECUNDARIA O TERCIARIA en su país.

D4.2.4. Prueba de HbA1C	○	○

D4.2. Indique la disponibilidad de los siguientes servicios de control y tratamiento de la ERC en el nivel de asistencia SECUNDARIA O TERCIARIA en su país.

D4.2.5. Determinación del colesterol sérico	○	○

D4.2. Indique la disponibilidad de los siguientes servicios de control y tratamiento de la ERC en el nivel de asistencia SECUNDARIA O TERCIARIA en su país.

D4.2.6. Determinación de la creatinina sérica sin notificación automática de la FGe	○	○

D4.2. Indique la disponibilidad de los siguientes servicios de control y tratamiento de la ERC en el nivel de asistencia SECUNDARIA O TERCIARIA en su país.

D4.2.7. Determinación de la creatinina sérica con notificación automática de la FGe	○	○

D4.2. Indique la disponibilidad de los siguientes servicios de control y tratamiento de la ERC en el nivel de asistencia SECUNDARIA O TERCIARIA en su país.

D4.2.8.Análisis de orina con tiras reactivas para albúmina/proteínas (análisis cualitativos)	◯	◯

D4.2. Indique la disponibilidad de los siguientes servicios de control y tratamiento de la ERC en el nivel de asistencia SECUNDARIA O TERCIARIA en su país.

D4.2.9. Análisis de orina con tiras reactivas para albúmina/proteínas (análisis cuantitativos)	◯	◯

D4.2. Indique la disponibilidad de los siguientes servicios de control y tratamiento de la ERC en el nivel de asistencia SECUNDARIA O TERCIARIA en su país.

D4.2.10. Determinación del cociente albúmina:creatinina (CACO) o el cociente proteínas:creatinina en orina (CPCO)	○	○

D4.2. Indique la disponibilidad de los siguientes servicios de control y tratamiento de la ERC en el nivel de asistencia SECUNDARIA O TERCIARIA en su país.		
D4.2.11. Servicios de radiología (p. ej., instalaciones para ecografía renal)	○	○

D4.2. Indique la disponibilidad de los siguientes servicios de control y tratamiento de la ERC en el nivel de asistencia SECUNDARIA O TERCIARIA en su país.

D4.2.12. Servicios de anatomía patología (instalaciones de interpretación de biopsias renales)	○	○

D5: ERC de origen desconocido y poblaciones desproporcionadamente afectadas por ERC

Esta sección sirve para saber si existen puntos críticos regionales de enfermedad renal (en concreto, ERC de origen desconocido) en su país.

D.5.1. ¿Sospecha que existen variaciones regionales en la tasa de enfermedad renal en su país? (Con esto nos referimos a grupos de población con alto riesgo de insuficiencia renal que requieren diálisis o trasplante, o personas que mueren de insuficiencia renal)

- ◯ Sí (por favor, especifique)
- ◯ No

D5.1. Si ha seleccionado "si" por favor especifique.

____

D.5.2. Grupos de edad afectados (seleccione todas las opciones que correspondan):

- ☐ < 18 años de edad
- ☐ 18-44 años de edad
- ☐ 45-64 años de edad
- ☐ Más de 65 años

D.5.3 En su opinión, ¿cuáles son algunas de las causas probables o contribuyentes a la enfermedad renal en estas áreas (seleccione todas las opciones que correspondan)?

- ☐ Diabetes
- ☐ Obesidad
- ☐ Tensión arterial alta
- ☐ Ambientales (p. ej., agua, suelo)
- ☐ Clima (por ejemplo, temperatura alta)
- ☐ Genéticas
- ☐ Biológicas (p. ej., prevalencia alta de VIH o TB, cálculos)
- ☐ Culturales (p. ej., dieta, uso de AINE, hierbas)
- ☐ Otras (especifique)

D.5.3. Si ha seleccionado "otras" por favor especifique.

____

D.5.4. Tipos de industria en esta región (seleccione todas las opciones que correspondan):

- ☐ Agricultura
- ☐ Industria manufacturera
- ☐ Minería
- ☐ Turismo
- ☐ Servicios/Profesionales
- ☐ Otras (especifique)

D.5.4. Si ha seleccionado "otras" por favor especifique.

____

D.5.5. Tipo predominante del clima en su país (seleccione solo uno)

- ◯ Tropical
- ◯ Semiárido
- ◯ Templado
- ◯ Otro (especifique)

D.5.5. Si ha seleccionado "otro" por favor especifique.

____

D.5.6. ¿Cuál es la altitud de este país?	◯ Alta<br>◯ A nivel del mar<br>◯ Baja<br>◯ Otra (por favor, aporte detalles)
D.5.6. Si ha seleccionado "otra" por favor especifique.	__________________________________

E. Política sanitaria nacional
Definiciones:
Política: decisión o conjunto de decisiones oficiales específicas concebidas para llevar a cabo un modelo de actuación avalado por un organismo estatal; incluye un conjunto de objetivos, prioridades e indicaciones generales para alcanzar dichos objetivos. El documento en el que se inscribe la política puede incluir una estrategia para ponerla en práctica.
Programa: conjunto de actividades o procedimientos planificados con un propósito específico.
Estrategia: plan a largo plazo concebido para lograr un fin concreto.

E1. Política y estrategia
Enfermedades no transmisibles (ENT): enfermedades que no se pueden contagiar a otra persona, en especial las enfermedades cardiovasculares (como el infarto de miocardio y el ACV), el cáncer, las enfermedades respiratorias crónicas (como la EPOC y el asma) y la diabetes.

E1.1. ¿Existe en su país alguna estrategia nacional para las enfermedades no transmisibles?	◯ Sí, en vigor (indique los detalles a continuación)<br>◯ Está en desarrollo, pero aún no se ha puesto en práctica (indique los detalles a continuación)<br>◯ No<br>◯ No lo sé
E1.1. Si ha seleccionado "si" por favor especifique.	__________________________________
E1.1. Si está en fase de desarrollo, por favor, aporte detalles al respecto.	__________________________________
E1.2. ¿Existe en su país alguna estrategia nacional para mejorar la atención sanitaria a los pacientes con ERC?	◯ Sí, existe una estrategia nacional específica para la ERC<br>◯ Sí, pero la estrategia para la ERC está dentro de una estrategia para ENT que incluye otras patologías<br>◯ No<br>◯ No lo sé
E1.2.1. Indique las poblaciones cubiertas por la estrategia nacional específica para la ERC (marque todas las respuestas adecuadas)	☐ ERC sin diálisis<br>☐ Diálisis crónica<br>☐ Trasplante de riñón
E1.2.2. Indique las poblaciones cubiertas por la estrategia nacional general para las ENT (marque todas las respuestas adecuadas)	☐ ERC sin diálisis<br>☐ Diálisis crónica<br>☐ Trasplante de riñón
E1.3. ¿Existen políticas específicas para la ERC?	◯ Si<br>◯ No<br>◯ No lo sé
E1.3.1. Si la respuesta es afirmativa, especifique el tipo de políticas para la ERC que existen en su país (marque todas las respuestas adecuadas)	☐ Políticas nacionales<br>☐ Políticas regionales

E2. Sensibilización

E2.1. En su opinión, ¿las autoridades de su país reconocen la ERC como una prioridad sanitaria?	◯ Sí (indique los detalles a continuación)<br>◯ No (explique por qué no a continuación)
E2.1. Si ha seleccionado "si" por favor especifique.	__________________________________
E2.1. Si ha seleccionado "no" por favor especifique.	__________________________________
E2.2. ¿Existe algún grupo de sensibilización en los niveles máximos del estado (por ejemplo, una comisión parlamentaria) o alguna ONG para dar un mayor relieve a la ERC y su prevención?	◯ Sí (indique los detalles a continuación)<br>◯ No (explique por qué no a continuación)<br>◯ No lo sé
E2.2. Si ha seleccionado "si" por favor especifique.	__________________________________
E2.2. Si ha seleccionado "no" por favor especifique.	__________________________________
E2.3. En su opinión, ¿las autoridades de su país reconocen la LRA y/o su prevención como una prioridad sanitaria?	◯ Sí (indique los detalles a continuación)<br>◯ No (explique por qué no a continuación)
E2.3. Si ha seleccionado "si" por favor especifique.	__________________________________
E2.3. Si ha seleccionado "no" por favor especifique.	__________________________________
E2.4. ¿Existe algún grupo de sensibilización en los niveles máximos del estado (por ejemplo, una comisión parlamentaria) o alguna ONG para dar un mayor relieve a la LRA y su prevención?	◯ Sí (indique los detalles a continuación)<br>◯ No (explique por qué no a continuación)<br>◯ No lo sé
E2.4. Si ha seleccionado "si" por favor especifique.	__________________________________
E2.4. Si ha seleccionado "no" por favor especifique.	__________________________________
E2.5. En su opinión, ¿las autoridades de su país reconocen la ERC estadio 5 y/o el TRR como una prioridad sanitaria?	◯ Sí (indique los detalles a continuación)<br>◯ No (explique por qué no a continuación)
E2.5. Si ha seleccionado "si" por favor especifique.	__________________________________

E2.5. Si ha seleccionado "no" por favor especifique.

____

E2.6. ¿Existe algún grupo de sensibilización en los niveles máximos del estado (por ejemplo, una comisión parlamentaria) o alguna ONG para dar un mayor relieve a la ERC estadio 5 o el TRR?

- ◯ Sí (indique los detalles a continuación)
- ◯ No (explique por qué no a continuación)
- ◯ No lo sé

E2.6. Si ha seleccionado "si" por favor especifique.

____

E2.6. Si ha seleccionado "no" por favor especifique.

____

E2.7. ¿Existen organizaciones nacionales o regionales para los médicos u organizaciones de pacientes que faciliten recursos para la atención sanitaria de la ERC estadio 5?

- ◯ Sí (indique los detalles a continuación)
- ◯ No (explique por qué no a continuación)
- ◯ No lo sé

E2.7. Si ha seleccionado "si" por favor especifique.

____

E2.7. Si ha seleccionado "no" por favor especifique.

____

E3. Obstáculos en la atención sanitaria óptima de las enfermedades renales

E3.1. ¿Existen en su país obstáculos específicos para una atención sanitaria óptima de las enfermedades renales? Marque todas las respuestas adecuadas.

- ☐ Geografía (distancia a los centros asistenciales o desplazamientos prolongados)
- ☐ Médico (disponibilidad, acceso, conocimientos, actitud)
- ☐ Paciente (conocimientos, actitud)
- ☐ Nefrólogo (disponibilidad)
- ☐ Sistema sanitario (disponibilidad, acceso, recursos)
- ☐ Ausencia de voluntad política y de políticas facilitadoras
- ☐ Factores económicos (financiación limitada, mecanismos del reembolso deficientes)
- ☐ Otra (especifique)

E3.1. Si ha seleccionado "otra" por favor especifique.

____

E4. ¿Cómo recopiló la información para cumplimentar esta encuesta? Marque todas las respuestas adecuadas.

- ☐ Opinión o conocimientos personales
- ☐ Recopilé información de otras fuentes (por ejemplo, literatura médica o informes publicados)
- ☐ Consulté con otros colegas
- ☐ Otra (especifique)

E4. Si ha seleccionado "otra" por favor especifique.

____
