# Supplementary material for: A global snapshot on health systems capacity for detection, monitoring, and management of acute kidney injury: A multinational study from the ISN-GKHA
Source: PLOS Glob Public Health. 2024 Oct 15;4(10):e0003823. doi: 10.1371/journal.pgph.0003823 (PMC11478907; doi:10.1371/journal.pgph.0003823)
Supplement: S2 Fig — (PDF) [file pgph.0003823.s004.pdf]

S2 Fig. Countries with AKI detection programs based on national policy or guidelines, by ISN regions and World Bank income groups.*

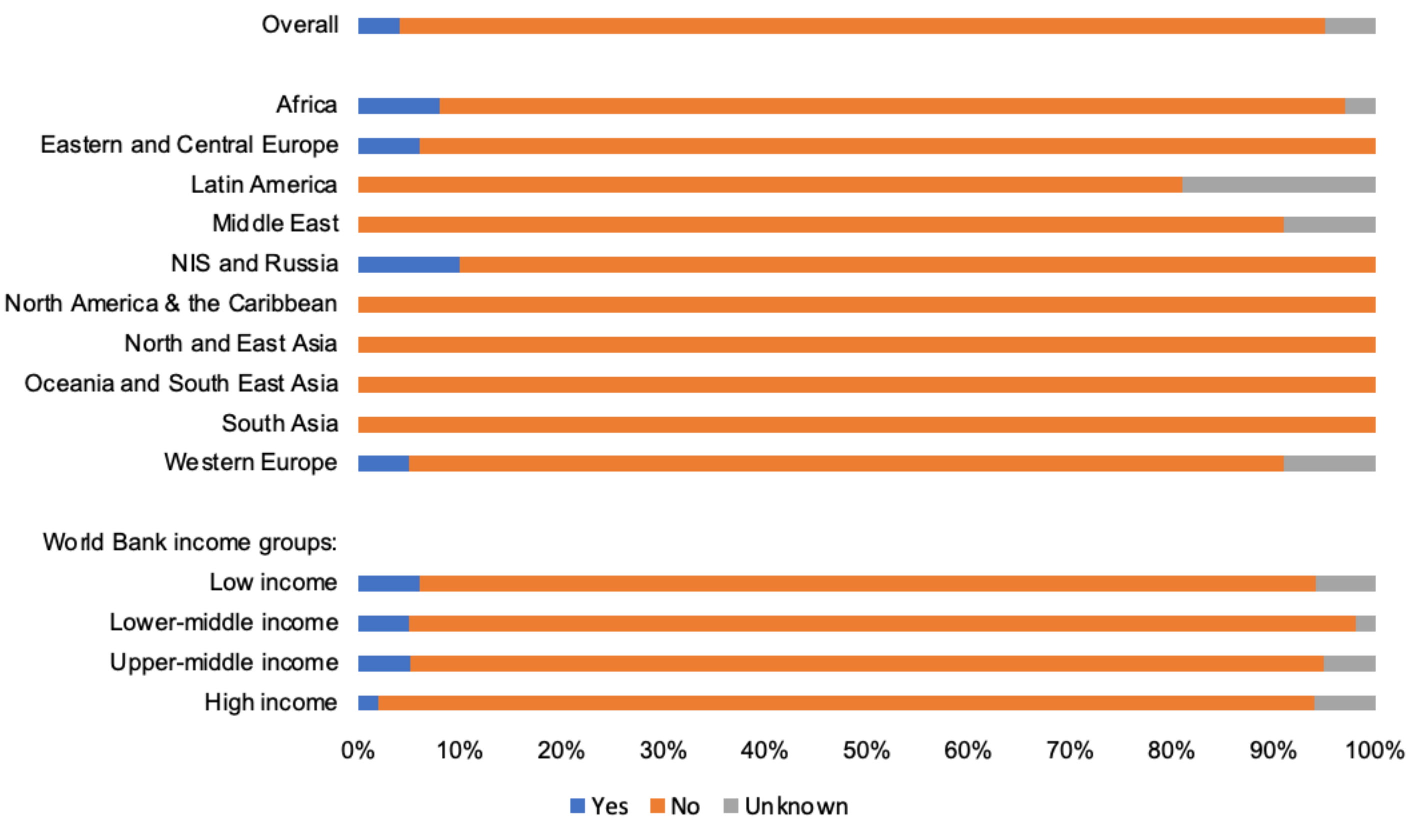

*Values represent absolute number of countries in each category expressed as a percentage of total number of countries.
Abbreviations: AKI = acute kidney injury; ISN = International Society of Nephrology; NIS = Newly Independent States
