# Supplementary material for: A global snapshot on health systems capacity for detection, monitoring, and management of acute kidney injury: A multinational study from the ISN-GKHA
Source: PLOS Glob Public Health. 2024 Oct 15;4(10):e0003823. doi: 10.1371/journal.pgph.0003823 (PMC11478907; doi:10.1371/journal.pgph.0003823)
Supplement: S1 Table — (DOCX) [file pgph.0003823.s008.docx]

**S1 Table. Characteristics of existing AKI registries, by ISN regions and World Bank income groups (N, %).**

|  | **Provider Participation** | | | **Geographical Coverage** | | | **Spectrum of AKI covered** | | **Information collated in the AKI registry** | | | | | |
| --- | --- | --- | --- | --- | --- | --- | --- | --- | --- | --- | --- | --- | --- | --- |
|  | **Voluntary** | **Mandatory** | **Unknown** | **National** | **Regional/ state/ provincial** | **Local/ hospital/ community** | **Whole spectrum of AKI (stages 1-3)** | **AKI requiring KRT** | **Risk factors for AKI** | **Etiology of AKI** | **Incidence of AKI** | **Hospitalization** | **Requirement for KRT** | **Mortality** |
| Overall | 5 (36) | 6 (43) | 3 (21) | 7 (50) | 1 (7) | 5 (36) | 10 (71) | 4 (29) | 7 (50) | 9 (64) | 9 (64) | 7 (50) | 6 (43) | 7 (50) |
| ISN regions: |  |  |  |  |  |  |  |  |  |  |  |  |  |  |
| Africa | 1 (25) | 2 (50) | 1 (25) | 0 | 0 | 2 (50) | 2 (50) | 2 (50) | 2 (50) | 2 (50) | 2 (50) | 1 (25) | 1 (25) | 2 (50) |
| Eastern and Central Europe | 0 | 1(50) | 1 (50) | 1 (50) | 0 | 1 (50) | 1 (50) | 1 (50) | 1 (50) | 2 (100) | 1 (50) | 2 (100) | 1 (50) | 2 (100) |
| Latin America | 2 (67) | 1(33) | 0 | 3 (100) | 0 | 0 | 3 (100) | 0 | 3 (100) | 3 (100) | 3 (100) | 2 (67) | 2 (67) | 1 (33) |
| Middle East | 0 | 0 | 0 | 0 | 0 | 0 | 0 | 0 | 0 | 0 | 0 | 0 | 0 | 0 |
| NIS and Russia | 1 (50) | 1(50) | 0 | 2 (100) | 1 (50) | 1 (50) | 1 (50) | 1 (50) | 0 | 0 | 2 (100) | 1 (50) | 1 (50) | 1 (50) |
| North America and the Caribbean | 0 | 1(100) | 0 | 0 | 0 | 1 (100) | 1 (100) | 0 | 0 | 1 (100) | 0 | 0 | 0 | 1 (100) |
| North and East Asia | 0 | 0 | 0 | 0 | 0 | 0 | 0 | 0 | 0 | 0 | 0 | 0 | 0 | 0 |
| Oceania and South East Asia | 0 | 0 | 0 | 0 | 0 | 0 | 0 | 0 | 0 | 0 | 0 | 0 | 0 | 0 |
| South Asia | 0 | 0 | 0 | 0 | 0 | 0 | 0 | 0 | 0 | 0 | 0 | 0 | 0 | 0 |
| Western Europe | 1 (50) | 0 | 1 (50) | 1 (50) | 0 | 0 | 2 (100) | 0 | 1 (50) | 1 (50) | 1 (50) | 1 (50) | 1 (50) | 0 |
| World Bank income groups: |  |  |  |  |  |  |  |  |  |  |  |  |  |  |
| Low income | 1 (33) | 2 (67) | 0 | 0 | 0 | 2 (67) | 2 (67) | 1 (33) | 2 (67) | 2 (67) | 2 (67) | 1 (33) | 1 (33) | 2 (67) |
| Lower-middle income | 0 | 1 (100) | 0 | 1 (100) | 1 (100) | 1 (100) | 1 (100) | 0 | 0 | 0 | 1 (100) | 1 (100) | 1 (100) | 1 (100) |
| Upper-middle income | 3 (50) | 2 (33) | 1 (17) | 4 (67) | 0 | 1 (17) | 4 (67) | 2 (33) | 3 (50) | 4 (67) | 4 (67) | 2 (33) | 1 (17) | 2 (33) |
| High income | 1 (25) | 1 (25) | 2 (50) | 2 (50) | 0 | 1 (25) | 3 (75) | 1 (25) | 2 (50) | 3 (75) | 2 (50) | 3 (75) | 3 (75) | 2 (50) |

Abbreviations: AKI – acute kidney injury; ISN – International Society of Nephrology; KRT – kidney replacement therapy; NIS = Newly Independent States
